# New York State Climate Impacts Assessment Chapter 10: Water Resources

**DOI:** 10.1111/nyas.15197

**Published:** 2024-12-09

**Authors:** Kelsey Leonard, Stephen B. Shaw, Abraham Francis, David Hermann, Laureline Josset, Christine L. May, Benjamen Wright, Kiyoko Yokota, Amanda Stevens

**Affiliations:** ^1^ University of Waterloo Waterloo Ontario Canada; ^2^ Department of Environmental Resources Engineering The State University of New York College of Environmental Science and Forestry Syracuse New York USA; ^3^ Department of Infrastructure, Housing, and Environment Mohawk Council of Akwesasne Akwesasne Ontario Canada; ^4^ United States Department of State (Ret.) Clinton New York USA; ^5^ Columbia Water Center Columbia University New York New York USA; ^6^ Pathways Climate Institute San Francisco California USA; ^7^ Hazen and Sawyer Baltimore Maryland USA; ^8^ Biology Department State University of New York Oneonta Oneonta New York USA; ^9^ New York State Energy Research and Development Authority Albany New York USA

**Keywords:** adaptation, climate change, groundwater, impacts, infrastructure, New York State, resilience, surface water, vulnerability, water

## Abstract

Clean, abundant water is essential to the health of New York State's residents, ecosystems, and economy. This critical natural resource faces numerous challenges associated with climate change, including potential impacts on the quality and quantity of source waters—both surface waters and groundwater. Climate change can also affect the infrastructure that treats and delivers safe drinking water to New Yorkers, manages wastewater to protect water quality, and protects against flooding. The Water Resources chapter examines these impacts and highlights opportunities for New Yorkers to adapt and build resilience as the state's climate changes.

## 1 TECHNICAL WORKGROUP KEY FINDINGS

Clean, abundant water is essential to the health of New York State's residents, ecosystems, and economy. This critical natural resource faces numerous challenges associated with climate change, including potential impacts on the quality and quantity of source waters—both surface waters and groundwater. Climate change can also affect the infrastructure that treats and delivers safe drinking water to New Yorkers, manages wastewater to protect water quality, and protects against flooding. The Water Resources chapter examines these impacts and highlights opportunities for New Yorkers to adapt and build resilience as the state's climate changes.

**Key Finding 1: Water bodies and groundwater under the direct influence of sea level are already being affected by climate change and will face greater risks in the future**. Groundwater wells in coastal areas will be subject to saltwater intrusion from sea level rise. Rising seas will also push the saltwater boundary on estuarial rivers such as the Hudson River farther upstream during drought periods, potentially affecting freshwater use and withdrawals. Detailed modeling of saltwater intrusion and estuary salt concentrations will lead to a better understanding of likely impacts.

**Key Finding 2: New York State's aging water infrastructure is particularly vulnerable to the impacts of climate change**. New York's older infrastructure was built for water levels, flows, and water quality conditions that have since changed and will continue to change. Older infrastructure is also more prone to leaks, breaks, and other failures. Upgrades to aging infrastructure can offer the dual benefit of building resilience for the future and providing immediate improvements in water system function. Strategically identifying upgrades that also support climate adaptation can be a cost‐effective way to allocate limited resources.

**Key Finding 3: Resources and preparedness for dealing with climate change vary greatly depending on the size and wealth of different communities**. Water infrastructure and water resources management are largely overseen by local governments. For communities that cannot afford full‐time technical staff, several different resources are available from state and federal governments and nongovernmental organizations, but these resources can be difficult and time‐consuming to navigate. There is a need to develop more efficient and less burdensome ways to support communities in implementing resilience measures.

**Key Finding 4: Long‐term water infrastructure resilience requires proactive incorporation of changing climate conditions into planning and design**. State, Tribal, municipal, and other responsible entities broadly recognize that new infrastructure should reflect knowledge of future climate conditions to avoid increasing risk of failure. However, new analytical tools, models, and data sets need further development to provide a basis for establishing forward‐looking standards. Continued support of applied science research and open‐access water data will help advance these improvements.

**Key Finding 5: Climate change could place new stresses on already complex, multijurisdictional water governance, especially during drought periods**. A variety of in‐state and regional organizations manage these multijurisdictional waters for flood protection water supply, electric generation, recreation, and other uses. These complex governance structures affect underserved communities, regional governments, Tribal Nations, and other entities that are particularly vulnerable to negative climate impacts and that often have fewer resources and influence to represent their interests. Water management approaches that incorporate these groups’ participation and diverse knowledge, including Indigenous Traditional Ecological Knowledge, can help remedy water injustices and identify new opportunities to increase climate resilience.

BOX 1 **Developments since the 2011 ClimAID assessment**

The 2011 ClimAID assessment evaluated risks, vulnerabilities, and potential adaptation strategies in response to ongoing and projected future climate change across New York State.^1^ The Water Resources chapter of the ClimAID assessment looked at how changing climatic conditions would affect four main topic areas: (1) flooding in noncoastal regions, (2) drinking water supply, (3) water availability for nonpotable uses, and (4) water quality. This chapter provides an updated assessment in those areas while also broadening the focus to look more extensively at issues of water governance and how climate change will affect pollution prevention (i.e., wastewater management) infrastructure. It also provides an overview of research areas that have emerged and deepened in the years since ClimAID, including impacts on groundwater in coastal areas, water affordability, changes in water demand, and environmental justice concerns. In addition, this chapter more extensively considers multiple perspectives on climate change, including those of local governments, Indigenous communities, and individual homeowners.

## 2 INTRODUCTION AND BACKGROUND

New Yorkers depend on water for a variety of uses, including drinking water, irrigation, recreation, manufacturing, fisheries, and power generation. Myriad institutions within the state are responsible for planning, constructing, operating, maintaining, and regulating water‐related infrastructure to ensure that surface water and groundwater resources continue to support human and habitat needs. This chapter reviews the best available information sources to quantify the impacts of climate change on New York State's water resources that have been observed to date, impacts projected in the decades ahead, and opportunities to adapt and build resilience. It cites and assesses evidence from technical literature, analyzes data from state and federal agencies and other sources, and integrates perspectives from practitioners who manage water resources and infrastructure in New York.

The background material in this section (Section 2) provides a foundation for understanding the impacts of climate change on water, including an overview of the state's water resources and systems, key climate hazards affecting them, issues of equity and justice, and opportunities for positive change. Subsequent sections assess the state of knowledge on climate impacts and adaptation as follows:

- **Section** **3** examines climate risks and vulnerabilities that relate to the quantity and quality of surface water and groundwater resources in the state. It focuses on surface and groundwater resources that provide potable (drinking) water but notes that many of the same concerns extend to other water uses.
- **Section** **4** examines climate risks and vulnerabilities for the infrastructure used to convey and treat drinking water and wastewater.
- **Section** **5** explores the variety of adaptations that can be used to help address the climate impacts and vulnerabilities experienced by New York State's water resources. The section describes water resource adaptations under three broad categories: structural, nonstructural, and governance.
- **Section** **6** highlights opportunities for positive change that can grow out of climate adaptation efforts and identifies notable emerging topics and research needs in the Water Resources sector. This section also provides a conclusion, summarizing the major findings and recommendations presented in the chapter.
- The **Traceable Accounts** appendix examines each key finding in depth. It provides citations that support each assertion, and it presents the authors’ assessment of confidence in each finding.
- **Case studies** highlight examples of climate change impacts on watersheds, water supply systems, and the communities who depend on them, along with adaptation and resilience strategies that could serve as models for others. These case studies are not included in the chapter proper but are available through links provided in the chapter.

### 2.1 Sector scope and context

This section summarizes New York State's water resources, infrastructure, and demand, along with the institutions that manage these resources.

#### 2.1.1 Overview of New York State's water resources

New York State has 17 major watersheds, as shown in Figure 10-1.^2^ The state has more than 7800 freshwater lakes, ponds, and reservoirs, as well as 52,337 miles of rivers and streams. It also has approximately 117 miles of shoreline stretching along the coast of the Atlantic Ocean, including five major estuaries. In addition, New York has 577 miles of shoreline within the Great Lakes Basin, including shoreline along Lake Ontario, Lake Erie, the St. Lawrence River, and the Niagara River.^3^, ^4^ Wetlands in the state include 2.4 million acres of freshwater wetlands and 25,000 acres of tidal wetlands.^4^

**FIGURE 10-1:**
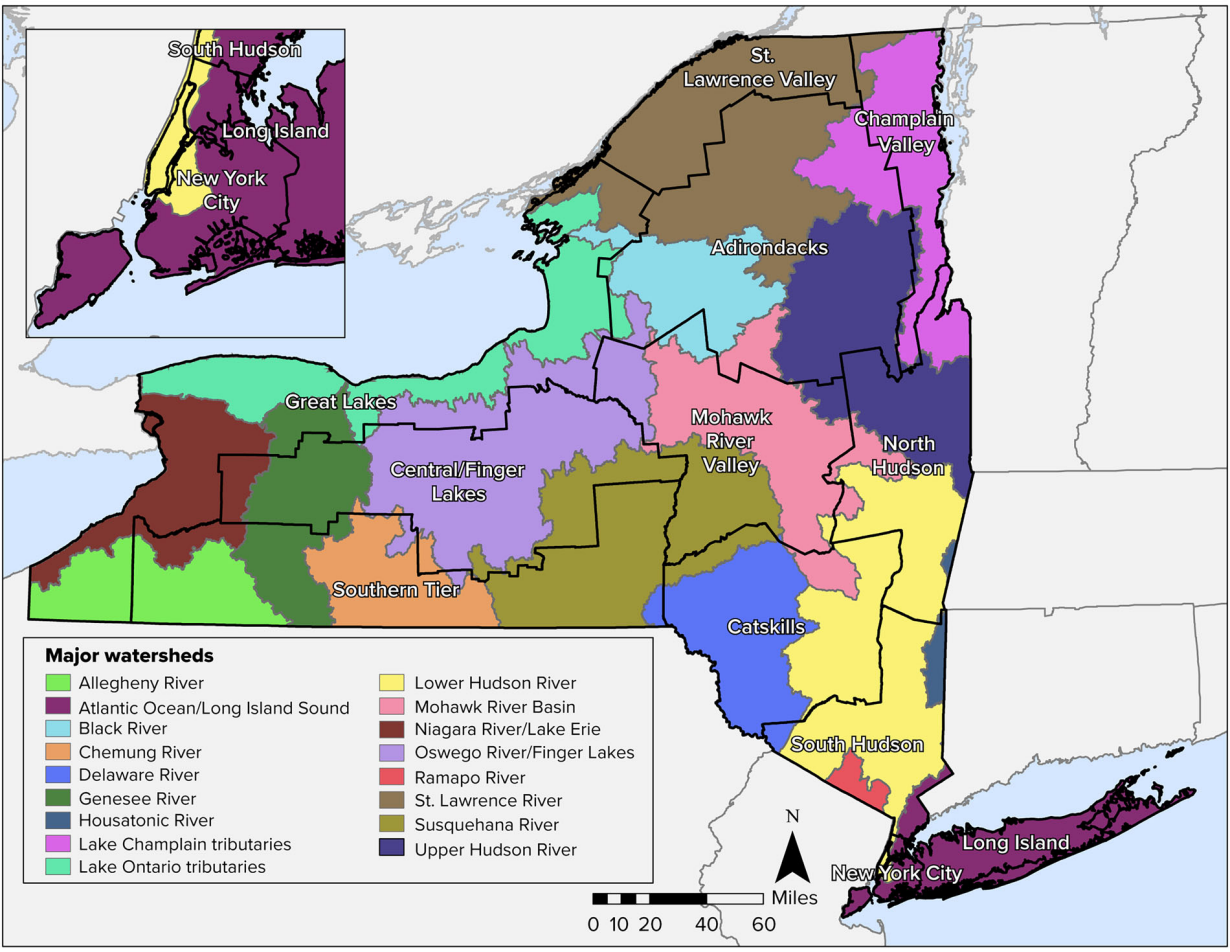
Major watersheds in New York State, with assessment regions overlain. Data from the New York State Department of Environmental Conservation (NYSDEC) (2023).^6^

Areas in the state with geology most suitable for supplying groundwater are designated as primary aquifers and principal aquifers, located as shown in Figure 10-2. Primary aquifers are designated based on current, extensive use by major municipal water supply systems. Principal aquifers can provide a substantial amount of water but are not as heavily used. Together, primary and principal aquifers cover approximately 18% of all New York State land area.^5^, ^6^

**FIGURE 10-2:**
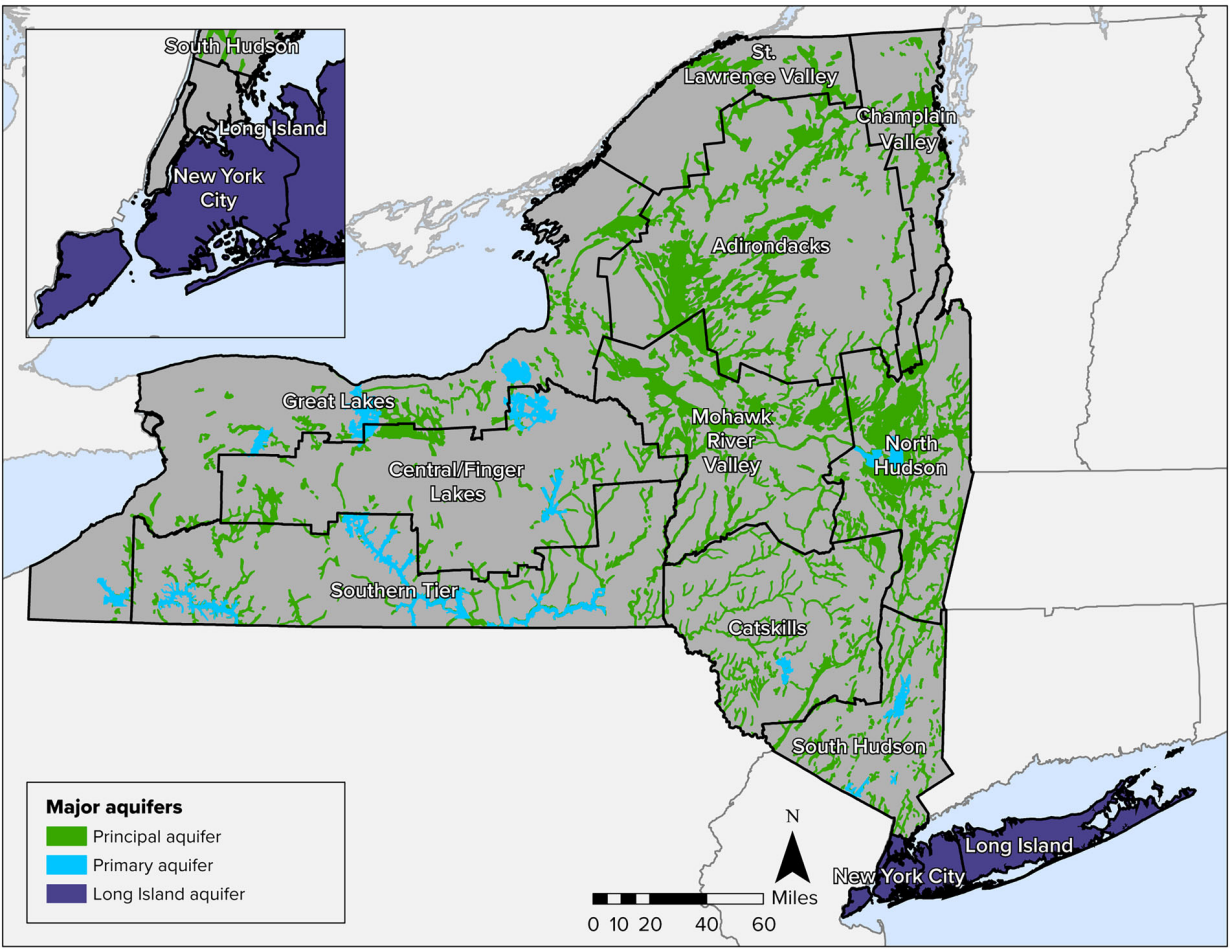
Primary and principal aquifers in New York State. Data from NYSDEC (2023).^7^

Regulations and voluntary programs at all levels of government combine to protect New York's water resources. The New York State Department of Environmental Conservation (NYSDEC) is the lead state agency for monitoring surface water and groundwater quality, administering permits to regulate sources of pollution, and regulating water withdrawals. New York's water quality standards are the foundation for how the state manages programs, enforces regulations, and issues permits to protect surface water and groundwater resources. Each water body has an assigned best use—for example, source of drinking water, primary contact recreation (i.e., swimming), secondary contact recreation (i.e., boating), fishing, and shellfishing. For groundwater resources, the best use is potable water supply. Drinking water standards, established by the New York State Department of Health (NYSDOH), are distinct from source water quality standards, as they pertain to “finished” water delivered to customers after treatment. Responsibility for potable water supply operation and planning is highly fragmented and managed at the individual utility level.

#### 2.1.2 Overview of New York State's water infrastructure

The infrastructure used to manage New York's water resources includes water supply, wastewater management, and flood control infrastructure. Constructing, operating, maintaining, and upgrading the state's water infrastructure is essential to ensuring that water meets water quality and drinking water standards. However, much of that infrastructure is aging and currently or soon to be in need of replacement.^8^ This section summarizes key information about the state's water resources infrastructure.

##### 2.1.2.1 Water supply infrastructure

New Yorkers obtain potable water from a variety of sources (Figure 10-3). Based on 2015 estimates, 12.6 million people receive water from surface sources such as lakes, reservoirs, and rivers.^9^ Surface water sources are largely maintained by public water suppliers such as water utilities or water companies. The largest surface water supplier, the New York City Department of Environmental Protection (NYC DEP), provides water to about 9.5 million users,^10^ while the smallest serves a few hundred people.^9^ The remainder of the state population—about 7.1 million people—receive water from groundwater sources, where wells pump water from underground. About 2.5 million residents get water from private domestic wells. The other 4.6 million users who rely on groundwater sources receive water from public water suppliers; the majority of these users (about 2.8 million) reside on Long Island.^9^ Every water supply system—whether surface or groundwater, public or private—has unique features dependent on geology, hydrology, and regional climate, as well as on the installed infrastructure. Section 3 provides a broad overview of climate impacts on the surface water and groundwater resources found in the state and Tribal Nations.

**FIGURE 10-3:**
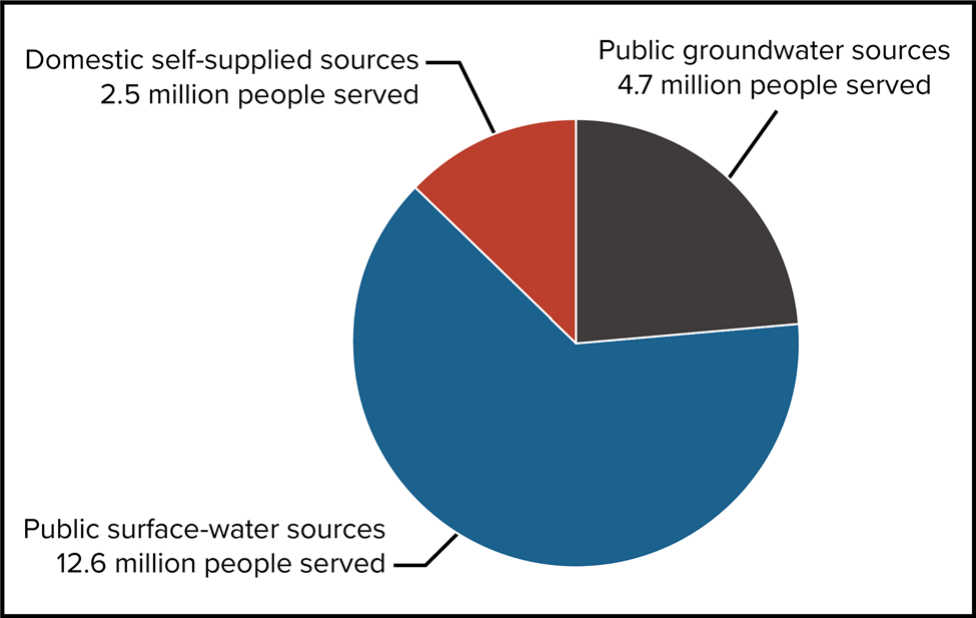
Sources of potable water in New York State (millions of people served), 2015. Data from Dieter et al.^9^

##### 2.1.2.2 Wastewater infrastructure

Pollutants can enter water resources from point sources (i.e., discrete wastewater discharge pipes) and nonpoint sources (i.e., diffuse sources of runoff). Wastewater treatment facilities—also referred to as wastewater resource recovery facilities in some places, including New York City—are point sources and are a critical component of wastewater infrastructure across the state. There are more than 600 wastewater treatment plants (WWTPs) operated by municipalities and water utilities statewide. Facility capacities range from processing less than 100,000 gallons to up to 1.3 billion gallons of wastewater per day.^11^ Approximately 200 additional municipalities own pipe infrastructure that conveys their sewage to other municipally owned wastewater treatment facilities.^12^ More than 35,000 miles of sewers convey wastewater throughout the state. Industrial facilities with wastewater discharges are also point sources that own and operate wastewater infrastructure. More than 600 industrial facilities have wastewater industrial permits that authorize discharges to state water bodies.^13^

Septic systems can also act as a source of pollution. Approximately 22% of homes in the state have septic systems for treating household wastewater.^14^ Septic systems are most common in rural areas but can also often be found in high density around lakes and in coastal areas. Long Island, for example, has more than 400,000 septic systems.^15^

Stormwater systems, both separate and combined, are also important components of the state's wastewater infrastructure. Municipal separate storm sewer systems, referred to as MS4s, convey stormwater runoff and discharge untreated stormwater directly to surface waters. Combined sewer systems (CSSs) convey both sanitary sewage and stormwater; as the volume of wastewater increases during heavy precipitation events, wastewater treatment facilities sometimes exceed capacity, and untreated wastewater discharges to surface waters via combined sewer overflow (CSO) outfalls. New York has approximately 800 CSO outfalls, 74% of which are operated by four permitted communities—New York City, Buffalo, Syracuse, and Albany.^16^ More than 10 million New Yorkers—more than half the state's population—live in communities served by CSSs.^17^

Throughout the state, the infrastructure used to manage nonpoint sources of pollution varies by land use type. In urban settings, best management practices (BMPs) for addressing nonpoint pollution from wet weather runoff include structural measures such as constructed wetlands, green roofs, and rain gardens. In rural areas, BMPs include riparian buffer strips, nutrient management planning, and retention ponds.

When operated and maintained correctly, wastewater treatment facilities, septic systems, and nonpoint source management measures help keep waterways clean. However, most of the state's existing infrastructure was not designed to accommodate changing climatic conditions. Section 4 will explore climate impacts and vulnerabilities related to pollution control systems.

#### 2.1.3 Statewide water uses and demand

In addition to meeting potable water demands, water is used for irrigation, industry, power generation, recreation, cultural and ceremonial practices, and natural habitats for a broad range of species. To give a sense of proportion, New York uses 60 million gallons per day for irrigation during peak growing season, 122 million gallons per day consumptively for thermoelectric power generation, 320 million gallons per day for industry, and more than 2600 million gallons per day for potable water.^9^ Not quantified here is the water that remains in streams and rivers, available for recreation, transportation via waterways, and natural habitats. This remaining water far outweighs the consumed water when considering the state as a whole. However, water limitations can occur during drier periods and on specific water bodies that experience high demand.

#### 2.1.4 Water management institutions and governance

Climate change could lead to increased demand for access to water resources at the local and regional scale, and it will also lead to other challenges such as an increase in floods and short‐term droughts. Managing these stresses is especially challenging for water bodies that cross political boundaries and require coordinated water use and for those that have multiple, sizable users and complex legal issues. A single watershed can extend across numerous small municipalities; a particular challenge is when upstream communities are less inclined to manage water resources that eventually flow to downstream communities. This section provides an overview of several state and regional entities with formal cross‐boundary governance structures or other unique water management responsibilities that have grown more complex in the face of a changing climate. Figure 10-4 identifies these key entities and shows the areas of the state that they cover. Section 5.3 discusses adaptations these entities are using or could use to prepare for the impacts of climate change. Section 2.5 provides more discussion of a particularly important set of governance issues: the relationship between New York State and Tribal Nations in managing water in conjunction with the Tribal Nation water rights established by federal law.

**FIGURE 10-4:**
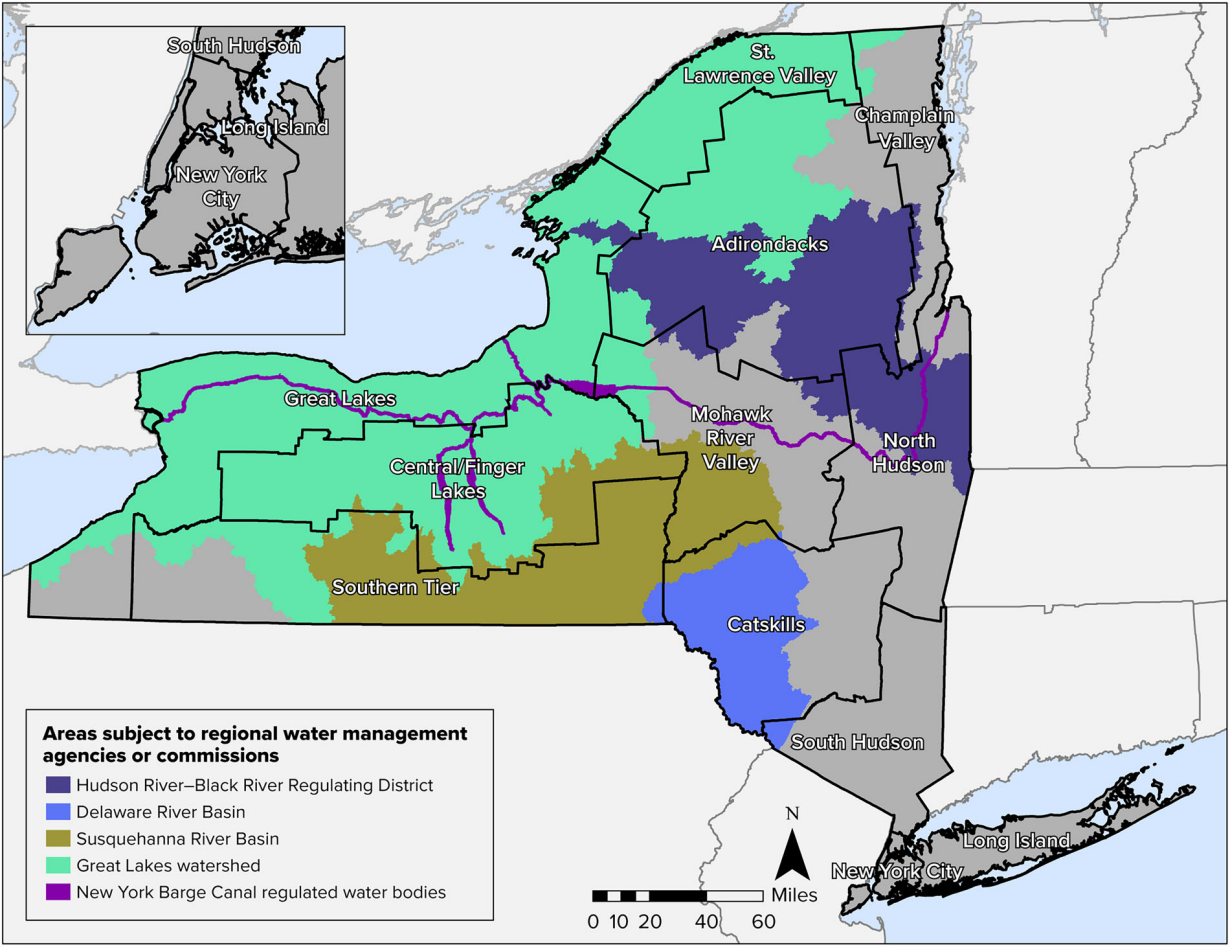
Key entities with cross‐boundary water governance responsibilities in New York, with assessment regions overlain. Data from USGS (2023).^18^

##### 2.1.4.1 Great Lakes and the International Joint Commission

The International Joint Commission (IJC) was established under the Boundary Waters Treaty of 1909 to prevent and resolve disputes between the United States and Canada over transboundary waters. The IJC reviews and approves projects that affect water levels and flows across the boundary and is often tasked by both governments to investigate emerging transboundary water issues and recommend solutions. From the beginning, the IJC has focused on the impacts of flooding, droughts, and extreme precipitation events on boundary waters—an aspect of its work that will grow in importance with climate change.^19^

The IJC comprises three American and three Canadian commissioners, who take an oath to support the Boundary Waters Treaty in an impartial fashion. The commissioners are supported by staff in Washington, D.C.; Ottawa; and Windsor, Ontario. The IJC has also established advisory and control boards to provide local oversight for various shared transboundary waters. Several of these boards engage on boundary waters touching New York State: the International Niagara Board of Control, the International Lake Ontario‐St. Lawrence River Board, the International Lake Champlain‐Richelieu River Study Board, the Great Lakes Water Quality Board, and the Great Lakes Science Advisory Board. Climate change increasingly factors into the work of these boards, and climate impacts are a growing challenge in carrying out their responsibilities assigned by the IJC.^19^

Of the major water planning issues addressed to date by the IJC, the United States‐Canada Great Lakes Water Quality Agreement and the regulation of Lake Ontario and the St. Lawrence River are particularly important to New York State. Based on recommendations developed by the IJC, the United States and Canada signed the Great Lakes Water Quality Agreement in 1972 with the objective of restoring and maintaining the chemical, physical, and biological integrity of the waters of the Great Lakes Basin ecosystem. The agreement was amended in 2012 to address emerging concerns such as aquatic invasive species and the water quality impacts of climate change.^19^

Following the opening of the Moses‐Saunders Dam on the St. Lawrence River in 1958, the IJC began regulating Lake Ontario and the St. Lawrence River in order to reduce flooding to shoreline communities, improve commercial shipping, and generate electricity.^20^ As the ecological impacts of regulating levels and flow became apparent, the U.S. and Canadian governments asked the IJC to develop a new regulation plan that would continue to provide these benefits while also helping to restore a more natural balance of levels and flows. In late 2016, the IJC instituted a new regulation plan called “Plan 2014” that provides technical rules about the timing and quantity of water releases from the dam, as well as rules for deviations during floods or droughts.^20^, ^21^

In 2015, the IJC established the Great Lakes‐St. Lawrence River Adaptive Management Committee to assess hydroclimatic trends in the Great Lakes Basin and develop strategies for transboundary cooperation and coordination.^22^ The 2020 IJC *Triennial Assessment of Progress Report* highlighted the current and persistent impacts of climate change on water resources across the basin, including in New York State and for Tribal Nations.^23^ Moreover, the IJC Great Lakes Water Quality Board's 2021 Great Lakes Regional Poll found that 76% of the 4550 respondents are highly concerned with the negative impacts of climate change on water quality in the Great Lakes.^24^

##### 2.1.4.2 Delaware and Susquehanna River Basin Commissions

Two extensively used regional river systems originate in New York and then flow into other states: the Susquehanna River and the Delaware River. Since the 1960s, actions have been taken in both basins to create interstate compacts that address water management concerns.^25^

Dams on the headwater sections of the Delaware River create reservoirs that supply a substantial portion of New York City's water, while water in the lower portion is withdrawn to supply large parts of New Jersey and the city of Philadelphia. With the same river system supplying water to major population centers in three states, there has been inevitable competition over access to its water. A 1954 Supreme Court decree established a basis for water sharing between New York, Pennsylvania, New Jersey, and Delaware.^26^ Then, in the early 1960s, the four states and the federal government signed a compact that resulted in the formation of the Delaware River Basin Commission (DRBC). The commission, which supports a full‐time staff, oversees water supply, pollution control, flood protection, watershed management, recreation, and other functions.^27^ One key task is permitting and regulating water use. The commission permits all major water withdrawals with knowledge of the sum water demands within the system and ensuring demand can be met even during dry conditions.

The Susquehanna River Basin extends through New York, Pennsylvania, and Maryland, where it feeds into the Chesapeake Bay, which is the nation's largest estuary. In the late 1960s, concerned citizens pushed for a compact commission modeled after the one established on the Delaware River. Thus, in 1970, a compact among the three states and the federal government resulted in the Susquehanna River Basin Commission (SRBC), which is responsible for water supply allocation, management of water resources, and comprehensive planning throughout the Susquehanna River Basin.^28^ New York has also signed the interstate Chesapeake Bay Watershed Agreement of 2014, which commits the state to participate in the Chesapeake Bay Program and work toward a set of shared goals for improving the quality of the water that ultimately flows into the Chesapeake Bay.^29^

##### 2.1.4.3 Barge Canal System

The completion of the Erie Canal in the 1820s and its subsequent expansion around the turn of the 20th century dramatically changed the hydrography of much of the state.^30^ The canal led to the formation of vast new reservoirs, such as Delta Lake and Hinckley Reservoir, to serve the canal as well as other water demands. The canal created new interconnections; in Western New York, numerous streams receive water diverted from the canal to support irrigation, hydropower, and recreational fishing. The canal also transformed the Seneca and Mohawk Rivers, manipulating their water levels with a series of dams and gates. In particular, outflow from the largest Finger Lakes (Keuka, Seneca, and Cayuga) to Lake Ontario is regulated by dams and locks on the Seneca River. The construction of the Erie Canal also coincided with the forced removal of the Haudenosaunee from their lands and the loss of access to sacred waters.^31^, ^32^ Tribal Nations in the region still feel the legacy of trauma associated with these past water governance decisions.

The canal—now operated by the New York State Canal Corporation, a subsidiary of the New York Power Authority—was managed for most of its history with the intent to support commercial barge traffic. As barge traffic has diminished, the Canal Corporation has broadened its operational goals to include minimizing flooding, diverting water to address drought, and enhancing recreation.

##### 2.1.4.4 Hudson River–Black River Regulating District

More than 100 years ago, New York State established the Hudson River–Black River Regulating District (HRBRRD)^33^ to help address extreme flows on rivers in Northern New York. Central to the regulating district's mission was the construction of dams that resulted in large reservoirs: Stillwater Reservoir, the Fulton Chain of Lakes, and the Great Sacandaga Lake. In operation since 1930, the existing set of reservoirs has helped prevent the highest stages of flooding on portions of the Hudson River and the Black River.

The Conklingville Dam, which created the Great Sacandaga Lake, the largest reservoir in the state, is the most significant control structure regulated by the HRBRRD. The current regulation (operating) plan for the Conklingville Dam is based on an agreement reached in 2002 between HRBRRD and various federal and state agencies, municipalities, and stakeholder groups. The regulation plan governs how much water is to be released each day for all combinations of reservoir elevation and downstream flows. The plan aims to manage the reservoir in such a way as to (1) maintain the lake at targeted elevations during the late winter to store water for flow augmentation; (2) maintain flows in the Hudson and Sacandaga rivers for water quality and fish habitats; (3) target lake elevations that enhance fall recreation; (4) minimize energy losses to affected hydroelectric projects through an “aggressive use of storage”; and (5) release stored water to enhance Sacandaga River whitewater recreation.^34^

### 2.2 Key climate hazards

Chapter 2 of this assessment, New York State's Changing Climate, provides an in‐depth look at projected changes in primary climate variables through the end of the 21st century. Changes in temperature, precipitation, and sea level all have direct effects on water resources, as Sections 3 and 4 of this chapter explain in more detail. The following are examples of such impacts:

- Higher temperatures and drought increase demand for water, particularly for irrigation.
- Precipitation affects lake water levels, streamflow, and groundwater recharge. Projected increases in total annual precipitation could increase average streamflow, but longer stretches of drought and more frequent droughts—especially those projected for summer months—could cause shorter‐term decreases.
- Heavy storms can overwhelm stormwater collection systems and cause CSOs that compromise water quality.
- Higher temperatures and more runoff can degrade water quality by promoting harmful algal blooms (HABs).
- Sea level rise raises the risk of coastal flooding, particularly when combined with intense storms that create storm surge. Such flooding can damage critical water and wastewater management infrastructure.
- Sea level rise brings salt water farther upstream and into the coastal water table, which could compromise surface or underground sources of drinking water.
- Extremely hot temperatures can cause operational challenges for wastewater treatment facilities, as can sea level rise for facilities that discharge to tidal waters.
- Power outages caused by extreme weather events can disable well pumps and disrupt water and wastewater treatment.

### 2.3 Nonclimate factors

Several nonclimate factors influence the quantity and quality of New York State's water resources, as well as the ability of public water supply systems to reliably meet water needs. These factors include land‐use patterns, aging infrastructure for water supply and wastewater conveyance, and changes in water demand. Climate hazards will interact with these nonclimate factors to create compounding impacts on the state's water resources, infrastructure, demand, and governance.

#### 2.3.1 Land use

The composition of land uses within New York State's 17 major watersheds influences the quality and quantity of water resources. Land uses that more closely reflect predevelopment conditions preserve the landscape's natural functions that promote infiltration and decrease the velocity of runoff. In addition to altering hydrologic function, land uses affect the quantity and types of pollutants transported via runoff to surface water and infiltrated to groundwater. In particular, urbanizing watersheds with increased amounts of impervious surfaces (e.g., paved roads, sidewalks, parking lots, rooftops) experience decreases in stream integrity. One modeling study that sought to predict stream quality based on the percentage of impervious cover found that watersheds with 25−30% impervious cover consistently exhibit signs of poor condition, including poor water quality and diminished aquatic diversity.^35^ The Ecosystems chapter also discusses land use as a nonclimate factor influencing water resources from an aquatic habitat perspective.

#### 2.3.2 Aging infrastructure

Water resources cannot be separated from water infrastructure and the entities that oversee the operation and management of this infrastructure. Unlike power or telecommunication utilities, which are typically centralized and cover an extensive geographic region, water infrastructure is operated and maintained by a mix of municipalities, water utilities, private water companies, and small community water suppliers. A key concern is whether entities responsible for water systems (whether private, public, or individual homeowners) have the resources and knowledge to monitor, manage, and upgrade infrastructure to adapt to climate change and address compounding stressors such as system aging, evolving demand, or new contaminants. New York State has examined aging infrastructure related to water supply and wastewater conveyance. For drinking water infrastructure in the state, some treatment and distribution systems are more than 100 years old even though pipes have a lifespan of 50−70 years and core components at treatment plants have a lifespan of 20−40 years.^36^ Of the 35,000 miles of sewer lines throughout the state, more than 40% are over 60 years old, and approximately 10% were installed prior to 1925.^12^ Aging infrastructure is susceptible to structural failures. NYSDEC estimates that 65% of the separate sanitary sewers over 60 years old experience sanitary sewer overflows due to factors such as heavy rains, snowmelt, insufficient system capacity, and broken sewer pipes.

#### 2.3.3 Changing water demand

Climate change can influence water demand, but so can other factors such as population change, human behavior, and technologies. New York's per capita residential water use has declined in recent decades with the adoption of more water‐efficient devices in homes. However, New Yorkers still use substantially more water in summer for activities such as watering lawns and gardens, filling pools, or washing cars. Demand can also be influenced by the prevalence of water‐intensive agricultural commodities and water‐intensive industry sectors.

### 2.4 Equity and climate justice

Water resources face multiple stressors due to climate change, and often these stressors compound water quantity and quality challenges that result from historical and systemic racism and injustice. Environmental and health hazards tend to have a disproportionate impact on vulnerable and historically marginalized individuals and groups. Factors that influence vulnerability include race, ethnicity, age, gender or sexual identity, income, ability status, national origin, and degree of English proficiency, as well as other intersecting identities. Researchers have linked drinking water disparities to the historical injustices of racial redlining,^37^, ^38^, ^39^, ^40^, ^41^ a practice that was widely applied throughout New York State.^42^, ^43^, ^44^, ^45^ In a 2019 report, Dig Deep and the U.S. Water Alliance identified race as the strongest predictor of individual access to water and sanitation in the United States, noting that Indigenous Peoples “are more likely to face water access issues than any other group.”^46^ The U.S. Environmental Protection Agency's (EPA's) definition of environmental justice refers to ensuring that no group bears disproportionate impacts and providing equal access to the decision‐making processes and regulatory agency decisions that affect one's environment and health.^47^, ^48^, ^49^, ^50^

Low‐income families and other populations face compounding water injustices such as drinking water affordability and equity concerns. *Drinking water affordability* refers to the cost of both drinking water and wastewater treatment, which is billed based on water usage. Some water bills also include fees for stormwater‐related services. Gerlak et al. define *water equity* as “fairness in the water‐related decision‐making process,” which is characterized by “full access to information and the ability to participate” as well as the ability “to modify decisions and outcomes to address imbalances in power, access, and distributive fairness.”^41^ *Water justice* works across social, ecological, and political spheres to address water challenges through fairness, equity, and participation in water decision‐making.^51^, ^52^ With climate change compounding existing stressors on water quality, availability, and demand, more data collection is needed moving forward to document water affordability, shutoffs, and drinking water quality violations statewide.^41^, ^49^, ^53^

In the remainder of this chapter, the subsections on vulnerabilities apply an environmental justice lens to highlight communities, governments, groups, or regions that could be particularly vulnerable to certain impacts. Specific topics include water affordability, resource limitations of small (often rural) communities, climate change‐induced relocation, and potential exposure of people in low‐income communities to polluted waters through CSOs.

### 2.5 Indigenous communities

The territories of nine federally recognized or state‐recognized Indigenous Nations share boundaries with New York State, and other Indigenous Nations maintain ties to ancestral territories located within the state despite being forcibly relocated. The assessment introduction introduces these Nations and their lands, which are particularly rich in water resources. Open water and wetlands account for more than 26% of the land cover on Indigenous lands statewide—well above the statewide total of 11.6%.^54^ Water itself and aquatic ecosystems are highly valued in Indigenous cultures for both the sustenance they provide and their spiritual significance.^55^, ^56^ However, in some Tribal Nations, including the Onondaga and Mohawk Nations, watersheds have suffered severe ecological impacts from industry.

Tribal Nation governments and utilities operate and maintain their own water supply systems and oversee water delivery for public supply, industry, agriculture, and other uses on Tribal lands. Tribal Nation public water systems operate in compliance with the federal Safe Drinking Water Act (SDWA), EPA's National Primary Drinking Water Regulations, and in some cases with other Tribally established standards and requirements. Two Tribal Nations in New York, the Saint Regis Mohawk Tribe and Seneca Nation of Indians, have received “Treatment in the Same Manner as a State” designation under the Clean Water Act for establishing and administering water quality standards programs. The Saint Regis Mohawk Tribe established an approved water quality standards program in 2007 and is responsible for managing ambient water quality, nonpoint source pollutants, and direct discharges to the waters of Akwesasne.^57^ The Saint Regis Mohawk Tribe water quality standards are higher than the minimum federal standards and represent the Tribe's commitment to water resource protection.

Tribal Nations further create water laws and work with federal and state governments to implement shared water resource management. Tribal water rights are protected under domestic laws and international conventions such as the United Nations’ Declaration on the Rights of Indigenous Peoples.^58^

Tribal Nations and Indigenous Peoples have multifaceted concerns about the potential impacts of climate change on water resources.^59^ Potential impacts are tied not only to drinking water, but also to other exposures and ceremonial practices.^59^, ^60^ Climate change threatens their cultural and spiritual connections to water, a topic also discussed in the Human Health and Safety and Ecosystems chapters. Research suggests that the severity of water resource impacts due to climate change will likely be greater for Tribal Nations and Indigenous Peoples.^61^, ^62^, ^63^ However, to date, there has been limited inclusion of Indigenous communities in the consideration of climate change impacts in New York. This chapter will provide some context for the unique challenges Tribal Nations and Indigenous Peoples face, such as the challenges involved in financing water infrastructure because of the lack of eligibility for state resources. It will also consider the distinct cultural connection many communities have to their local waters.

### 2.6 Opportunities for positive change

The construction, operation, and maintenance of natural and built water resources infrastructure is an evolving process. The aging of system components, evolving and expanding regulatory requirements, changes in demand, and introduction of new technology already necessitate continual repair and replacement of built infrastructure. Climate change underscores the importance of incorporating resilience, redundancy, and flexibility into infrastructure and system operation. Especially given the long‐term underinvestment in water infrastructure,^64^ efforts and resources to address climate change can serve as an impetus to address other longstanding problems. This can include updating deteriorating or underperforming source water, water and wastewater treatment, distribution, and conveyance system infrastructure; improving infrastructure in areas prone to flooding; installing new equipment that reduces operational costs; implementing and improving asset management programs; enhancing water and energy conservation measures; and building new relationships among public water systems and across watersheds. Section 6.1 looks at some of these opportunities in greater detail.

## 3 WATER RESOURCES IMPACTS AND VULNERABILITIES

This section presents a summary of climate impacts to surface water quantity and quality and a separate summary of impacts to groundwater quantity and quality. The discussion of adaptations for addressing these impacts and vulnerabilities in Section 5 takes a more holistic approach, acknowledging that many adaptations are simultaneously beneficial for both surface water and groundwater, given their connections.

An understanding of possible changes in water availability, not just changes in precipitation and temperature, is especially important for the discussion in this section. Water availability refers to precipitation that has not been lost as evaporation and evapotranspiration (i.e., surface drying or use by plants) and remains available for use. Available water is measured as the flow of water in streams, the stored surface water in lakes and reservoirs, and the stored groundwater in aquifers. However, the amount of water available in streams, lakes, and groundwater does not change in exact proportion to precipitation and temperature. Several processes determine how precipitation and temperature influence available water. For instance, precipitation falling as snow is stored in snowpack until it melts, delaying changes to flow in streams. Alternatively, precipitation falling in intense storm events may disproportionately generate runoff that immediately enters streams or rivers, instead of groundwater recharge. Additionally, differences in land cover create variations in the landscape's ability to efficiently absorb and store precipitation no matter the intensity. During spring and summer when plants are most active, evapotranspiration depletes stored water, and light spring and summer rainfall have less effect on streamflow than during cooler, wetter months when the ground remains more saturated. Estimates of future changes in available water can be inferred in two ways: (1) through trends in historical records of streamflow, groundwater levels, or lake levels; and (2) through hydrologic modeling studies that attempt to simulate how climate projections of future precipitation and temperature interact to impact water availability.

### 3.1 Changes in water demand

A changing climate could lead to changes in water demand throughout the state. Residential water per capita use has generally declined in recent decades as more water‐efficient appliances and fixtures have been adopted. However, at the residential scale, water use often increases in the summer as homeowners use more water for watering lawns and gardens, filling pools, or washing cars. Longer summer‐like conditions in a changing climate could, therefore, lead to demand increases. A study of water use in Ipswich, Massachusetts, in 2005 noted that the town applied a 50% rate premium in the summer to better manage water use.^65^ This same study found that lawn size—when also controlling for house size—was an important predictor of homeowner water use.^65^ A study in Canada^66^ found an approximately 10−45% increase in water demand during the summer months in humid cities. More locally, the city of Oswego issued a request for residents to reduce water use during the drought of August 2016. Although the city's water source (Lake Ontario) was not constrained by the drought, residents were using water at a rate greater than the capacity of the treatment plant.^67^

Agricultural water use is particularly sensitive to drought. In normal climate years, natural precipitation in New York State can provide nearly all the water crops need. During drought years, however, some farmers use stream water or well water for irrigation to maintain yields and product quality. This use of irrigation is especially important for high‐value crops such as apples, berries, grapes, and vegetables. An analysis of well installation records from four counties in Western New York over the last 20 years indicates a spike in wells installed for irrigation purposes during the drought of 2016 (Figure 10-5). Modeling of water use during the 2016 drought event indicated that irrigation water use could increase three to eight times during very dry years.^68^ Thus, in areas where many farms grow high‐value crops, competition for limited water resources could deplete stream water or groundwater resources over time. A lengthening growing season could also lead to changes in water demand for agriculture as well as recreation, an observation already made on Long Island.^69^ Refer to the Agriculture chapter for more information on changes in agricultural water demand.

**FIGURE 10-5:**
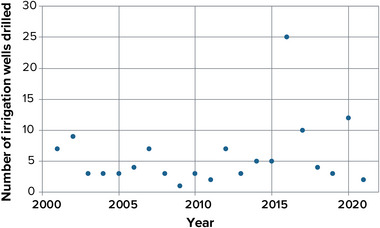
New irrigation wells drilled per year in agriculturally intensive counties in Western New York, 2001–2021. Specific counties are Genesee, Monroe, Orleans, and Wyoming. Well drilling peaked in 2016 during one of the most severe droughts in the last several decades, indicating that farmers shifted from depleted surface water supplies to groundwater. Data from NYSDEC (2023).^70^ Additional data obtained through a Freedom of Information Law request.

Water demand could also change due to shifting populations. While recent studies have examined the potential for the Great Lakes region to become a climate refuge, in part due to its cooler temperatures and access to fresh water, evidence of climate migration in the state remains anecdotal.^70^, ^71^ The Society and Economy chapter discusses migration in additional detail.

### 3.2 Surface water quantity and quality

Surface water quantity and quality is important for a variety of uses including drinking water, irrigation, recreation, and habitat. Climate change could affect surface water quantity and quality in several ways.

#### 3.2.1 Surface water quantity

Surface water quantity focuses on the amount of water in lakes, rivers, and streams to meet various needs. This section examines historical trends and variability of water availability in New York State and the projected changes to water availability in a changing climate. This section then addresses climate impacts and vulnerability considerations for different types of public water systems that rely on surface water sources.

##### 3.2.1.1 Historical records, trends, and variability of water availability

In the historical records, the four major river systems in New York (as determined by drainage area) do not exhibit any recent shift in flow outside of the range in variation historically seen in the record (Figure 10-6). Although there is evidence of regional warming in the last several decades,^72^ as of yet, there is no strong evidence of changes in annual average flows on major rivers. Despite variations in flow over time that often last multiple years, the long‐term mean has remained relatively stable. Broader analyses of trends in flow demonstrate upward shifts in mean average discharge in New England and parts of the Northeast^73^, ^74^ and within New York State.^75^, ^76^ This general increase in wetter conditions is sometimes deemed a pluvial—a climatological period of unusual wetness. The initiation of this pluvial is evident across much of the northeastern United States starting in the early 1970s,^77^ with an additional upward shift in precipitation on the eastern side of the state beginning in the early 2000s.^78^

**FIGURE 10-6:**
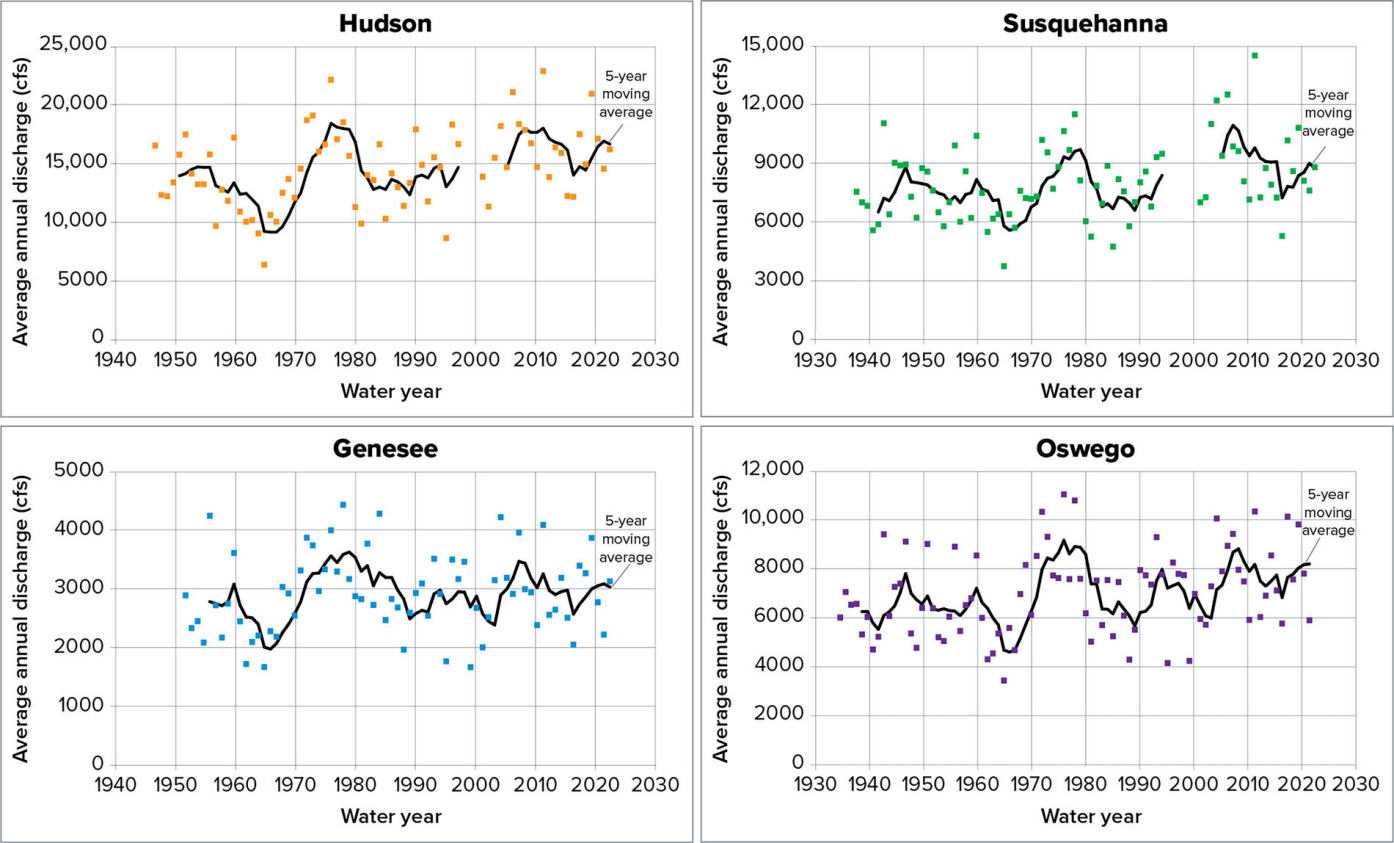
Trends in annual average discharge on major river systems in New York State. The time periods of data presented for each river are as follows: Hudson, 1947–2022, Susquehanna, 1938–2022, Genesee, 1952–2022, and Oswego, 1935–2021. Points indicate discharge as measured at United States Geological Survey gages averaged over the water year, in cubic feet per second (cfs). The solid black lines are 5‐year moving averages. Hudson River discharge is measured at Green Island Dam in Troy, Susquehanna River discharge is measured at Waverly, Genesee River discharge is measured at the Ford Street Bridge in Rochester, and Oswego River discharge is measured at Lock 7 in Oswego. Data from USGS (2022).^81^

Despite relative stability in long‐term mean annual flows, there have been observations of seasonal changes in streamflow in New York.^75^, ^79^, ^80^ In particular, there have been observations of higher streamflows occurring earlier in the spring, presumably as less precipitation falls as snow or as snow melts earlier.^80^

The drought of record, or the driest period in the observed streamflow record, serves as an indicator of the driest possible conditions likely to occur. Therefore, the drought of record is often used to plan and design water resources infrastructure. Figure 10-6 shows a consistent low point in flow in the early 1960s across all four river systems profiled. From 1961 to 1965, drought conditions were most severe in Southeastern New York, but it was generally abnormally dry across much of the state. For many locations in the state, this period is identified as the drought of record, and its streamflows are used as a worst‐case planning scenario. Supplemental data from tree ring observations in very old trees suggest that the 1960s drought was one of the most severe droughts in the state in at least the last 500 years, if not longer.^77^, ^78^ Given that the 1960s drought was severe by multicentury standards, it should be a reasonable proxy for future severe dry conditions.^77^

##### 3.2.1.2 Projecting climate change impacts on water availability

Climate simulations can provide insights into future water availability. Modeling of future hydrology in the Northeast typically identifies seasonal shifts in the timing of river and stream discharge, consistent with existing observed seasonal shifts. While changes in long‐term mean regional precipitation patterns are difficult to predict,^82^, ^83^ most models predict that discharge will increase in winter^84^, ^85^ as less precipitation falls as snow and as warmer temperatures increase precipitation. Figure 10-7 shows a representative example of average changes in river flow by month with increases anticipated in January and February and decreases much of the rest of the year, with the largest deficit occurring in late fall. Some of the most extensive modeling of future hydrology in New York State focuses on the Catskills region, which supplies water to New York City. These studies indicate a likely increase in annual discharge as well as a shift in the timing of peak flows from spring to winter.^86^, ^87^ An assessment of differences between historical cold and warm winters corroborates the modeled findings.^88^

**FIGURE 10-7:**
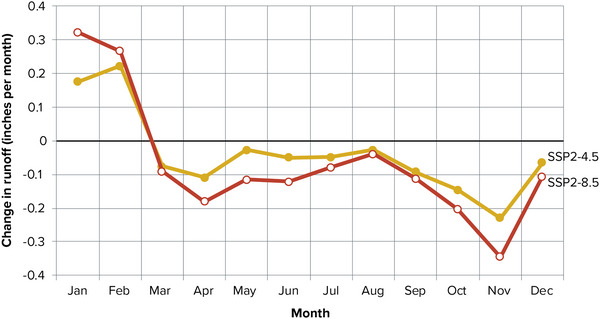
Modeled changes in mean monthly runoff on the Susquehanna River between 1980–2010 and 2050–2074. The USGS National Climate Change Viewer projected the changes using the ensemble mean of 20 global climate models. Changes in discharge based on the climate change viewer are similar for other river systems in New York State. The red line is the very high greenhouse gas emissions scenario used in this assessment: Shared Socioeconomic Pathway (SSP) 5‐8.5. The yellow line is the intermediate emissions scenario (SSP2‐4.5). Data from the USGS National Climate Change Viewer.^89^

Modeling studies are of particular value because they can assess concurrent changes in precipitation and evaporation. One study used modeling that replicated the general weather patterns associated with the 1960s drought in the Northeast but updated the thermodynamic characteristics of the atmosphere to represent the likely future conditions of the mid and late 21st century.^90^ It found that precipitation increased in some years during the drought, but that overall water availability decreased due to increased evaporative loss. Importantly, the study also found that drought onset came more quickly with less warning as enhanced evaporative demand exacerbated precipitation deficits.^90^

Despite the extensive use of models, simulating how a changing climate may modify hydrology remains a challenge. For instance, despite the importance of the Great Lakes, only in the last several years have researchers applied predictive models that couple the interaction between the atmosphere and water surface to the Great Lakes.^91^ Lake ice is one variable that has proven difficult to project with confidence. The presence of lake ice diminishes evaporation, which influences lake levels and the weather patterns that develop over the lake (i.e., lake‐effect snow and rain). New York State's Changing Climate discusses observations, projections, and uncertainties for lake ice and other physical attributes of the Great Lakes in detail.

Modeled estimates of future evapotranspiration can also be uncertain. There are no standardized techniques for modeling future changes in evapotranspiration, and different modeling studies for the same region can result in differing outcomes of projected future streamflow.^92^, ^93^ In particular, many hydrologic modeling studies are done “offline” from climate models, simply using climate model output but not allowing for any feedback. One study found that offline models often do not match evapotranspiration trends as predicted by the climate models themselves, in part because of a lack of feedback between variables such as soil moisture and air temperature, as well as a lack of consideration of how the change in carbon dioxide concentration affects the function of stomata (the small openings in leaves that allow carbon dioxide in and water vapor out) in plants.^94^

##### 3.2.1.3 Climate impacts on surface water quantity for public water systems

From a human perspective, surface water quantity is critical for potable water use. From a habitat perspective, surface water quantity is essential to sustaining aquatic life. Given the Ecosystems chapter's focus on surface water resources from the perspective of sustaining habitats, this section focuses on impacts to the quantity of surface water supply for potable water. This section divides surface water sources into several broad categories based on features that influence their vulnerability to climate change.

###### 3.2.1.3.1 Run‐of‐river systems

In run‐of‐river systems, water is drawn directly from a river or stream with no additional storage provided by a reservoir or lake. Run‐of‐river systems depend on the water immediately available in the river. Typically, run‐of‐river systems are found on large rivers where withdrawn potable river water is a small fraction of the total river flow. For instance, the city of Binghamton treatment facility withdraws an average of 4.7 million gallons per day,^95^ while the lowest observed flow on the Susquehanna River (the source) was 54.9 million gallons per day based on 110 years of records.^96^ However, because there is no storage to buffer short dry periods, run‐of‐river systems could be sensitive to any changes in the magnitude of short‐term droughts. While projections indicate New York State will generally have more rainfall in the future, this does not prevent periods of below average rainfall.^72^ With increased evaporation likely in the future due to warmer temperatures^72^ and greater vapor pressure deficits, even if rainfall deficits are similar to those in the past,^97^ actual streamflow would decline more than it did in past droughts.

Examples of run‐of‐river water systems include those serving the city of Binghamton and surrounding areas (44,564 people),^95^ the city of Elmira (approximately 54,000 people),^98^ Cornell University (more than 35,000 people),^99^, ^100^ the city of Olean (nearly 14,000 people),^101^ the city of Cohoes (nearly 17,000 people),^102^ and the city of Watertown (approximately 45,000 people).^103^ Some of these municipalities have supplemental wells or interconnections, but all rely predominantly on river water. Although system‐specific analyses would more accurately assess vulnerability, systems in which demand approaches minimum flow have the most cause for concern in a changing climate.

Climate change could also affect the quality of potable surface water supplies from the Hudson River. Several communities obtain water from the mid‐Hudson River, including more than 100,000 people in seven communities served by the Hudson River Drinking Water Intermunicipal Council (known as the Hudson 7): the town of Esopus, town of Hyde Park, town of Lloyd, town and city of Poughkeepsie, and town and village of Rhinebeck.^104^ The Hudson River up to the Green Island Dam in Troy is technically an estuary because it involves the mixing of fresh water and salt water. The salt front is defined as the point in the estuarial water body where the chloride concentration exceeds 100 parts per million. The position of the salt front changes depending on the amount of freshwater moving downriver.^105^ The salt front can extend up to Newburgh by the fall during a normal year. However, when drought periods diminish freshwater inflows into the Hudson River, the salt front can extend farther; this occurred during the drought periods in 2002 and 2007 when the salt front extended up to Poughkeepsie.^106^, ^107^ Thus, even though there may be no visible change in the quantity of water in the Hudson, the water could become too salty to meet drinking water standards during drought periods.

Even if future droughts are similar to those in the past, increases in sea level will change the elevation of the ocean outlet and lead the salt front to move further upriver. There are no known analyses that simulate the effects of increasing sea level on salt front for the Hudson River, although such analyses in the Chesapeake Bay^108^ and in Spain^109^ have shown upriver shifts in the salt front. Additionally, there is both anecdotal information and preliminary modeling^110^ that indicates that the salt front of the Delaware River, another regional estuary, could move several miles upriver due to sea level rise. The modeling on the Delaware River used 2002 drought period river flows and compared the salt front position with different amounts of sea level rise. With a half meter of sea level rise (the approximate amount predicted by mid‐century), the salt front is projected to advance upstream on the Delaware River by 3.7 miles relative to the baseline condition with no sea level rise.^111^

###### 3.2.1.3.2 Great Lakes systems

The Great Lakes hold about 21% of global fresh water^112^ that is not bound in ice or snow. Great Lakes water levels have fluctuated over their recorded history, but only over several feet.^113^ There is little chance that the Great Lakes would be depleted to an extent that would threaten their use as a potable water source, and new protections in place via the Great Lakes Compact prevent water transfers outside the Great Lakes Basin.^114^, ^115^ Therefore, the portions of the Great Lakes Basin that fall within New York State—Lake Erie, the Niagara River, Lake Ontario, and the St. Lawrence River—are reliable water sources from a surface water quantity perspective.

Major metropolitan areas in Western and Central New York rely predominantly on withdrawals from the Great Lakes Basin. This includes the metropolitan areas of Buffalo, Rochester, and Syracuse—with water authorities that collectively provide water to nearly 700,000 people^116^—plus the smaller cities of Oswego and Ogdensburg. The Buffalo area relies entirely on Great Lakes water. The Monroe County Water Authority (serving the suburbs and towns around Rochester) obtains all of its water from Lake Ontario and can provide supplemental water to the city of Rochester.^117^ The Onondaga County Water Authority (serving towns surrounding Syracuse) obtains about half of its water from Lake Ontario and the other half from Otisco Lake. The city of Syracuse maintains an interconnection with the Onondaga County Water Authority to obtain supplemental Lake Ontario water during droughts.^118^ Even when a public water system does not rely entirely on a Great Lakes source, during extended droughts, the system can increase the proportion of water drawn from a Great Lakes source to help conserve the more limited inland source as long as possible.

There are possible limitations in the Great Lakes Compact that do not fully protect the Great Lakes from future depletion. The Great Lakes Compact seeks to prevent withdrawing more water than is gained in a typical year. However, persistent drought in regions outside of the basin could put pressure on Great Lakes states to divert Great Lakes water. As currently written, the Great Lakes Compact does allow diversions outside the basin to communities in counties that straddle the basin, as long as member states unanimously agree and as long as there is a demonstration that it is the only viable water source. In 2018, the Great Lakes states permitted the city of Waukesha, Wisconsin, to withdraw Lake Michigan water to replace a water supply contaminated by radium.^119^ As a condition for the diversion, Waukesha will return flows of treated water back to Lake Michigan. This was the first test of the Great Lakes Compact's ability to minimize outside diversions. However, the pressure to acquiesce to diversions to neighboring communities could increase if those communities experience widespread and severe water stress.

###### 3.2.1.3.3 Reservoir systems

Multiple public water systems in New York use reservoirs to make up for seasonal differences between user demand and the natural supply immediately available in a stream or river. Most of the state's rivers and streams have much higher flows during winter and spring than in summer and fall. Reservoirs capture this winter and spring flow and save it for use during summer and fall. Unlike run‐of‐river systems, reservoir systems are not sensitive to day‐to‐day levels of flow in a river. Instead, reservoir systems are sensitive to multimonth dry periods that change the overall volume of water entering a reservoir and the demand for water from the reservoir. While reservoirs are in large part designed to address seasonal variations in available water, most reservoirs are also sized to account for drought periods.

The sensitivity of reservoir systems to drought duration and intensity is strongly dependent on the specific characteristics of each system. In the simplest terms, reservoir systems’ sensitivity to drought is the ratio between the maximum storage in the system and the daily demand. Systems with greater storage volume relative to demand will only face water shortages for relatively long (and thus less frequent) droughts. For instance, the city of Troy obtains its water from the Tomhannock Reservoir. The reservoir has a maximum storage volume of 12.3 billion gallons, while its average finished water production is 17.27 million gallons per day,^120^ giving this reservoir a ratio of storage to demand of roughly 700 to 1. In contrast, the city of Ithaca has an average daily demand of around 2.5 million gallons per day but has reservoir storage of around 110 million gallons.^121^ Thus, Ithaca has a ratio of storage to demand of approximately 40 to 1. During a severe drought in 2016, Ithaca announced it was potentially within 30 days of running out of water.^121^

In terms of sensitivity to climate change, reservoir systems with a lower storage‐to‐demand ratio could face water shortages for droughts within as little as a few months. Systems with greater ratios between storage and demand would only face shortages for droughts lasting a year or more. In general, most climate projections indicate that New York State will be wetter at an annual time scale, with a tendency for more precipitation and streamflow in spring and less in summer and fall. If historical variations in annual precipitation are coupled with a shift in timing and greater evaporative demand in summer, it is reasonable to expect more frequent short‐term droughts, particularly in late summer.^72^ Public water systems that rely on reservoirs with lower storage‐to‐demand ratios could be susceptible to increased frequency of water shortages in the future.

#### 3.2.2 Surface water quality

Increasing scientific evidence indicates that changing climate patterns are negatively affecting lakes, reservoirs, and rivers that provide drinking water. This section focuses primarily on changes to the water quality parameters of most concern in drinking water: turbidity, waterborne pathogens, presence of HABs, and disinfection byproducts (DBPs). It also considers the possible impacts of climate change on the mobilization of other contaminants of concern in drinking water.

NYSDEC assesses the surface water quality of each of the state's 17 watersheds (refer to Figure 10-1) once every 5 years.^122^ Through this watershed assessment, NYSDEC collects information regarding toxic impacts, habitat, and aquatic life conditions. NYSDEC compares the data with the state's water quality standards to determine which water bodies are supporting their best uses (also referred to as designated uses) and which water bodies are impaired. The most recent list of impaired water bodies for New York State was published in 2018. According to this list, the state has 835 instances in which a pollutant is causing a best use/designated use impairment.^123^ Pollutant sources listed for these impairments include urban/stormwater runoff, contaminated sediment, atmospheric deposition, on‐site wastewater treatment systems (septic systems), agriculture, and CSOs. Many of these pollutant sources are driven by or influenced by wet weather; therefore, increased total precipitation and heavier rainstorms could exacerbate these existing sources of impairment to surface water quality.

This section provides an overview of the pollutants of concern most relevant to potable water supplies in New York State, and how climate change will affect these pollutants over time. Additional discussion on impairments related to agricultural use is included in the Agriculture chapter, and the Ecosystems chapter discusses water quality in relation to aquatic life. Additional discussion on waterborne disease is included in the Human Health and Safety chapter.

##### 3.2.2.1 Turbidity

Turbidity is an indicator of water clarity. The higher the turbidity, the less clear the water. Turbidity is typically reported in nephelometric turbidity units (NTUs). This is a straightforward measurement of how much light is scattered when a beam of light travels through a water sample in a vial or cuvette. Increased turbidity most often occurs in lakes, streams, or rivers with the mobilization of fine sediments. In rivers and streams, large storm events that increase streamflows can erode channel banks, pick up loose soils from floodplains, or move bed material. In smaller watersheds or watersheds with large amounts of impervious surface, increased precipitation intensity during storm events will likely translate into higher streamflows that could increase erosion and turbidity.^124^ Biotic compounds such as decomposing organic matter and phytoplankton can also contribute to turbidity.

Turbidity is a water quality challenge for several reasons. Higher turbidity causes surface water temperatures to increase because the particles absorb sunlight. This warming in turn decreases dissolved oxygen levels needed to support aquatic life. Warmer water can also help fuel HABs (refer to Section 3.2.2.3). Turbidity also serves as an indicator for microbial contamination of drinking water both before and after treatment. Based on federal requirements, turbidity is monitored in all public water supplies using surface water. Public water suppliers with sources in which turbidity exceeds regulatory limits might need to upgrade their treatment processes or identify new water sources.

Despite extensive monitoring to meet regulatory requirements, there has been no systematic study of trends in turbidity across the state's drinking water systems in recent decades. The lack of an extensive study could mean that the vast majority of water suppliers have managed to adapt treatment processes or water sources (i.e., by shifting to an alternate source during a high‐turbidity event). Such adaptation does come with additional costs. A shift to new water sources requires new infrastructure, and filtration of higher turbidity water can require chemical additions, according to a case study in Texas.^125^ Dealing with higher turbidity can also lead to reduced filter rates because of the need for more frequent back‐washing and more solids handling.

The water systems in the state with a Filtration Avoidance Determination (FAD) have been evaluated more extensively for possible increases in turbidity. The NYSDOH can grant an FAD under SDWA (40 CFR Part 141)^126^ to water supplies with generally low turbidity and microbial concentrations in their source water. Avoiding filtration can offer substantial cost savings,^127^ but systems without filtration are particularly sensitive to possible changes in turbidity. In New York State, the two water supplies with FADs are New York City's water supply reservoirs located to the west of the Hudson River (referred to as the Catskill/Delaware supply reservoirs) and Skaneateles Lake that supplies the city of Syracuse.

In the New York City system, there has been extensive investigation of turbidity in the Ashokan Reservoir and Esopus Creek, the stream that feeds into the reservoir. Esopus Creek had five extreme streamflow events between 2010 and 2011 that exceeded the 10‐year recurrence interval streamflow (Q_10_), three of which occurred during the 21‐month period starting in January 2010.^128^ During this time, the Ashokan Reservoir experienced turbidity levels greater than 100 NTU,^129^ which is 20 times greater than the threshold for the entry point surface water turbidity for unfiltered water systems.^130^ Elevated turbidity does not mean that the water is entirely unusable but may require changes to operational practices. High turbidity sometimes requires the addition of coagulants (e.g., aluminum sulfate, polyaluminum chloride, ferric chloride) to help settle out particulates. During high‐turbidity events in the New York City system, aluminum sulfate (alum) is episodically added to the raw water from Ashokan Reservoir at the Pleasantville Alum Plant, located toward the end of the Catskill Aqueduct. The settled particles accumulate in the Catskill Influent Chamber Cove in Kensico Reservoir. Existing monitoring data for aquatic organisms in the cove does not indicate adverse impacts from the alum application,^131^, ^132^ although studies in other locations have noted impacts to fish and benthic macroinvertebrates in the year during and after alum treatment.^133^

Simulations using older emissions scenarios have predicted that the average wintertime streamflow of Esopus Creek will increase by 12% and 20% and reservoir turbidity will increase by 11% and 17% in 2046–2065 (with emission scenario B1) and 2081–2100 (with scenarios A1B, A2, and B1), respectively.^129^, ^134^ Ambient stream turbidity is expected to increase from November to March and decrease during April during the same future years^135^ in response to the predicted increase in winter rainfall, reduction in snowfall, and earlier snowmelt runoff. This, in turn, could increase the frequency of alum treatment needed to reduce turbidity. Frequent use of alum increases costs, and it also adds sediment to reservoir beds that could eventually require dredging. More frequent extreme precipitation events combined with changes in snowfall and snowmelt could introduce more uncertainty in the future prediction of turbidity in source water bodies around the state, including Ashokan Reservoir.

New York City has received an FAD for its source water from the Catskill and Delaware Watersheds since 1993^136^, ^137^ with periodic reviews by EPA. The current FAD is in effect from 2017 to 2027. While New York City has successfully maintained the FAD for the Catskill and Delaware watersheds for 30 years, source water turbidity has been one of the main challenges. The third watershed supplying water to New York City, the Croton watershed, experienced a notable deterioration in water quality in the 1990s. The Croton Water Filtration Plant came online in the Bronx in 2015 to meet the EPA requirement for filtration against waterborne pathogens and also to prepare for the effects of climate change.^138^, ^139^ The water quality decline in the Croton watershed was mainly attributed to the suburbanization of formerly rural watershed land at the time; however, climate‐induced increase in stream and reservoir water turbidity could negatively affect watersheds with FADs in the future.

Syracuse has drawn water from Skaneateles Lake since 1894 with no filtration.^140^ The most recent FAD granted to the city in 2004 has no explicit expiration or renewal date; as long as the water quality remains high, the city is not required to conduct filtration.^141^ However, the lake's turbidity has increasingly fluctuated since 2009. The occurrences of high‐turbidity events have increased since 2006; these events are attributed to high wind (not heavy precipitation) and could lead to the closure of one of the two intakes.^141^ The future of the FAD for Skaneateles Lake is especially challenging to predict due to turbidity–HAB interaction. Increased sediment and nutrient loading from more extreme weather can exacerbate this interaction at a decadal or longer timescale, but the underlying biogeochemical processes are distinct at shorter temporal scales. HABs are rarely observed during a storm‐driven high‐turbidity event but often develop during calm and sunny days afterward as light availability in the water column and surface water temperature increase.

Another possible source of turbidity changes may come from unwanted erosion in streams and rivers. Streams and rivers have undergone extensive channelization, impoundment, and realignment due to industrialization and urbanization, which has changed the streams’ natural, dynamic exchange of sediment.^142^ With these disturbances, streams and rivers have experienced a loss of sediments from certain reaches and more deposition in other places, such as behind dams. Increases in high‐flow events with a changing climate—especially in small or urban watersheds—can remobilize these legacy sediments, leading to unwanted blockages and hydraulic disturbances in streams, as well as the movement of pollutants adsorbed on these sediments.

While turbidity is a useful proxy for microbial contamination in pre‐ and post‐treatment water, its utility is low in studies of ambient lake and reservoir water quality where microbes are expected and can be harmlessly ubiquitous. Water transparency estimated by a Secchi disk and human vision indicates the degree of vertical light attenuation along the water column, but this includes light absorbed by biotic compounds such as dissolved organic matter and photosynthetic pigments. While some stream monitoring programs involve quantification and/or characterization of (re)suspended fine sediment particles that directly contribute to turbidity, they are beyond the scope of most lake and reservoir water quality monitoring programs in the state, including the Citizen Science Lake Assessment Program (CSLAP). A more systematic investigation of light‐attenuating entities in water could help researchers more fully understand the interactions between climate change and sources of water turbidity.

##### 3.2.2.2 Pathogens

Surface water and groundwater can contain a wide variety of bacteria, viruses, and parasites, some of which can cause human illnesses and, therefore, are called pathogens. Coliform bacteria, such as fecal coliform, are indicator organisms that signify the presence of pathogens in water. While some pathogens occur naturally, most common waterborne pathogens originate from land‐based fecal sources such as agricultural runoff, failing septic systems, urban stormwater runoff, and CSOs. Increases in total precipitation and heavy storms can drive increases in runoff that carries pathogen loads into water bodies.

Standard drinking water treatment steps such as filtration and disinfection (e.g., chlorination, ozonation, ultraviolet radiation) are effective in eliminating pathogens in finished water, but these treatments can become less effective when the systems are overwhelmed with highly turbid raw water, as the suspended particles interfere with the physical and chemical processes that remove and kill pathogens.^143^ As discussed in Section 3.2.2.1, the changing climate can cause more extreme rainfall events and fluctuations in streamflows, leading to increased turbidity in raw water.

While the epidemiological relationship between drinking water turbidity and occurrences of gastrointestinal illnesses appear dependent on study methodologies and local and seasonal contexts,^144^, ^145^ a few studies show correlations between the two. One in New York City found a small but statistically significant positive association between the post‐treatment city water turbidity (which reflected fluctuations in pretreatment source water turbidity) and emergency room visits due to acute gastrointestinal illnesses in spring, especially for 0‐ to 4‐year‐olds^146^ (note: this study was based on data from 2002 to 2009, before New York City's Catskill/Delaware Ultraviolet Treatment Facility began operation in 2013).^147^ Another study in Philadelphia, Pennsylvania, focused on residents aged 65 years or older and reported statistically significant associations between drinking water turbidity (from filtered municipal systems operating within EPA standards) and gastrointestinal‐related hospital admissions.^148^

##### 3.2.2.3 Harmful algal blooms

Warm, nutrient‐rich surface water can promote the overgrowth of cyanobacteria and algae, impairing water quality best uses associated with recreation, drinking water, and aquatic life. Some cyanobacteria and algal blooms are labeled as HABs because they involve species that produce toxic compounds. Research has tied climate change to an observed global increase in summertime HABs.^149^, ^150^ Cyanobacteria generally coexist with microscopic algae in cooler and oligotrophic (i.e., nutrient‐poor) waters, but cyanobacteria can outcompete microscopic algae in warm, eutrophic (i.e., nutrient‐rich) waters. Climate change not only warms the air and water temperatures in general but also increases the stability of the warm surface water layer in temperate lakes and reservoirs, which favors the growth of cyanobacteria against microscopic algae. Simultaneously, this phenomenon can alter the overall thermal structure of the water column and decrease dissolved oxygen toward the bottoms of lakes, which can lead to enhanced nutrient release from sediments.^151^ Additionally, increases in high‐intensity precipitation due to climate change can result in runoff events that provide pulses of nutrients.^152^ Some research suggests that oligotrophic lakes (those with normally low nutrient levels) can be particularly susceptible to increases in cyanobacteria from more frequent nutrient input events.^153^ The Ecosystems chapter provides additional discussion about the observed and projected occurrence of HABs in New York's waters, including the Great Lakes.

HABs occurred in more lakes and reservoirs across New York in 2016–2019 compared with 2012–2014.^154^ As it is unlikely that drastic changes in watershed land use and stream/river water quality occurred during the 8‐year timespan to drive a statewide nutrient loading increase, it is likely that changing precipitation and temperature patterns contributed to the increased occurrence of HABs in lakes and reservoirs. Skaneateles Lake was one such water body that formally documented HABs for the first time in September and October 2017,^155^ despite being classified as oligotrophic due to low total phosphorus, low chlorophyll *a* concentrations, and high water transparency (i.e., low turbidity). The Skaneateles Lake HABs in 2017, 2018, and 2019 contained cyanotoxins at levels that can harm human and animal health.^154^ While eutrophication is often suggested as a major cause of cyanobacterial HABs, a 2019 study of Skaneateles Lake water quality data showed a significant positive correlation only between annual precipitation and surface chlorophyll *a* concentration; there was no significant correlation between total phosphorus and chlorophyll *a* concentration.^156^ This result suggests that weather‐driven factors other than external loading of total phosphorus or sediment need to be investigated further as potential HAB contributors.

Owasco Lake, a mesotrophic (moderately productive) source water lake directly west of Skaneateles Lake, also experienced HABs in the 2016–2019 period. In 2016, low concentrations of a class of cyanotoxins called microcystins were detected in the finished drinking water sourced from Owasco Lake.^157^ In response, the city of Auburn and town of Owasco, two municipalities using Owasco Lake as their municipal water source for a total of approximately 60,000 people, retrofitted their water treatment plants with granulated activated carbon systems to remove cyanotoxins.^157^ The town of Owasco has since upgraded its granulated activated carbon system to a permanent one,^158^ while the Cayuga County Water and Sewer Authority sought alternative water sources to supplement Owasco Lake.^157^

Besides cyanotoxins, some organic matter associated with the cyanobacterial biomass can react with chlorine and form toxic DBPs, which are discussed more broadly in Section 3.2.2.4.

Additionally, certain filamentous cyanobacteria have also been identified as producers of geosmin and 2‐methylisoborneol,^159^ both of which are not known to be harmful to humans but give a musty flavor to finished drinking water, which often leads to high consumer dissatisfaction and public relations issues.

Climate change has reduced the duration of ice cover for lakes and reservoirs.^160^, ^161^, ^162^, ^163^ Lake ice‐out (thaw) dates have been identified as a major driver of lake productivity and cyanobacterial abundance in lakes in the northeastern United States.^164^ In nutrient‐poor lakes in Sweden, different types of cyanobacteria (e.g., toxin producers vs. nonproducers) responded differently to (1) water temperature increase, (2) recovery from acidification, (3) reduced nitrogen deposition, and (4) browning (increase in colored dissolved organic matter).^165^ These four stressors are relevant to water bodies in New York State, and prediction of future HABs will require careful evaluation of these and other local stressors. In general, models have predicted that nutrient‐poor northern temperate lakes (based on observed data from New Hampshire and Wisconsin) could be more sensitive to climate warming than eutrophic lakes, and that climate warming could amplify the effects of eutrophication.^166^

HABs can lead to human health impacts through exposure beyond drinking water or recreational activities. In 2021, EPA hosted a series of webinars on managing HABs in Tribal waters. Researchers working with California Tribes identified a conceptual pathway for HAB impacts related to Tribal cultural uses (Figure 10-8). As adapted for Tribal Nations in New York State, exposure pathways impacted by HABs include ingestion of water through the collection and consumption of aquatic plants and animals; intergenerational water knowledge transmission activities; and ceremonial and spiritual practices. In many cases, these cultural uses of water are long‐term and repetitive in nature, suggesting multiple opportunities for exposure.^60^

**FIGURE 10-8:**
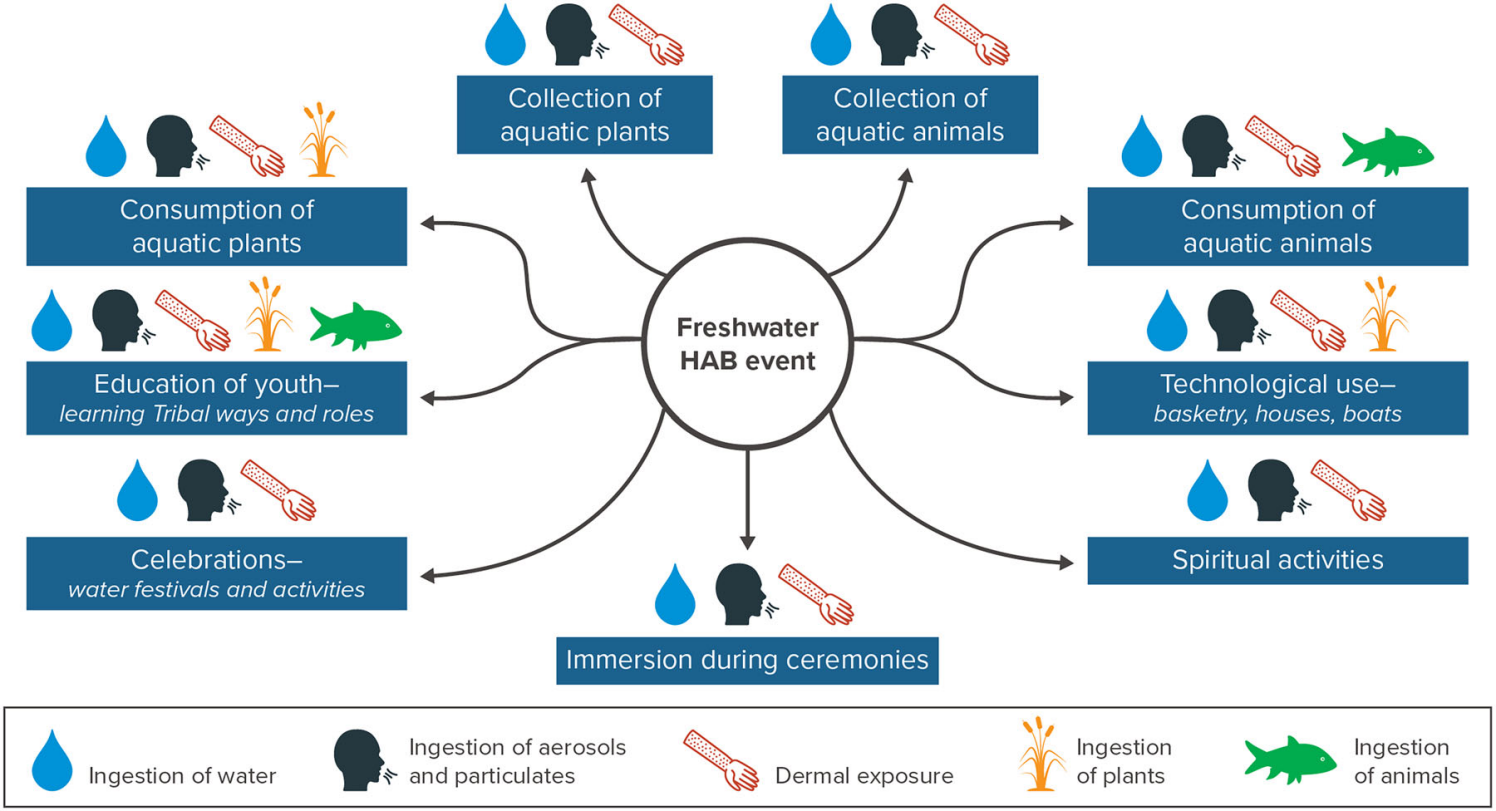
Tribal cultural use of fresh water and potential pathways for exposure to HABs. Adapted from an image by the Big Valley Band of Pomo Indians and the Karuk Tribe (with assistance from Meyo Marrufo and Dr. Jeanine Pfeiffer).^60^

##### 3.2.2.4 Disinfection byproducts

DBPs are chemical compounds that form via chemical reactions between natural organic matter in raw water and chlorine used in the disinfection process. Like turbidity, the concentration of natural organic matter in water can fluctuate due to seasonal changes and weather events. Climate change alters weather patterns and can also affect vegetation types in the watershed as air and soil temperatures and moisture availabilities dictate which plant species thrive. Different types of vegetation (e.g., upland conifers vs. wetland shrubs) result in different patterns of soil nutrient cycling, and the dead biomass has different chemical compositions and decomposition rates. This can change the types and amounts of organic matter that enters the runoff. Some DBPs can harm human health, and municipal drinking water systems are mandated to test their finished drinking water for certain contaminants, including DBPs, and publicize the results to the end users.

Drinking water quality in the state's public water systems is mostly acceptable in terms of chemical contaminants, with only sporadic exceedances of the maximum contaminant level (MCL) for the main parameters used to quantify DBPs: haloacetic acids (HAA5), and total trihalomethanes (total THMs or TTHMs).^167^ HAA5 and TTHMs are carcinogenic DBPs. Figure 10-9 depicts the New York population served each year by water systems with mean annual total TTHM concentrations greater than the MCL of 80 micrograms per liter.

**FIGURE 10-9:**
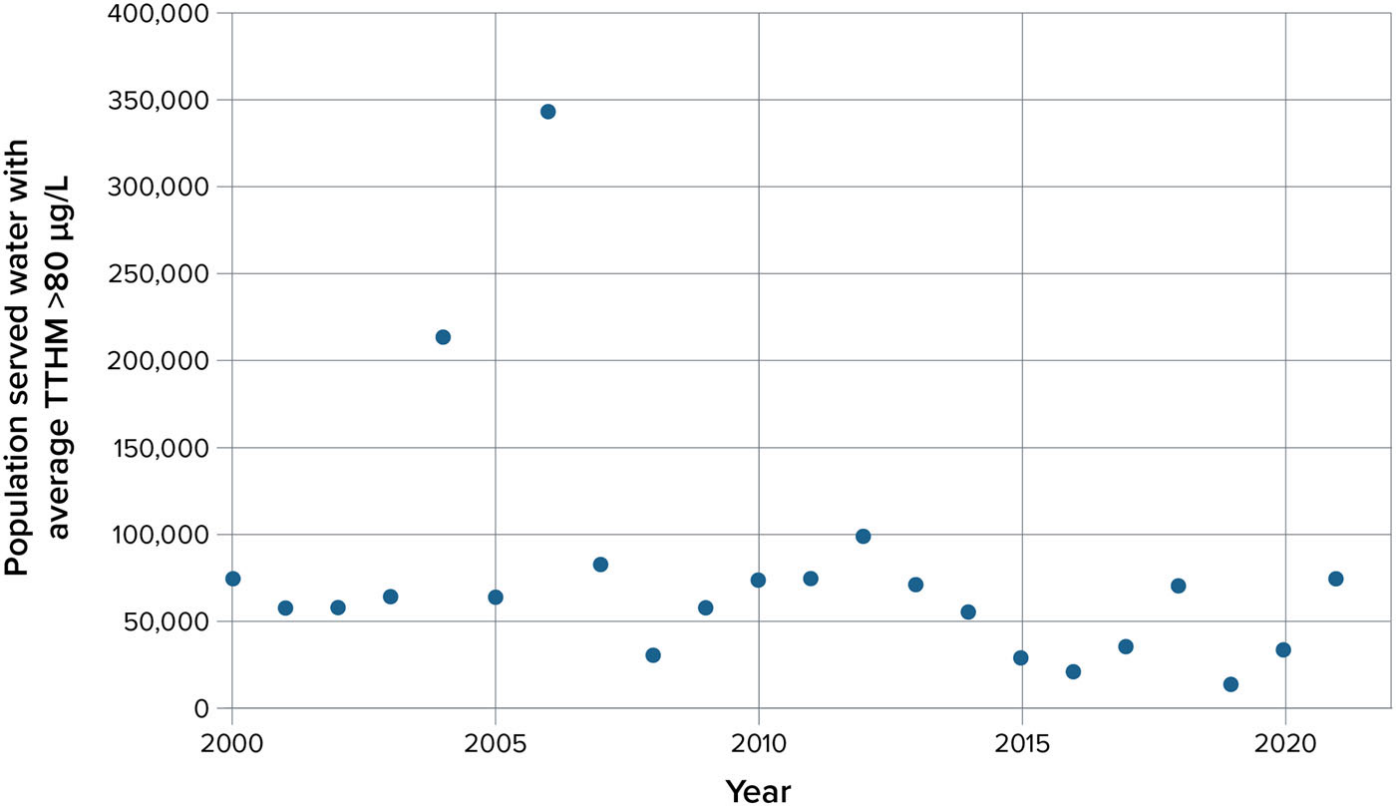
Annual population in New York State served by water supplies with average annual total trihalomethane concentrations exceeding 80 micrograms per liter. Data from New York State Department of Health (2022).^167^

As evident in Figure 10-9, there is no clear trend over time in changes in population exposed to high TTHM levels; this suggests there is not yet a strong linkage to a changing climate. However, multiple future climate‐DBP models predict increased MCL exceedances for TTHMs and HAA5.^168^, ^169^, ^170^, ^171^ The NYC DEP has been involved in a research project to develop advanced techniques to predict and control DBP formation potential in the New York City water supply system.^172^

##### 3.2.2.5 Other contaminants

Other harmful pollutants affecting the state's water quality include methyl mercury, per‐ and polyfluoroalkyl substances (PFAS) (including perfluorooctanoic acid [PFOA] and perfluorooctane sulfonic acid [PFOS]), lead, pesticides, fertilizers, road deicing salt, solvents, and pharmaceuticals, and microplastics. Unlike the contaminants discussed in prior subsections, these contaminants do not occur naturally and must come from human sources. This section focuses on their potential effects on potable water quality. The Ecosystems chapter addresses climate impacts on watersheds from agricultural runoff (e.g., fertilizers and pesticides), deicing salt, and CSOs.

Each of these contaminants has different toxicity levels, transfer pathways, and degradation mechanisms in the environment that climate hazards could exacerbate in different ways. For instance, recent research shows that high precipitation increased the mobility of atrazine (an herbicide), triclocarban (an antibacterial chemical), and seven antibiotics, resulting in higher in‐stream concentrations.^173^ Higher temperatures decreased detection, likely by causing faster degradation, but not without the concern of unknown degradation products.^173^ Thus, there is not always consistent information to understand exactly how the prevalence of these types of chemicals in drinking water might change.

PFAS compounds (per‐ and polyfluorolalkyl substances) have received increased regulatory attention as they have been detected more widely. PFAS compounds—sometimes referred to as “forever chemicals” because of their persistence—can negatively affect human and ecosystem health. PFAS can enter the environment from a number of sources, including manufacturing facility effluent, firefighting foams often used at airfields, WWTPs handling industrial effluent, and inactive landfills. PFAS monitoring has only become common in the last several years, and comprehensive data sets representative of drinking water across the state are just becoming available. The Federal Unregulated Contaminant Monitoring Rule 5 initiated data collection in 2023 on 29 PFAS substances and lithium; this data collection effort also now includes smaller systems.^174^ This new monitoring rule will help reveal the true extent of PFAS contamination of public drinking water across the country, while EPA has already acknowledged its disproportionate impact on small or overburdened communities.^175^

It is difficult to broadly generalize possible changes in PFAS mobilization and transport in a changing climate due to the diversity of sources and variety of pathways by which PFAS could move in the environment. Some studies suggest a possible decline in the mobilization of PFAS. For instance, modeling studies for northern temperate regions in China suggested that increases in precipitation could reduce concentrations of PFAS in inland water bodies and soils due to a dilution effect.^176^, ^177^, ^178^ Conversely, a study focused on the simulation of PFAS mobilization during rainfall events from fire training areas with a high concentration of PFAS in soils found that PFAS was consistently transported in runoff from poorly drained soils, with PFAS still potentially generated from soils for years after PFAS‐containing firefighting foam was last used.^179^

Additionally, increases in flooding could also make more stored material containing PFAS available to be mobilized. The *New York State Flood Risk Management Guidance for Implementation of the Community Risk and Resiliency Act* ^180^ instructs facilities that could cause harm to water bodies if flooded (e.g., those that store hazardous waste, chemicals, or floatable materials; water treatment plants) to remain out of the floodplain. However, this guidance does not always apply, such as when allowances are made for older facilities built before the current guidance; when a structure must remain in place because of access to utilities or lack of available land; or if facilities are repurposed.

Limited analysis of flood mobilization risk has been performed in New York State, but studies in other countries have identified vulnerable sites. Neuhold and Nachtnebel screened more than 1000 sites in Austria and found that roughly 30% of hazardous storage facilities are in flood‐prone zones. Screening of sites in England found that approximately 10% of solid waste management sites are in flood‐vulnerable areas.^181^, ^182^ In these cases, such sites were not typically active but were legacy sites that predate current environmental regulations. As part of the Inactive Landfill Initiative Program, New York State is inventorying inactive solid waste storage sites and quantifying risk to groundwater quality from contaminants such as PFAS.^183^ Despite this assessment of groundwater risk, there does not appear to be any concurrent assessment of the risk of mobilization of legacy waste due to flooding of the sites. The assessment team's preliminary mapping of these inactive sites suggests that approximately 13% (269 out of 2091) are located in or near floodplain areas with a 1% or greater annual chance of flooding (Figure 10-10). These 269 sites are spread across 52 counties. Six counties account for 41% of the sites, including Broome (48 sites), Erie (17 sites), Albany (12 sites), Saratoga (12 sites), Dutchess (11 sites), and Rensselaer (10 sites).

**FIGURE 10-10:**
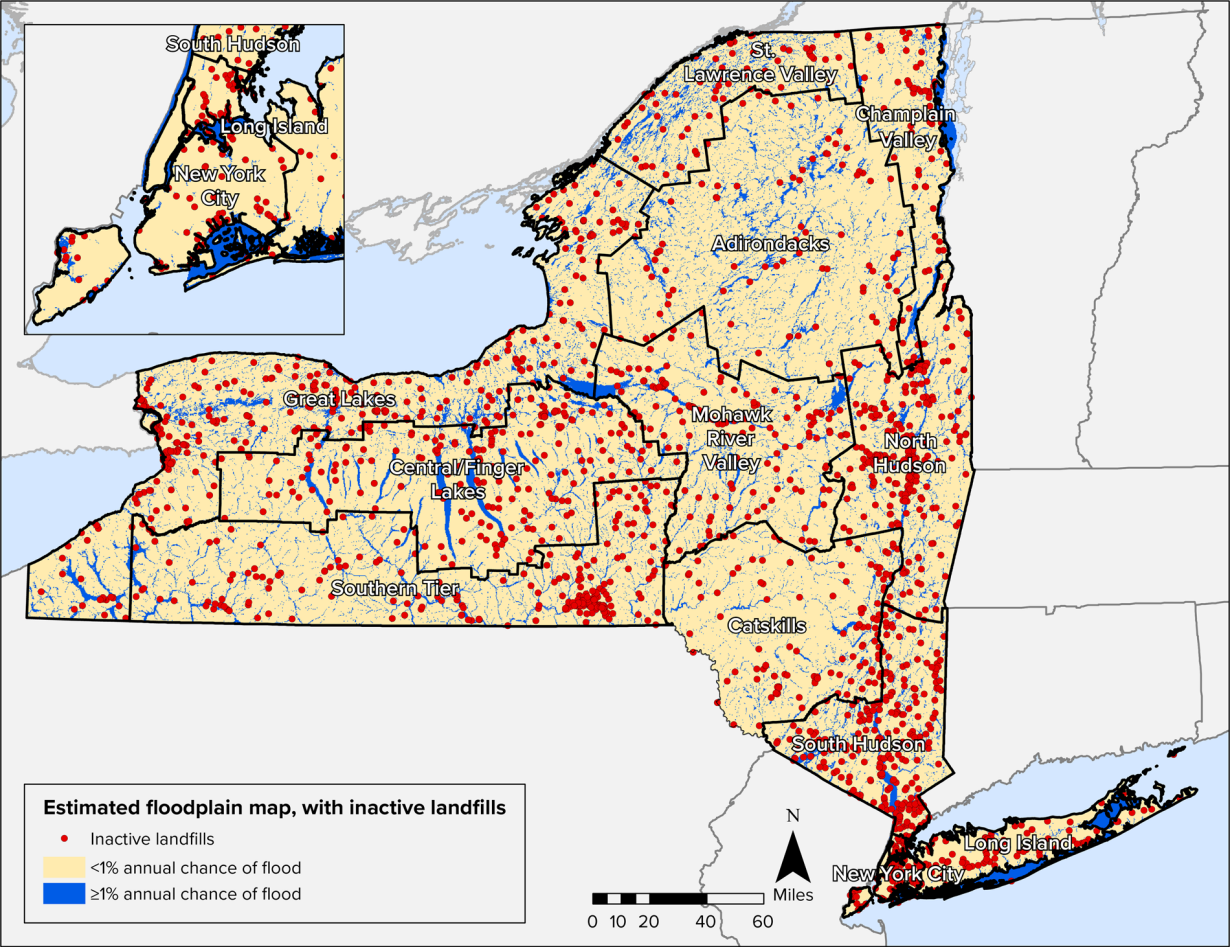
Inactive landfills and the 1% floodplain (1% or greater annual chance of flooding) in New York State. Data from NYSDEC (2023).^184^

### 3.3 Groundwater quantity and quality

Groundwater quantity and quality is important for drinking water, irrigation, and groundwater‐dependent ecosystems. Given the interconnectedness of groundwater quantity and quality, this section addresses climate impacts to both. It focuses on the Long Island aquifer system and other sand and gravel aquifers that collectively serve the majority of New York's groundwater users. Key climate hazards affecting groundwater quantity and quality are droughts, sea level rise, and precipitation increases. As with surface water in the previous section, the discussion of climate impacts to groundwater quantity and quality focuses primarily on potable uses.

#### 3.3.1 Long Island aquifer system

The Long Island aquifer system contains some of the most productive aquifers in the United States^15^ and provides water to more than 2.8 million people.^185^ In 2019, the aquifers were estimated to contain about 50 trillion gallons of fresh water.^186^ Surrounded by ocean, Long Island cannot readily tap into surface supplies of freshwater, and surface water quality has been degraded due to pollution,^15^ making groundwater the primary source of potable water. More than 1500 public supply wells pump about 425 million gallons of water per day, with additional groundwater pumped by private wells.^186^ The aquifer system includes three layers of fresh groundwater, separated by lignite, clay soils, and confining layers and surrounded by the salt water of Long Island Sound and the Atlantic Ocean.^187^ The primary source of aquifer recharge is precipitation, with anthropogenic contributions from wastewater return flow, stormwater inflow, and leaky infrastructure. Recharge rates vary across the island due to variation in soil and geologic conditions.^188^

Recent estimates suggest that precipitation can adequately replenish groundwater withdrawals.^15^ However, these estimates might oversimplify and overstate this balance, and current groundwater withdrawals in some areas could exceed recharge.^69^ Water tables have lowered by more than 10 feet over large areas of the western island, largely due to the additions of sewer systems that have eliminated wastewater return flows.^188^ In eastern parts of the island, water tables have risen by more than 2 feet. In drier years, water levels in the aquifer decline due to both a lack of precipitation for recharge and increased local groundwater pumping.^189^ In 2022, a sustained drought challenged Long Island water suppliers’ ability to keep up with groundwater demand.

Streamflows show similar patterns, with a decrease in streamflows in the west and an increase in the east.^188^ Maintaining a local balance between groundwater withdrawals and recharge is important for nearby streams, ponds, and wetlands. Overpumping can lower the water table. If the water table drops below the elevation of a stream, pond, or wetland, no recharge will reach these water bodies and they could dry up.

The balance between groundwater withdrawals and recharge is also important for groundwater quality. When withdrawals exceed recharge, the freshwater/saltwater interface can move inland (i.e., saltwater intrusion), affecting the water quality of wells near the interface. If a water supply well begins to have higher salinity, the well may no longer be viable for water supply use.^15^, ^190^ Saltwater intrusion has long been documented along the U.S. East Coast.^191^ A United States Geological Survey (USGS) study of the extent of existing saltwater intrusion along the eastern seaboard, including Long Island, found elevated chloride concentrations in wells of the Magothy aquifer along the southern barrier islands in central Long Island and in southern shoreline areas closer to New York City.^192^ Several public water supply wells have been shut down and abandoned along the southwestern coast of Long Island due to extensive saltwater intrusion.^193^ The freshwater/saltwater interface, once theorized to be miles offshore, is now located at the coastline, which could place groundwater supplies at the barrier islands, such as Long Beach, at risk.^193^

Water quality is also affected by past and present land use and agricultural practices. Chemicals such as fungicides, herbicides, and pesticides, including some chemicals that have been discontinued for more than 20 years, have entered aquifers via precipitation recharge.^194^ Groundwater pumping from deeper aquifers can draw these contaminants from the surface layers, potentially endangering deep groundwater quality.^195^

As sea levels rise and precipitation rates increase, the sustainable balance between recharge and withdrawal could change across Long Island. While sea level rise shifts the freshwater/saltwater interface inland, increased precipitation that results in increased recharge can shift the interface seaward, helping to maintain reduced salinity levels within the aquifer. Due to concerns over water quality and aquifer sustainability, USGS has partnered with NYSDEC to model the complex groundwater processes within the aquifer system. Since 2016, this effort has improved the hydrogeologic mapping and modeling of the aquifer system. The effort continues to assess water quality, saltwater intrusion, and effects on freshwater and coastal habitats, and helps to predict likely outcomes of future management decisions and a changing climate.^186^, ^188^, ^194^, ^196^, ^197^

The Shinnecock Nation and Unkechaug Nation populations remain heavily reliant on individual household groundwater wells to meet drinking water needs. They face additional stressors due to sea level rise, including saltwater intrusion and inundation of existing Tribal territories.^61^ In 2014, USGS partnered with the Shinnecock Nation to establish 17 observation wells on Shinnecock territory to analyze and assess the vulnerability of coastal ecosystems and potable water supply.^198^

#### 3.3.2 Sand and gravel aquifers

Other municipalities across the state also rely mainly on groundwater. They include Schenectady, Batavia, Endicott, and Johnson City, Cortland, and Jamestown (as identified by principal aquifers in Figure 10-2), as well as other smaller communities. Nearly all of these municipalities are located in areas in which retreating glaciers dropped thick deposits of sand and gravel tens of thousands of years ago.^187^ These sand and gravel deposits have large pore spaces that can store sizable amounts of water and readily transmit water to high‐yield wells. The overall amount of stored water depends on the extent and depth of these deposits. Because many of these groundwater supply systems are the primary water source for their community—referred to as sole source aquifers—they have undergone extensive geologic mapping as part of a cooperative program between USGS, NYSDOH, and NYSDEC. For these mapped aquifers, the amount of stored water can be many times the daily demand, providing extensive buffer against extended dry periods. For aquifer systems in which the ratio of recharge to well withdrawals have been studied, such as the Cortland aquifer, recharge remains greater than withdrawals, even during dry periods.^199^ Based on this anecdotal information, as well as more formal studies like the USGS study in Cortland, it appears that sand and gravel aquifers should remain a reliable water source even during possible future periods of more intense, short‐term summer drought, with storage buffering any deficits in recharge.

In cases where a well is situated near a sizable river, pumping the well can cause induced infiltration such that most well water is being drawn from the river. Communities with sizable induced infiltration include Schenectady, Johnson City, and Endicott. For example, Endicott's water system specifically includes a Ranney well—an approximately 10‐foot diameter shallow well^200^—situated near the banks of the Susquehanna River with a yield of about 4000 gallons per minute.^201^ In these cases, the well systems operate more like run‐of‐river systems and could be sensitive to low river flows.

#### 3.3.3 Private wells

About 2.5 million people in New York State rely on private wells for their water (Figure 10-3). Modern wells typically consist of a 6‐inch diameter steel casing designed to keep the well hole open and keep possible contaminants near the surface from entering the well. A submersible pump pushes water through heavy‐duty plastic tubing to the surface where it connects to the home's plumbing system. Since 2000, drillers have submitted well‐completion records to NYSDEC, which maintains information on flow rate and water depth in a database.

Because wells are often only drilled to a depth that provides sufficient water at the time of drilling, conditions drier than those during the period of drilling could lead to the groundwater table dropping below the well depth and the well going dry. A review of well construction data in California found that one in five wells in the Central Valley had gone dry during severe, multiyear droughts.^202^ While California is an extreme case given the severity of its recent droughts, and there is no indication this severity would occur in New York State, the situation provides some indication that the widespread presence of dry wells is not out of the question. Interviews with 41 well owners in Arkansas, Indiana, and Oklahoma^203^ after a 2012 drought found that while few respondents had their own well go dry, many knew someone who had. An analysis in the northeastern United States found that well completions reported by well drillers increased during periods of drought when controlling for new housing construction, suggesting that new wells had been drilled to replace dry wells during these drought periods.^204^ The study observed well failure in about one in 1000 homes in a region experiencing drought. While homeowners should slowly replace the shallowest, most vulnerable wells as new droughts occur, the possible effects of drought on groundwater levels may become more severe. Even if droughts occur with the same duration and intensity as in the past, increased evaporative water loss brought by increasing temperatures and longer growing seasons due to climate change could decrease well recharge further and result in an increased number of private wells going dry.

### 3.4 Vulnerabilities

#### 3.4.1 Vulnerabilities to quantity of water available

As described in Section 3.2.1, communities that draw water from the Great Lakes, other large lakes, reservoir systems with extensive storage, or groundwater aquifers with extensive reserves are at the least risk of facing supply shortfalls. At somewhat higher risk are systems with little storage (e.g., run‐of‐river systems) that are sensitive to even brief periods that are drier than normal. Increased evaporative water losses in a changing climate could exacerbate any short‐term precipitation deficits and cause short periods of limited supply even if conditions are on average wetter over the full year or longer.

Because of the certain continued increase in sea level,^72^ communities with water supplies sensitive to sea level are in particular need of additional information on potential future risks to water availability. Sea level rise poses a risk to the seven communities along the Hudson River (with an aggregate population of over 100,000)^205^ that draw water directly from the river and depend on the position of the salt front. The position of the salt front also indirectly affects water allocations to New Yorkers in the Delaware River Basin. A large portion of New York City's water supply watersheds fall within the Delaware River Basin. Thus, water diverted to supply the New York City system is water diverted away from the Delaware River and its other downstream users such as the city of Philadelphia, Pennsylvania. A 1954 Supreme Court ruling^206^ established permissible withdrawals and minimum required flows within the Delaware River. While the agreement established by this ruling and the formation of the DRBC have successfully coordinated water withdrawals for the last 70 years, the continued success of coordinating water availability to both New York City and Philadelphia depends on state, regional, and local water resource authorities continuing to prepare for shifts from historical conditions.

Sea level rise also poses a threat to coastal aquifers that may be subject to saltwater intrusion. An ongoing USGS study (originally slated for completion in 2023) will provide a better understanding of possible changes in water supply from aquifers on Long Island. Given that groundwater remains the only potable water source for nearly all of Long Island, there is a critical need for continued studies to better understand the potential risk associated with saltwater intrusion due to increasing sea level.

#### 3.4.2 Vulnerabilities due to changing demand

It is critical to ensure that efforts to build resilience to climate change will also remain sufficient in the face of other changes such as population growth in certain communities. As noted earlier, about 2.5 million New Yorkers, largely in rural areas, rely on their own private wells (Figure 10-3).

In some communities, there are efforts to move people from homeowner wells to more reliable community water supplies. For instance, the Saint Regis Mohawk Tribe in the Akwesasne region operates its own water treatment plant that draws from the St. Lawrence River.^207^ Residents in Akwesasne relied on homeowner wells for many decades, but most have transitioned to using the centralized water system. The plant capacity is more than 1.2 million gallons per day, but typical use is closer to 800,000 gallons per day.^208^ Therefore, when possible, the utility seeks to connect Tribal citizens on well water to the public water system through the installation of new water lines.^208^ However, Tribal Nations face persistent challenges to meeting increasing water infrastructure demands as populations grow. For example, Akwesasne has a population growth rate of 3.6% per year.^209^ Programs to encourage regional economic development could also lead to higher growth than has been observed in recent decades. For instance, Tribal Nations have used federal funds extensively to remediate former industrial sites with chemical contamination in the St. Lawrence region, opening the door to new development,^210^ which could put pressure on existing water infrastructure.

#### 3.4.3 Vulnerabilities to quality of available water

While the quality of treated water from public water supplies is extensively monitored, there is not always consistent monitoring of ambient water quality. Concerns include nutrients and turbidity, and parameters as simple as temperature. As of 2018, 38% of rivers and streams and 60% of lakes and reservoirs across the state remain unassessed.^211^ Gaps in consistent, long‐term water quality monitoring data across the state can impede understanding of general water quality trends that could affect all water bodies, whether used for water supply or not. Additionally, even when not used as a public water supply, some lakes still serve as a water source for individual homeowners operating their own pump and treatment systems. Much of the lake monitoring that occurs within the state occurs as part of the CSLAP. Local volunteers sample the lakes participating in CSLAP biweekly from mid‐June to October^211^ and samples are analyzed by professional laboratories. However, lakes without an organized, well‐resourced lake association may not be routinely monitored. This is a major concern for lakes in low‐income areas, including urban and suburban lakes.

## 4 WATER INFRASTRUCTURE IMPACTS AND VULNERABILITIES

Aging and deteriorating water infrastructure is already highly susceptible to nonclimate hazards, underscored by frequent instances of water infrastructure failures in the state and nationwide.^212^, ^213^, ^214^ Climate change compounds the potential for structural, functional, and operational consequences due to climate hazards.^215^ The upgrades needed to make water infrastructure more resilient are extremely costly, creating vulnerabilities for systems that treat and convey water throughout New York State. This section highlights climate impacts to potable water supply infrastructure and wastewater treatment and conveyance infrastructure.

### 4.1 Water supply infrastructure and operations impacts and vulnerabilities

Potable water requires substantial collection, treatment, and distribution infrastructure. Multiple potential climate‐related impacts to these facilities in New York State could reduce the reliability of public water supplies. While infrastructure upgrades for climate resilience are often possible, these updates can be expensive, and utilities may ultimately pass this expense to water users through increased water rates.

#### 4.1.1 Impacts to water supply infrastructure

Potable water treatment infrastructure and delivery often require large quantities of power to drive pumps and other mechanical equipment. Power outages due to extreme weather events could disrupt water treatment and delivery.^216^ The current *Recommended Standards for Water Works*, commonly referred to as the 10 State Standards, indicate that water treatment facilities should have auxiliary power sufficient to meet average daily demand, and that all water supply facilities and water treatment processes be protected to at least 3 feet above the 100‐year flood or maximum flood of record.^217^ However, these standards only apply to new construction; existing facilities may not have full backup power. Extreme heat could also stress sensitive electrical equipment at treatment or pumping facilities.

Another concern is the disturbance or rupture of piping, especially in the distribution system. Flooding could be an issue for facilities located in flood zones, with a recent study estimating that by the late 21st century, the 100‐year flood level could occur as frequently as annually in the northeastern United States.^218^ There have also been instances in other parts of the country of pipelines rupturing from the weight of floodwaters. Pipe failure can also be caused by temperature or soil moisture variations that affect pipe materials or cause movement in soils around the pipes. Iron pipe failure is most often seen in winter due to heave and soil expansion caused by soil freezing. Climate change may lead to an increased frequency of extreme freeze‐thaw cycle events that may lead to increasing instability of conventional water pipe systems.^219^ However, there is also the possibility for breaks due to dry weather as soils dry and move. Polyethylene pipe is more resistant, and implementation of this pipe material as systems are upgraded would reduce future sensitivity to climate.^220^

#### 4.1.2 Impacts to dams

New York State has more than 100 large dams and thousands of small dams,^221^, ^222^ including dams used to impound water supply reservoirs. Many of these dams are in disrepair and in need of upgrades.^222^ The design of most dams allows high waters to flow around the dam using an emergency spillway. For most dams, failure can occur if water overtops the dam. Therefore, proper sizing of the spillway is essential for dam safety, particularly considering excessive precipitation and extreme weather events resulting from the changing climate. For smaller dams with limited impact to life or property, if failure occurs, the *New York State Guidelines for Design of Dams* require their spillway design to pass the 100‐year flood (i.e., 1% annual exceedance probability).^223^

For large dams that could result in extensive destruction if they were to fail—such as the large reservoir dams classified as high hazard (hazard Class C)^224^—the spillway must be designed to pass the so‐called probable maximum flood (PMF). The PMF is the flood that can be expected from the most severe combination of critical meteorologic and hydrologic conditions possible, with the flow resulting from the probable maximum precipitation. The PMF is the hypothetical worst‐case scenario, typically outside the observed record in most places. For instance, rainfall amounts leading to a PMF could be upward of 11 inches of rain in 24 hours.^225^

Climate change is expected to increase the probability of extreme storms, changing the magnitude of both 100‐year storm events and possibly the PMF.^226^ This assessment's analysis of climate projections concurs with high confidence that several types of storms will become more intense in New York during the 21st century.^72^ However, very large precipitation events depend on several factors, many of which are geographically dependent, such that regional analyses are needed.^227^ While detailed analyses on changes in PMF have been performed for the southeastern United States,^228^ parts of Canada,^229^ and the western United States,^227^ researchers have not yet considered changes in PMF specifically in the Northeast. This remains an active area of research and discussion within the dam safety community, with some states already proactively adjusting their PMF estimates. For example, New Mexico and Colorado have increased their current PMFs based on climate change analyses.^230^

While large dams are often important for establishing water supply reservoirs, there are thousands of smaller dams throughout the state that create shallow lakes or ponds. While the possible failure of these dams may not greatly affect the volume of potable water in most cases, they can affect water quality if their failure results in the flushing of stored sediments or the loss of impounded waste. For instance, many dairy farms store manure slurry in large tanks or holding ponds prior to spreading on farm fields. While failure is rare, a large manure pond failed in Lewis County, New York, in 2005; this failure led to an extensive fish kill on the Black River.^231^ While structural failure, not climate‐related impacts, caused this 2005 event, the frequency of small dam failures could increase due to a combination of aging infrastructure and increased high‐precipitation events that lead to rapid increases in stored volume within impoundments.

#### 4.1.3 Vulnerabilities

There are several vulnerabilities associated with water supply infrastructure, depending on the source of the water supply, limitations of community resources, and the age of the infrastructure. Some water supply infrastructure vulnerabilities result from direct exposure to climate hazards. This includes communities whose water resources are threatened by sea level rise. For example, the Shinnecock Nation on Long Island has identified sea level rise as a risk to facilities and utilities on Shinnecock Neck.^232^

The water supplier's financial and technical resources also influence vulnerability. Large water suppliers, such as major metropolitan utilities, have permanent technical staff, well‐developed infrastructure, and often multiple options for accessing water (e.g., surface water, groundwater reserves, interconnections). These larger entities have a greater capacity to plan and adapt to meet demands and treat water if changes in quality occur. Based on NYSDOH's 2022 listing of community water systems, New York has more than 1300 systems serving fewer than 100 people (over 50,000 people total) and another 1300 systems serving between 100 and 1000 people (more than 460,000 people total). The smallest systems often serve manufactured housing communities or apartment buildings. The systems serving between 100 and 1000 people typically serve small towns and villages.^233^ Small systems must often focus more on day‐to‐day operations and less on long‐term planning for any possible changes in water availability or supply. Climate adaptation incurs immediate costs, while benefits may not accrue for many years, and utilities may be competing with other needs requiring public funding that may take priority, such as housing or public health.^234^

Systems with fewer resources could experience larger water rate increases per capita if additional treatment or new infrastructure is necessary to deal with the impacts of climate change. For instance, as noted earlier, some water systems may see increases in turbidity due to more high‐intensity storm events or additional biological growth. While treatment facilities can remove elevated turbidity, doing so could require facility upgrades and involve increased operational costs. While water suppliers serve all residents within the utility's service area boundaries regardless of race or income level, there can still be a question of whether all people have reliable access to piped water. If costly upgrades to address climate impacts result in substantial water rate increases, some customers could lose access due to failure to pay water bills. While most municipalities in New York do not shut water off when residents fall behind (instead taking out liens on customer property), some places—such as the city of Buffalo—do allow shutoffs. Recent investigative reporting found that 10% of homeowners in Suffolk County were behind on payments, while about 3% in Monroe County were behind.^235^

To give a sense of the cost of water, 2016 estimated water rates for a household of four were $730 per year in New York City and around $480 per year in other large metropolitan areas such as Buffalo, Rochester, Syracuse, and Albany.^8^ The highest rates were for areas in Rockland and Westchester County served by private water companies, at costs of more than $1300 per year. EPA's affordability benchmark considers a water bill larger than 2% of annual household income to be unaffordable.^236^ Thus, in New York City or other large metro areas, household incomes would need to be above $36,500 and $24,000 per year for a typical water bill to be considered affordable. Around 20% of households in New York State have incomes below $30,000 per year.^237^ While some of these households may have fewer than four people—and lower water bills than reported above—these numbers suggest that a sizable fraction of the state may face water affordability issues.

Of particular concern are possible increases in water rates that exceed increases in pay. These increases in water rates can come from required new investments (including investments needed to manage a changing climate and water trends) as well as loss of customer base in areas with diminishing population. In general, smaller utilities are often more sensitive because there are fewer customers to spread fixed costs to.^8^ Thus, water utilities and other decisionmakers must carefully consider the affordability of infrastructure investments intended to increase resilience to climate change.

Additionally, there are increasing concerns about safety and security risks posed by dams that may be exacerbated due to climate change. According to the Division of Homeland Security and Emergency Services, “there are approximately 400 high‐hazard (Class C) and nearly 700 moderate‐hazard (Class B) dams that pose a threat to jurisdictions in the event of a dam failure” in New York State.^238^ Dam failure is influenced not only by age but also by hydroclimatic and structural risks.^239^ Additionally, of the 407 dams in the state classified as high‐hazard, 207 have had engineering assessments filed to NYSDEC.^222^ These dams could be further evaluated for their ability to handle higher levels of water and update maintenance and operation plans. For example, planned water releases may need to take place before a forecasted extreme rainfall, so adequate water levels are maintained.

An emerging trend for addressing environmental injustices exacerbated due to dams is assessing dams for decommissioning. An example is the Hogansburg Hydroelectric Dam removal led by the Saint Regis Mohawk Tribe, which also led to the return of land to the Tribal Nation and habitat restoration for fish species of cultural importance.^240^ (Refer to the Ecosystems chapter for additional discussion about culturally important ecological resources.) Challenges related to resource limitations can lead to hesitancy among water utilities and utility decisionmakers (e.g., local elected officials, boards) to adopt new, climate‐resilient practices. Such hesitancy is well‐documented beyond the water sector.^234^, ^241^, ^242^, ^243^, ^244^ Resource and technical limitations related to understanding about the significance of climate change and how it will affect specific communities and infrastructure further compounds this issue.^234^, ^245^ Within the water sector, surveys have indicated that utilities view avoiding complaints and incidents as a measure of success,^246^ rather than being at the forefront of innovation.

### 4.2 Wastewater infrastructure impacts and vulnerabilities

Sewerage and drainage systems are critical for maintaining public health, preventing property damage, and controlling water pollution. Due to a combination of locations in low‐lying areas and age, these systems may not perform as designed as weather patterns and sea levels change in the future. Sea level rise and extreme weather events are of primary concern as they could affect service reliability, regulatory compliance, consumer perception, and costs of sewerage and drainage systems.

#### 4.2.1 Impacts to wastewater treatment plants

NYSDEC records list more than 2000 WWTPs in New York State.^247^ Of these, approximately 70% are industrial facilities and 30% are municipally owned. NYSDEC estimates that the municipal WWTPs serve more than 14 million residents, with combined design flows of more than 3.7 billion gallons per day of treated wastewater.^248^ WWTPs provide critical protection of the state's water resources by limiting the levels of pollutants discharged to water bodies, but no data are available for the design flows for the state's industrial WWTPs. Climate change has the potential to cause a variety of impacts to the effective operation of these systems.^249^ The two broad categories of impacts are physical damage from facility flooding and operational impacts that reduce pollution management effectiveness.

##### 4.2.1.1 Facility flooding

Sewerage collection systems are generally designed to maximize the use of gravitational flow and are often located in low‐lying areas near water bodies where the system can discharge treated wastewater (Figure 10-11). Flooding can cause multiple impacts to a WWTP, including damage to structures, electrical equipment, and mechanical equipment. Flooding can also lead to discharges of untreated wastewater to the receiving water body. Further, the plant could be out of service for some time after a flood event to repair damage, thus reducing effective treatment capacity.

**FIGURE 10-11:**
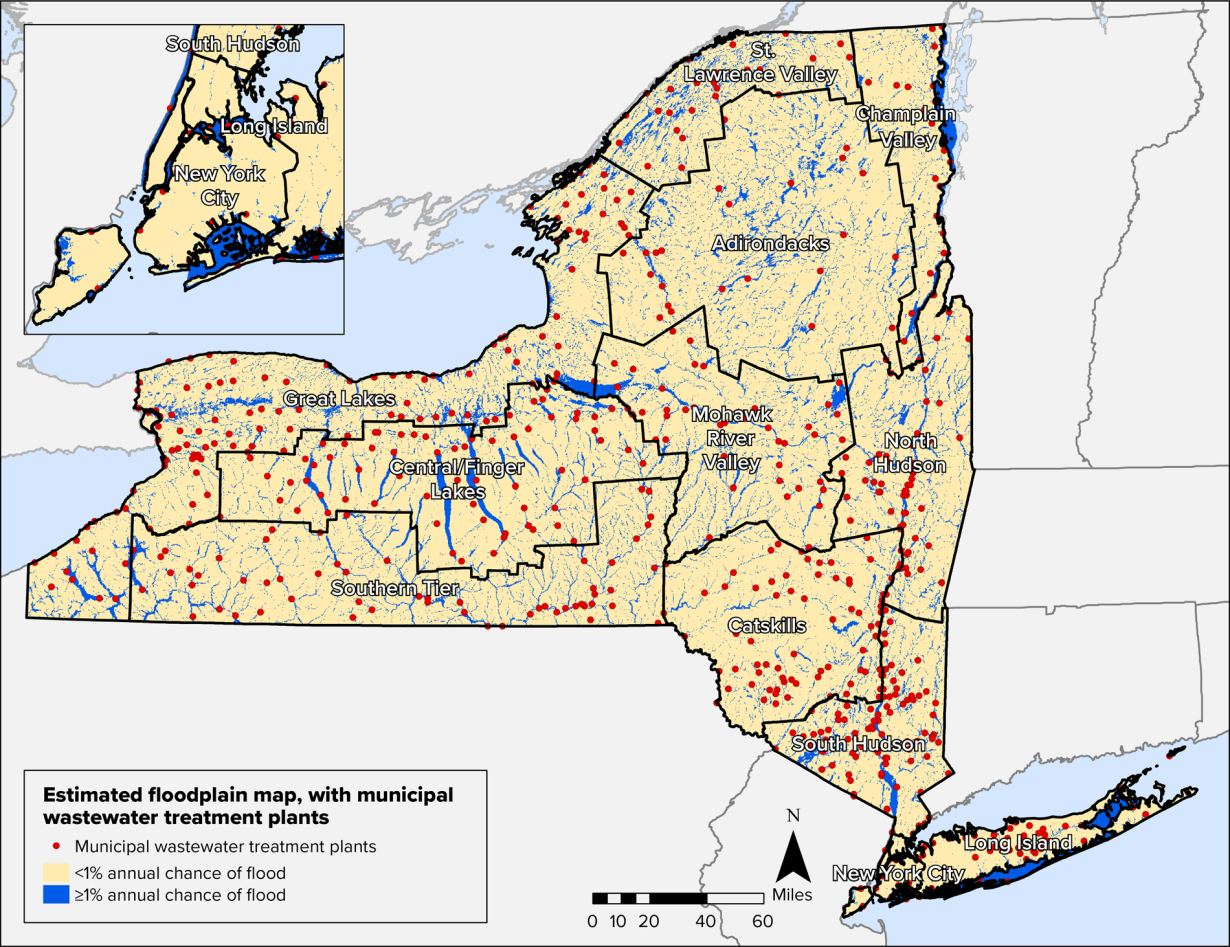
WWTPs and the 1% floodplain in New York State. Geospatial analysis of the 635 municipal WWTPs in New York State found that 182 were located within the defined 100‐year (1% or greater annual risk) floodplain. Data from NYSDEC (2022).^247^

Flood risks for inland areas depend on the unique interaction of climate and topographic conditions at each facility. Great Lakes water levels have been unpredictable in recent years, reaching unexpected near‐record highs in 2017. A survey by the IJC in 2019 found that 30% of treatment facilities located on Lake Ontario or the St. Lawrence River experienced some negative operation impacts due to high water levels.^250^ However, some of the largest facilities on the Great Lakes, such as those serving Rochester and surrounding towns, saw little impact (Chung C, Department of Environmental Services, Monroe County [2022, August 16, Personal communication]). Numerous WWTPs in the state are located on rivers and streams, and there are multiple examples of damage to these facilities. For instance, in 2011, the Binghamton‐Johnson City WWTP severely flooded during Tropical Storm Lee and critical equipment was damaged.^251^ In August 2021, the Tully WWTP in Onondaga County flooded during intense localized storm events.^252^ While the large Binghamton‐Johnson City WWTP was not fully rehabilitated until nearly 2019, the Tully WWTP came back online within several days.^253^ Despite the presence of prior damage at facilities, past flooding is not necessarily an indication of increased frequency of future flooding in riverine systems.^254^, ^255^

WWTPs with the highest risk are arguably those located on tidal waters facing the potential combination of storm surge, continued sea level rise, and local flooding due to intense rainfall. As an example, Superstorm Sandy damaged numerous WWTPs in New York State that resulted in billions of gallons of sewage overflows; repairs were estimated at nearly $2 billion.^256^, ^257^ An assessment of possible damage to New York City's WWTPs found that the combined effects of sea level rise and a surge 8 feet above normal would affect all 14 of the city's WWTPs.^258^ This study was limited by the fact that it only evaluated facilities’ sensitivity to various water level increases but did not evaluate the future probability of such increases. Studies that only assess future impacts associated with sea level rise and storm surge likely underestimate future risks, as increasing extreme rainfall^259^ and rising groundwater tables create compounding risks.^260^

In low‐lying coastal communities, the unconfined shallow groundwater table can rise with sea level rise. It has long been observed that wells near coastal shorelines have water levels that fluctuate with the tides or other oscillations in ocean elevation. The continuing rise in sea level will permanently shift these groundwater levels upward; the degree of groundwater table rise and the inland extent of the rise is dependent on soil characteristics, topography, and potential drainage mechanisms such as tributaries.^260^, ^261^, ^262^, ^263^ Rising groundwater tables are a concern in areas that have groundwater near the land surface. For instance, modeling of changes in groundwater depth on the Rockaway Peninsula in New York City found that with a 0.75‐meter increase in sea level, the groundwater table would approach the soil surface over large portions of the peninsula.^260^ When the groundwater exceeds the soil surface, it presents as emergent groundwater, which can create a flood hazard. The rising groundwater table can also cause septic system failures, infiltrate sewers, intrude into basements, and degrade built infrastructure long before it emerges above ground.^264^, ^265^, ^266^, ^267^

Even WWTPs that seem to have limited risk based on the most recent flood mapping could become more vulnerable in the future. For example, Figure 10-12 shows that most of the site for the Albany County Water Purification District North Plant along the tidal portion of the Hudson River lies above the current Federal Emergency Management Agency (FEMA) Special Flood Hazard Area, but is within the shaded Zone X. The Special Flood Hazard Area has an annual flood recurrence of 1% (100‐year return period) and Zone X has an annual flood probability of 0.2% (500‐year return period). (FEMA is using the term Special Flood Hazard Area [formerly the 100‐year floodplain] for areas at risk of flooding due to a 1% annual chance flood event and the term Moderate Flood Hazard Area [formerly the 500‐year floodplain] for areas with a 0.2% annual chance of flooding. Moderate flood hazard areas are denoted as either Zone X or Zone B.) Increases in these flood probabilities, as a result of either increased extreme storms or sea level rise in tidal zones, will increase the flood vulnerability of this and other WWTPs in the state.

**FIGURE 10-12:**
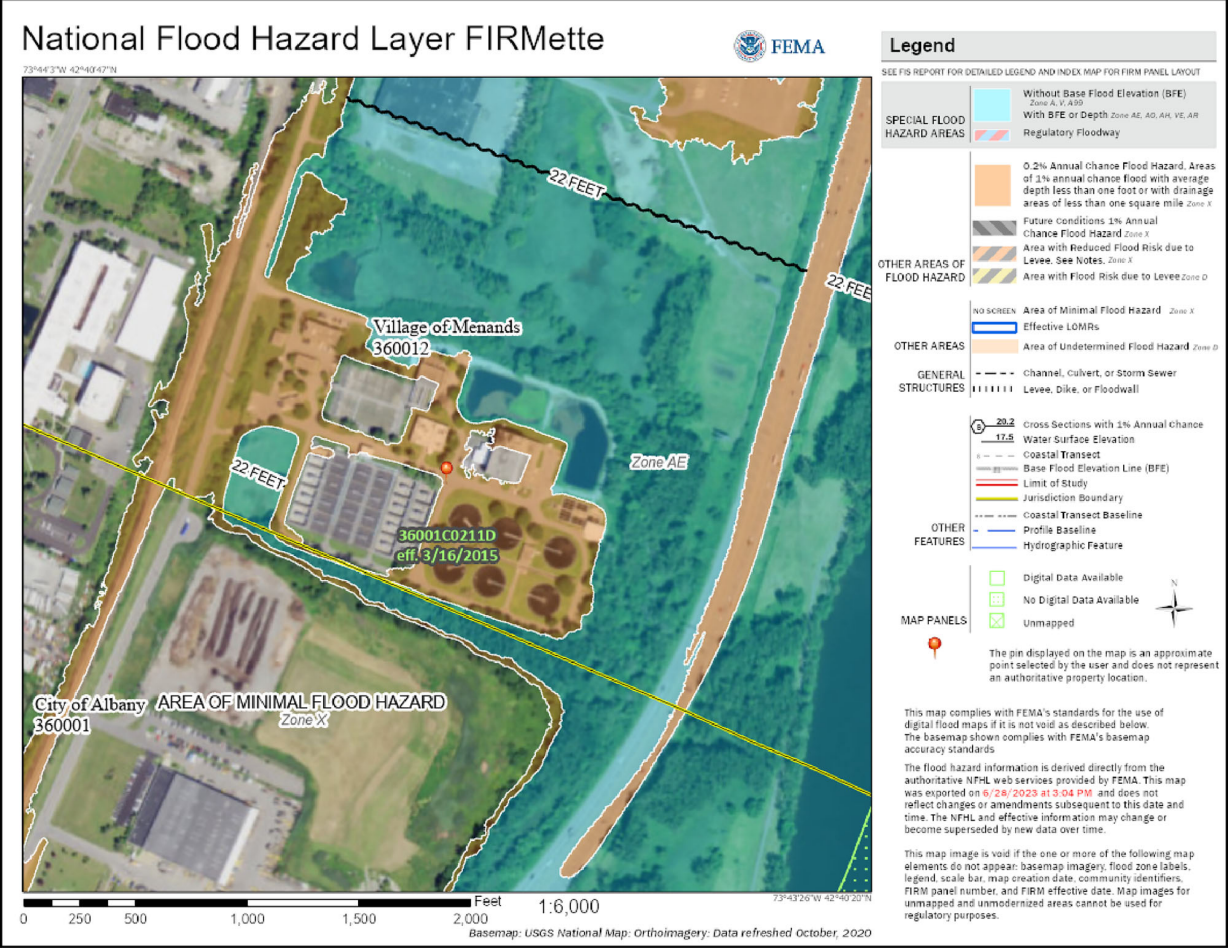
Screen capture of FEMA web tool showing Albany County Water Purification District North Plant along the Hudson River with the FEMA Special Flood Hazard Area and shaded Zone X. Image from FEMA (2023).^269^

Pump stations are another key piece of wastewater infrastructure that can sustain damage from flooding. While sewer systems are designed to work primarily by gravity‐driven flow, there are often geographic and topographic constraints that require pumping from low elevations in the sewer system. Pump stations lift wastewater to a higher elevation such that it can continue its journey to a treatment plant. During Superstorm Sandy, 42 of 96 pump stations in New York City became at least temporarily inoperable. Half of these losses of service resulted from power outages.^268^

##### 4.2.1.2 Operational impacts

Operational impacts to WWTPs are wide‐ranging, depending on the projected changes in temperature extremes, precipitation, and sea level rise. Most WWTPs operate with a combination of physical, biological, and chemical treatment systems intended to maintain proper treatment year‐round under a variety of conditions. Weather extremes and sudden changes in weather conditions can make it more difficult to maintain the proper system balance. Temperature extremes, predominantly high temperatures, could negatively affect the resilience of temperature‐sensitive electrical equipment (e.g., variable frequency drives for pumps).^270^

Extreme precipitation and rising groundwater tables can cause problems for WWTPs because of the unintended collection of stormwater through infiltration and inflow (I&I) into the sanitary sewer system.^271^ The extra volume of water can cause sanitary sewer overflows in the collection system and can exceed the capacity of the treatment plant, both of which can cause the release of untreated or partially treated sewage into the environment. Prior studies have shown that I&I volume is positively correlated with precipitation intensity.^272^, ^273^ Substantial I&I from high‐intensity storms during hot, dry summer conditions can stress the biological systems,^274^ not giving the microbial population time to adapt to the rapid change in concentrations.^249^ From a hydraulic capacity perspective, there is a risk that the biomass that feeds on the waste in the wastewater will float out. When this occurs, it causes both receiving water pollution and the loss of an important component to treat further incoming wastewater.

Sea level rise can cause operational problems for WWTPs that discharge to tidal areas. Wastewater treatment entails multiple processes that are typically constructed to maximize gravity flow and minimize pumping. The hydraulic grade line is an important factor that ensures the proper flow and residence time for each of the processes (Figure 10-13). As the elevation of the discharge point increases, the hydraulic grade line can change and reduce the efficacy of one or more treatment processes. The level of vulnerability for an individual facility will depend on the height difference between the final processes and the receiving water body as compared to expected changes in the tidal range. Hydraulic grade line impacts could also occur in riverine systems from flooding. However, such impacts may be less likely because there are typically larger topographic differences along rivers and because riverine flooding is relatively short in duration compared with the more permanent changes anticipated for sea level rise.

**FIGURE 10-13:**
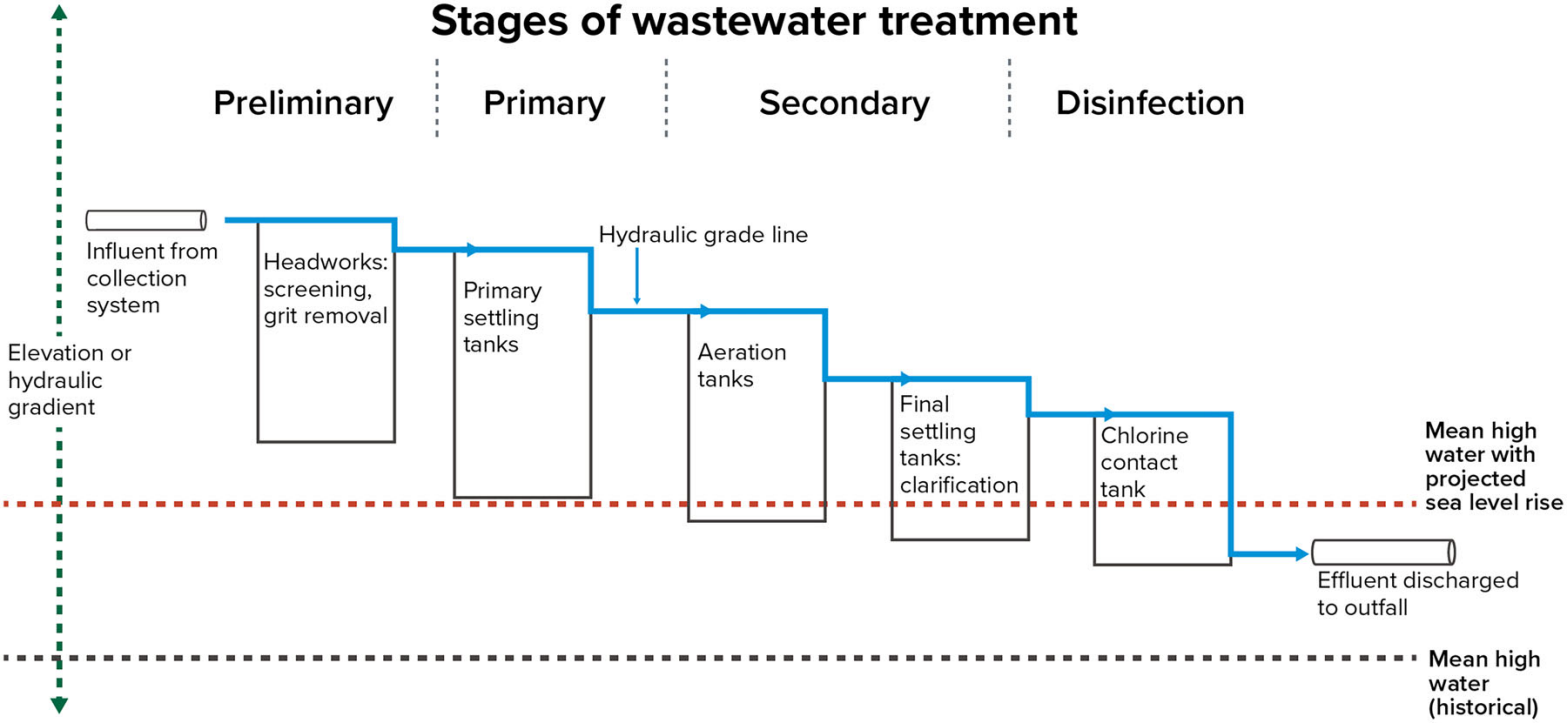
Hydraulic grade line example. Figure developed by the authors of this assessment.

##### 4.2.1.3 Considerations for WWTPs serving Tribal Nations

The Indian Health Service's Sanitations Facilities Construction Program assists with the provision of wastewater treatment systems servicing Tribal Nations in New York State, as mandated under the Indian Sanitation Facilities Act, Public Law 86‐121. However, not all Tribal Nations in New York currently have access to a WWTP, and they may rely heavily on septic systems, in part due to the cost and feasibility of constructing or extending services from a WWTP to small communities. Climate change is expected to further exacerbate health risks and from water quality problems and lack of adequate infrastructure that Tribal Nations already experience higher levels of, compared with other communities.^275^

#### 4.2.2 Impacts to CSSs

A CSS collects both rainwater runoff and municipal sewage into one collection system. Under normal dry‐weather conditions, wastewater is transported to a wastewater recovery facility for treatment before being discharged to a water body. During heavy rainfall or snowmelt events, the volume of wastewater and rainfall or snowmelt runoff can exceed the capacity of the CSS or treatment plant. When this occurs, untreated or partially treated human sewage and industrial waste mixed with stormwater discharges directly to nearby streams, rivers, and other water bodies. These untreated discharges are referred to as CSOs.

In New York State, 43 communities have permits to own and operate a CSS with CSOs, and there are approximately 816 CSO outfalls.^276^ Of the current CSO outfalls, 72% are located in four metropolitan areas: New York City (49%), Albany (10%), Buffalo (7%), and Syracuse (6%). Eighty‐nine of the state's approximately 700 facilities have some portion of their wastewater collection systems combined with stormwater collection. These 89 systems serve more than 10 million residents—roughly half the state's population.^248^ Given the large population served, CSOs are a primary pollution concern across the state.

Changes in precipitation, extreme weather, and sea level rise affect the function of CSSs and influence CSO frequency. Because CSSs transport stormwater runoff, changing rainfall patterns and extreme events could cause volumes that exceed the systems’ existing capacity. In August 2014, a flash thunderstorm overwhelmed Albany's CSS with approximately 3 inches of rain in less than an hour,^277^ causing CSOs. Sea level rise factored into the new design and increased construction costs of tide gates at the Hudson River CSO outfall in Albany.^278^ Albany's climate change vulnerability assessment acknowledges that the two wastewater treatment facilities near the Hudson River could experience more frequent flooding due to sea level rise and more frequent precipitation.^279^

While the preferred solution to CSSs is to rebuild sewer networks such that stormwater and sanitary sewers are separate, this can be prohibitively expensive and difficult to accomplish. As an alternative, regulators have worked with CSS municipalities to develop long‐term control plans (LTCPs). The goal of LTCPs is to identify the appropriate CSO controls necessary to achieve water body–specific water quality standards, consistent with EPA's CSO Control Policy and the water quality goals of the Clean Water Act. Projects in LTCPs may include some sewer separation but can also include increasing capacity at WWTPs, closing certain overflow outlets, diverting runoff away from storm drains and enhancing infiltration and recharge, operating street sweepers or skimmer boats to remove floating trash, and directly treating water at CSOs. LTCPs are structured around rainfall projections available at the time the LTCP is developed; one LTCP in the state has an approval date of 1995.^276^ More frequent extreme rainfall events anticipated under climate change could reduce the effectiveness of certain elements of the LTCPs, increasing the potential for CSOs. In particular, elements of LTCPs dependent on storage or infiltration of stormwater could capture a smaller fraction of certain storm events.

Impacts to WWTP operations are also a concern. Prolonged periods of low flows in CSSs can result in deposits of highly concentrated polluted sediment in sewers. The first flush from subsequent large storm events will then transport this sediment, impacting operations at the treatment plant. Climate change could result in more frequent dry periods punctuated by extreme storms that will increase these operational challenges.^72^

#### 4.2.3 Septic systems

Septic systems often serve a single residence but can also serve larger buildings such as schools in rural areas. They provide a means to treat domestic wastewater in lieu of a centralized WWTP. Traditional septic systems consist of a tank that collect solids and a leach field in which liquid waste is dispersed into the ground where the soil attenuates pathogens and nutrients through biological activity and filtration. For a septic leach field to function, there must be at least several feet of unsaturated soil between pipes emitting wastewater in the leach field and the groundwater table. Unsaturated soil refers to a soil that does not have water‐filling pore paces between soil particles. Groundwater table fluctuations are influenced by precipitation; therefore, design criteria for septic systems often reference the highest annual water table. As extreme precipitation events increase, groundwater fluctuations could exceed historical estimates of the highest annual groundwater table. In coastal areas, sea level rise can result in a gradual rise in the groundwater table that further reduces the depth of unsaturated soil between the septic system and the water table.^280^

If the unsaturated soil depth is minimal, discharged waste is not fully treated; instead, the waste can rapidly transport into the groundwater and eventually flow to nearby surface waters.^281^ Even if there is sufficient unsaturated soil depth, certain pollutants such as nitrate can move through the soil with little attenuation and contaminate the groundwater. A single poorly functioning septic system is unlikely to cause widespread pollution. However, widespread septic system failure can cause the discharge of pollutants to groundwater—older septic systems may already be discharging directly to groundwater—which can negatively impact water quality.^282^ Further, there are likely many septic systems that are currently functioning poorly due to improper design, lack of maintenance, or undersized infrastructure. Groundwater issues due to climate change will exacerbate these problems in areas with high reliance on septic systems.

Eastern Long Island offers an example of the challenges associated with septic systems. Sandy soils coupled with a high density of septic systems and little soil distance above the water table in coastal areas have led to high concentrations of nitrate in groundwater.^280^, ^283^ As this groundwater discharges to surface water, it can fuel HABs and contaminate near‐shore areas.^284^, ^285^ Increasing water tables in near‐shore areas due to sea level rise will further short‐circuit leach‐field filtering and increase nitrate loading from septic systems.^280^ During Superstorm Sandy, septic systems in Suffolk County were flooded, creating a rapid contamination pathway for untreated waste into groundwater and surface waters.^286^, ^287^

#### 4.2.4 Storm sewer systems and stormwater management

While some older urban areas in New York have combined sewers, other portions of the state have separated stormwater conveyances, often referred to as municipal separate storm sewer systems (MS4s). These MS4s include larger networks of inlets, pipes, ditches, and outfalls. Stormwater runoff transports surface contaminants from developed areas through MS4s to waterways via untreated stormwater discharges. Stormwater runoff can also cause erosion and transport sediment. Increased precipitation intensity will stress MS4s, which are typically designed for specific hydrologic parameters.^288^, ^289^

Additional stormwater management concerns include localized flooding, erosion at outfalls and culverts, and sea level rise inundation at storm drain outfalls.^278^ Climate change will affect stormwater quality, and updated permitting processes will likely be necessary pursuant to the requirements under the Community Risk and Resiliency Act as amended by the Climate Leadership and Community Protection Act (the Climate Act), similar to the recent changes made to the multisector general permit for stormwater discharges associated with industrial activity.^290^ An update to New York State's MS4 permit and associated resources is underway, but it is unclear if the updated permit language will include requirements for MS4 stormwater management programs to address a changing climate.

In urban areas, storm drains have been used for decades to convey stormwater away from populated areas toward streams and other waterways. More recently, stormwater management techniques have emphasized the use of green infrastructure, low‐impact development, and other BMPs that promote on‐site stormwater management through detention and retention. These on‐site approaches reduce the volume of stormwater entering the MS4 (or CSS) by promoting increased infiltration, which also helps to reduce stormwater pollution. There is a large body of work indicating the potential value of green infrastructure and low‐impact development to both minimize runoff volume and slow the progression of runoff into sewer systems in urban landscapes.^291^, ^292^, ^293^ However, installation of green infrastructure can require extensive landscape modification in urbanized areas, as well as a high degree of coordination among government entities.^294^ Many BMPs are designed to provide some pollutant removal and modify the quantity of stormwater. Many stormwater management BMPs use vegetative practices to support treatment, such as vegetated swales, planted riparian areas, and bioretention areas.

Stormwater management in more rural areas focuses on the implementation of BMPs. Outside of urbanized areas, stormwater management may rely more on voluntary rather than permitted activities. For agricultural areas, BMPs consist of various planting, livestock waste, fertilizer, and erosion prevention practices to limit the movement of pollutants into waterways.^295^

Stormwater conveyance infrastructure is designed based on local requirements, with a maximum capacity based on the size and slope of the storm drains. For example, the design criterion in New York City is to use the rainfall intensity‐duration values based on a storm with a 5‐year return period (e.g., 1.75 inches per hour for a 1‐h storm) to size storm drain pipes.^296^ Many communities use historical rainfall information but do not always use future rainfall projections for designing stormwater and wastewater infrastructure.^259^ Thus, as the climate changes, the effectiveness and level of service of the “gray” (i.e., built) and green infrastructure implemented to manage stormwater runoff will be reduced. In terms of green infrastructure, extreme high temperatures and changing rainfall patterns can reduce the effectiveness of these measures as plants become stressed or die.^297^ Maintaining green infrastructure benefits could require strategically shifting plant selections over time based on future climate conditions.^298^, ^299^

#### 4.2.5 Vulnerabilities

Factors influencing wastewater infrastructure vulnerability include outdated infrastructure design criteria, coastal infrastructure, aging infrastructure, and limited community adaptive capacity.

##### 4.2.5.1 Vulnerabilities due to outdated infrastructure design criteria

Runoff from small watersheds with limited storage is sensitive to changes in the intensity of short‐duration storms. Urban watersheds are often highly impervious, leading to high‐volume, high‐velocity runoff. The design criteria for the stormwater systems intended to convey runoff have not consistently been updated at the state or local levels to reflect increasing precipitation intensities.^259^

Stormwater infrastructure continues to be sized based on historical storm events despite the strong certainty that subdaily precipitation intensities will continue to increase. The 2022 Draft New York State *Stormwater Management Design Manual* ^300^ recommends using rainfall data from the National Oceanic and Atmospheric Administration (NOAA) Atlas 14^303^ or the Northeast Regional Climate Center extreme precipitation web tool^302^ to determine rainfall distribution curves for stream channel protection volume, overbank flood control, and extreme flood control design storms. Atlas 14 includes data through 2014, while the Northeast Regional Climate Center web tool uses data through 2008.^303^ These rainfall data sources for use in design storms represent more contemporary conditions than previous documents from the 1960s and 1970s, which used data extending back to the 1930s.^304^, ^305^ However, these design storm documents do not represent likely future precipitation intensities. As a result, new stormwater infrastructure designed to last for decades is, and will continue to be, undersized due to the gap between past and future conditions that will increase over time. The Water Utility Climate Alliance convened major stormwater and wastewater utilities from across the country, including from New York City, to help them overcome barriers associated with using climate projections to develop future condition design storms and criteria.^259^ Additionally, NOAA is in the process of updating the Atlas 14 precipitation frequency standard to account for future climate change projections.^306^ With a target completion date of 2027,^307^ the updated projections, referred to as Atlas 15, could support greater availability of forward‐looking design criteria for stormwater infrastructure.

While all stormwater infrastructure can convey larger pollutant loads when higher flows occur, CSSs can generate high loads with potential impacts to human health. Work is ongoing to evaluate where CSOs could disproportionately impact low‐income communities in the state. As an initial analysis, Figure 10-14 shows the locations of CSOs within New York State overlaid on the Centers for Disease Control and Prevention's Social Vulnerability Index. This index uses census variables such as poverty level, access to vehicles, and housing density to measure community‐level ability to respond or seek relief during natural disasters—in this case, high floodwater mixed with sewage. The data indicate that 119 CSOs are located within census tracts for which the Social Vulnerability Index summary ranking is in the top 10% statewide.^308^

**FIGURE 10-14:**
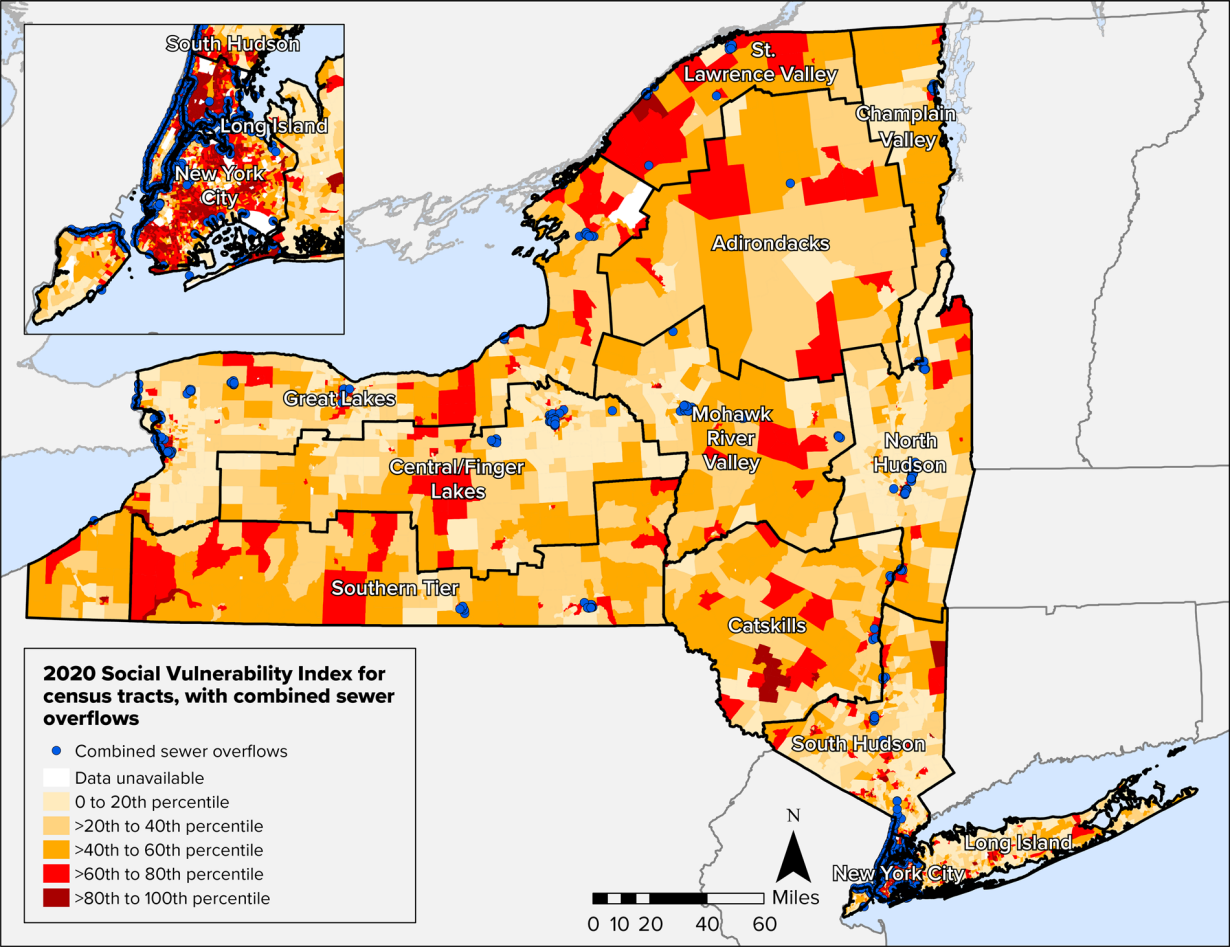
Locations of all CSO outfalls in New York State as of 2013 relative to community social vulnerability in the state. Data from New York State (2023)^308^ and Agency for Toxic Substances and Disease Registry (2024).^309^

##### 4.2.5.2 Vulnerabilities due to coastal location

Sea level rise will continue to affect drinking water and wastewater infrastructure in New York's coastal areas. WWTPs and drinking water facilities can be indirectly affected by loss of power if power generation facilities or grid infrastructure is damaged by storm events or flooding. EPA guidance addresses improvements in the reliability of power supplies,^310^ but reliability in power supply is ultimately the responsibility of individual drinking water and wastewater utilities. For power outages due to damage of distribution lines—the most common type of power outage—more isolated facilities could be more difficult to bring back online. However, for failures in the transmission network or in power generation, all facilities could be impacted.

##### 4.2.5.3 Vulnerabilities due to aging water infrastructure

Much of the water infrastructure in the state is decades old.^8^, ^11^, ^36^, ^311^, ^312^ The state's 20‐year infrastructure needs were recently estimated at approximately $44.2 billion for drinking water infrastructure and $38 billion for wastewater.^36^ Aging infrastructure is often more prone to leaks, breaks, or mechanical malfunctions that can exacerbate changes in climate hazards. For instance, a study of I&I in Vancouver, Canada, found a strong correlation between the age of each portion of the collection system and 100‐year I&I rates in the system.^313^ Coupled with increased rainfall intensities, increased I&I due to age can overwhelm both separate and CSSs, leading to uncontrolled discharge of untreated wastewater.^249^ Approximately 14,000 miles of New York State's sewers are more than 60 years old; approximately 65% of these outdated sewers experience overflow events.^12^

Older infrastructure was not designed for the present conditions in flow, precipitation intensity, or sea level. A 2022 survey of New York State's Climate Smart Communities indicated that of the 95 communities responding, 42 communities were undertaking projects to retrofit or rebuild critical infrastructure other than buildings, and more than half of those 42 communities were retrofitting or rebuilding water‐related infrastructure.^278^ Many of these projects address aging water infrastructure that experiences compounding stress from frequent storms and increased water volume.

##### 4.2.5.4 Vulnerabilities due to community adaptive capacity

The ability to adapt water infrastructure to climate hazards depends on the resources of the community that owns and operates the infrastructure. Many climate‐related impacts have an engineerable solution—such as moving a facility to a higher elevation, installing alternatives to traditional septic systems, or connecting to a microgrid—but such changes are expensive. Tribal systems are more likely to be affected by health‐based drinking water violations than non‐Tribal systems,^314^ and federal financial support for Tribal water systems, including funding for compliance, is lacking.^315^, ^316^, ^317^ Climate change will likely exacerbate such longstanding disparities for Tribal Nations and low‐income communities that lack adequate resources to fund adaptation efforts.

Drinking water and wastewater infrastructure is unique and highly dependent on the knowledge and expertise of the staff that operate these facilities. Not all of these facilities have staff who focus on long‐term planning in addition to day‐to‐day operations. For example, a survey of WWTP operators in Connecticut provided insight into how most operators perceive climate change risks.^244^ The survey found that most facilities make changes to build resilience based on actual experienced conditions that disrupted operations, rather than abstract projections of future conditions. Facilities with the resources to hire dedicated staff or consultants or partner with research organizations to focus on strategic planning and asset inventorying could have a greater likelihood of adapting based on scientific data that project future conditions.

## 5 ADAPTATIONS

A wide variety of adaptations can address the climate impacts and vulnerabilities facing New York State's water resources. Many available adaptations simultaneously address surface water and groundwater resources. This section describes adaptations under three broad categories: structural adaptations, nonstructural adaptations, and adaptations to institutions and governance.

### 5.1 Structural water resource adaptations

The structural category of adaptations focuses on approaches to creating more resilient infrastructure for potable water supply and wastewater management. This category captures asset planning and upgrades for water resources systems, as well as new technologies.

#### 5.1.1 Water supply contingency/asset planning and upgrades

Water infrastructure constantly evolves due to aging infrastructure, changes in development patterns, changes in regulations, and fiscal pressures that often encourage system consolidation. Water planners and engineers seek to add resilience and redundancy as they expand and modify water systems. In many cases, even if not specifically motivated by climate change, system upgrades reduce sensitivity to climate change. The following subsections describe some of the beneficial planning and upgrade efforts that will improve water supply infrastructure resilience.

##### 5.1.1.1 Consideration of climate resilience in asset planning

Water utilities are generally planning‐oriented. Even without the influence of climate change, water utilities must continually look ahead to plan for changing water demand, changing water quality standards, and the need to replace aging infrastructure. At times, additional planning is required. For instance, since the early 1990s, the New York State Public Health Law (Section 1125)^318^ has required public water suppliers to prepare formal contingency plans for any shortfalls in supply that could occur due to events such as bioterrorism or cyberattacks. These plans require water suppliers to identify existing and future sources of water, identify available interconnections, develop strategies to reduce water use, and develop methods for prioritizing users during any shortfalls. Similarly, the America's Water Infrastructure Act,^319^ passed in 2018, required water systems nationwide serving more than 3300 people to develop risk and resilience plans and emergency response plans. Large water systems had to complete this planning by September 2020,^320^ while smaller systems were given until the end of 2021. The America's Water Infrastructure Act focused on impacts of malevolent acts or natural hazards,^319^ but planning for these incidents should also provide some resilience for climate change in the form of new interconnections and pathways for seeking assistance in operations if climate disturbances were to occur.

##### 5.1.1.2 Water supply infrastructure upgrades

Water utilities often look to add redundancies that can enhance the reliability of their systems under any number of scenarios, including natural disaster, equipment failure, unexpected water quality issues, or climate change. For instance, several New York towns have undertaken water infrastructure upgrades that have included new interconnections among formerly separate water systems. Following the loss of power after Tropical Storm Lee, the village of Endicott's pumping systems failed^321^, ^322^ and its water mains were damaged by depressurization. Using federal recovery money, Endicott constructed a new water main that also provided an interconnection to the water system in the adjoining town of Vestal, allowing for water transfers between the two systems. This upgrade contributes to resilience in both towns, increasing their ability to respond to and recover from future climate events such as droughts and flooding.^322^

As other municipalities across the state undertake water system upgrades and plan for future replacement of infrastructure, it is important to encourage them to consider climate resilience. A study of behaviors of water treatment plant operators^244^ noted that most have sought to increase resilience but have done so largely based on storm events they have experienced themselves and not based on projections of future conditions. Entities such as the New York Rural Water Association provide free technical assistance via a professional staff of “circuit riders” who can visit facilities throughout the state, although much of the expertise offered focuses on issues such as detecting leaks, locating pipes, and testing water quality. In contrast, large system operators such as NYC DEP have staff to conduct simulations of the effects of potential future climate on water resources. The New York State Environmental Facilities Corporation helps communities pay for infrastructure projects by providing access to low‐cost capital and grants. To motivate the formal incorporation of climate resilience into project plans, the Environmental Facilities Corporation could strengthen requirements for municipalities and water systems applying for these funds.

As detailed earlier in this chapter, the treatment and distribution of potable water depends on extensive infrastructure. Adaptation to a changing climate could require modifications to some of that infrastructure. Infrastructure upgrades can be expensive, with a sizable portion of this cost passed on to water consumers. Of particular concern are increases in water rates that exceed growth in consumers’ income. These increases in water rates can result from required new investments as well as a loss of customer base in areas with declining populations. Smaller utilities are often more sensitive because they have fewer customers across whom they can spread fixed costs.^8^ As a result, considerations of possible means of building resilience to climate change must include whether a community can actually afford such upgrades.

#### 5.1.2 Wastewater infrastructure resilience planning and upgrades

This section highlights wastewater infrastructure resilience approaches involving both planning and upgrading structures.

##### 5.1.2.1 Incorporating resilience planning into facility upgrades

Wastewater treatment infrastructure has a lifespan of about 25−50 years, meaning that it must be refurbished and upgraded every several decades to remain operational. This routine replacement of aging infrastructure provides opportunities to adapt to a changing climate. Opportunities also arise in situations where extreme weather events cause damage or system failure. For example, damage to infrastructure caused by Superstorm Sandy and the remnants of Hurricane Irene led WWTP operators in the Hudson Valley and New York City metropolitan area to reevaluate facility vulnerabilities and take steps to address them—both during urgent poststorm repairs and during the more deliberate upgrades that followed.

In the next several decades, facilities in coastal areas can likely adapt by raising critical mechanical and electrical systems to higher elevations. However, by the end of the century, some facilities may be at higher risk of frequent damaging flooding, which can lead to major adaptation issues. Unlike most buildings, where the building function is not innately linked to its position in the landscape, WWTPs are tied to the wastewater conveyance infrastructure. This can make WWTPs difficult to relocate; rebuilding a plant at a new location would require reconstruction of significant conveyance infrastructure and might require pumping to get wastewater to a higher elevation. In addition, property acquisition, permitting, and construction for a new facility can take years. Therefore, the typical adaptation response for existing facilities is hardening them to higher flood risk.

Wastewater facility flooding can be addressed by methodical evaluation of flood risks.^323^ Constructing berms and floodwalls (Figure 10-15), raising equipment, and fortifying structures are typical adaptation responses to increased flood risk for these critical facilities. One indirect issue is loss of power, which can endanger many treatment processes. Many facilities have explored the possible use of microgrid power supplies. A microgrid can be separated from the larger regional power grid and supplied by a local power source.^310^ Some WWTPs already include cogeneration facilities that burn methane generated from the treatment process to create heat and electricity.^310^ New York City's North River Wastewater Treatment Plant was recently upgraded to include methane cogeneration, providing additional resilience to disruptions to its power supply.^324^

**FIGURE 10-15:**
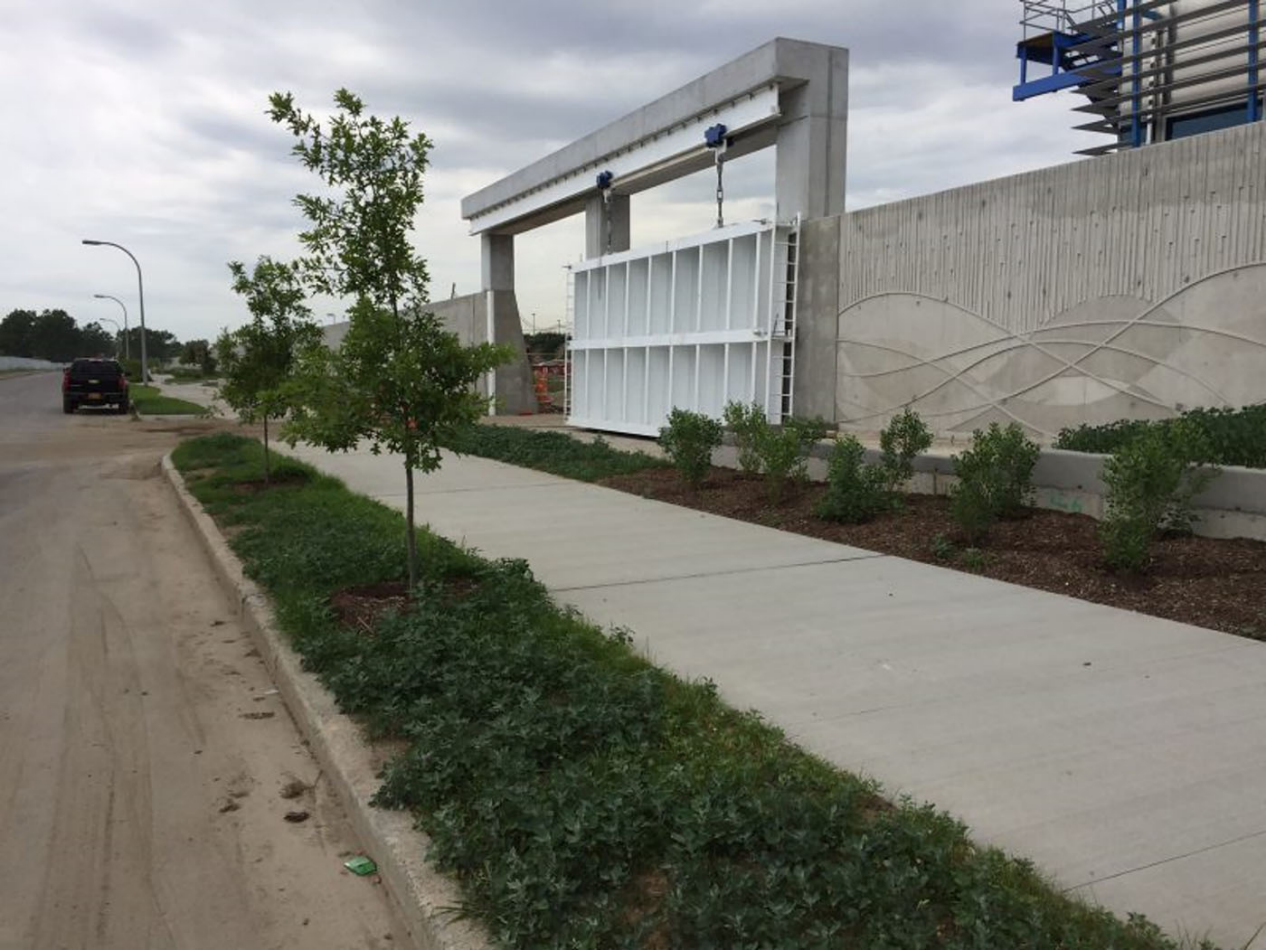
A perimeter flood protection berm and wall installed after Superstorm Sandy at the Bay Park Sewage Treatment Plant in Nassau County. Photo courtesy of Haugland Group.

To address operational challenges for wastewater treatment, utilities can work to “tighten” conveyance infrastructure to reduce I&I by disconnecting cross connections with the storm drain system and replacing or lining old pipes. Utilities can also build or expand equalization facilities to accommodate larger future inflows and prevent overflows at their plants.

##### 5.1.2.2 Minimizing CSOs now and in the future

As described in Section 4.2.2, CSOs are a primary concern across the state due to their impacts on water quality. Because CSOs occur during periods of heavy precipitation, they are likely to become more common as climate change leads to more frequent extreme weather events. This creates a strong need for effective adaptation measures.

Strategies to reduce the frequency and volume of CSOs have included rebuilding sewer networks to separate storm sewers and sanitary sewer systems and installing wet weather storage and green infrastructure to reduce stormwater volume and slow the time of concentration.^17^, ^325^ State regulators work with CSS municipalities to develop LTCPs that identify an appropriate balance of gray and green infrastructure for specific watersheds.

Infrastructure upgrades are often very expensive, especially for CSOs. Some municipalities have found that the most cost‐effective projects are opportunistic ones completed in conjunction with other necessary upgrades. For instance, Onondaga County has carried out more than 250 projects,^326^ large and small, as it has worked to abate CSOs. One particularly cost‐effective project was the separation of catch basins and the plugging of an existing, unused sanitary sewer line that was conveying nearly 11 million gallons of groundwater annually into the municipal sewer. The county was able to complete this wastewater infrastructure work during the reconstruction and repaving of Grand Avenue in the city of Syracuse, making it a low‐cost, high‐impact effort.^326^ Similarly, a major project to reconstruct Interstate 81 in Syracuse has led Onondaga County and the New York State Department of Transportation to coordinate on measures to enlarge stormwater trunk lines so they can handle the 50‐year return period storm, along with other more routine stormwater management measures. Well‐coordinated planning can ensure that any new work helps to minimize CSOs and manage urban stormwater both in the near term and in a future with heavier storms.

One barrier to addressing increased CSOs in the future is that many LTCPs are structured around the outdated rainfall statistics that were in place when the plans were developed. In many cases, changing precipitation would have a considerable effect on the runoff generated (refer to Section 4.2.2). For example, a modeling study of projected changes to stormwater generation after the installation of porous pavement in Buffalo found sizable differences in runoff volume depending on the intensity and duration of storms.^327^ These results suggest that communities can benefit from incorporating climate change projections in LTCPs.^327^ However, EPA's CSO Control Policy does not explicitly require communities to account for climate change or update LTCPs based on future weather patterns.^328^ Revisions to federal and state standards would be needed to require municipalities to incorporate climate change into LTCPs for CSO management.

Some researchers and communities have begun to look at the application of new technologies for managing CSOs. One promising technology is the use of real‐time controls on combined sewer outfalls. This technology relies on sensors for rainfall and CSO discharges to enable proactive management of control structures to limit CSOs. A research team that conducted a case study in the province of Quebec, Canada, found that real‐time controls for CSSs, used in conjunction with green infrastructure and stormwater improvements, could achieve a 98% reduction in CSOs.^329^ In 2012, New York City initiated a pilot program using remote sensors to measure and monitor combined sewer discharges at five outfall locations. The purpose of the pilot was to determine if remote sensors could produce accurate information on the occurrence, duration, and volumes of CSO events and if the information was reliable enough to be used in real time.^330^ While there were many benefits from the study, the data produced were not sufficiently accurate for near‐real‐time notification on a permanent basis. While real‐time controls can help reduce CSOs, the New York City pilot highlights some of the practical challenges with using sensor technology in difficult‐to‐reach locations exposed to harsh environmental conditions.

##### 5.1.2.3 Exploring new technologies for on‐site waste treatment

As noted earlier, more frequent high‐intensity precipitation events or rising groundwater levels can lead to more frequent saturation of septic leach fields, thus short‐circuiting the treatment process and resulting in the export of nutrient and pathogen waste into groundwater and surface water supplies. However, besides traditional septic systems, there are now numerous modern on‐site treatment technologies that do not rely on leach fields for treatment and are less sensitive to rising groundwater or intense precipitation. These on‐site treatment technologies use processes such as filtration or aeration to efficiently remove pollutants in a compact unit suitable for residential properties.^331^

Implementation of new on‐site treatment technologies is already being carried out on Long Island. The impacts of Superstorm Sandy, coupled with chronic high nitrate loading in groundwater and surface waters that have degraded water quality, resulted in a 2019 policy initiative in Suffolk County to replace traditional septic systems with central sewers and require new construction to use innovative designs that more actively treat nitrate.^332^, ^333^ Nassau County's Septic Environmental Program to Improve Cleanliness offers grants up to $20,000 to homeowners and small businesses that install nitrogen‐reducing septic systems.^334^ Technologies approved by the county's program to increase nitrogen removal include an activated sludge treatment process and flow systems with sequenced anerobic and aerobic reactors.^334^ In 2022, Governor Kathy Hochul announced the availability of an additional $30 million through the State Septic Replacement program to help remove and replace aging and failing systems.^335^

On freshwater lakes throughout New York, there is a similar need for new on‐site wastewater treatment technologies that remove phosphorus. As lakefronts become more densely packed with residences, many of which are used year‐round instead of seasonally, phosphorus levels in soils may be approaching a saturation point, and over time additional phosphorus could move from leach fields into surface waters.^336^, ^337^ While centralized WWTPs successfully use several technologies for phosphorus removal, these often require extensive user maintenance, making them an inappropriate choice for residential septic systems.^338^ Phosphorus removal technologies appropriate for residential on‐site treatment remain in the early stages of development.

##### 5.1.2.4 Tribal wastewater infrastructure adaptations

The Suffolk County septic system policy does not apply to Tribal territories on Long Island, including the Shinnecock and Unkechaug Nations, and Tribal citizens living on Tribal territories are not eligible for the subsidy programs provided through the new policy.^339^, ^340^ There are additional environmental justice and septic affordability concerns for overburdened communities, as the average cost of system replacement (not including costs associated with engineering and design) can be more than $25,000,^339^ which is often more than a family's available home improvement funds. Some Tribal Nations, such as Tuscarora Nation and Shinnecock Nation, have partnered with the Indian Health Service to receive upgraded septic systems and drinking water wells.^232^, ^341^, ^342^

The Seneca Nation of Indians is working to address infrastructure deterioration at the Thomas Indian Wastewater Treatment Plant.^343^ Additionally, the *Saint Regis Mohawk 2013 Climate Change Adaptation Plan* underscored the Tribe's completion of its sewer line systems through the southern portion of Akwesasne.^57^ However, only one district in the northern portion of the territory had been completed, highlighting the persistent challenges Tribal Nations face in meeting the necessary capital resource requirements to provide sanitation access for all.

### 5.2 Nonstructural water adaptations

Climate resilience for water resources also involves acknowledging the role of nonstructural factors such as policies and standards, planning, education, and behavior change. This section highlights nonstructural water adaptations under these categories.

#### 5.2.1 Policies and standards

Policies and standards play a foundational role in establishing the technical design criteria and other requirements needed to ensure the development and implementation of climate‐resilient infrastructure and practices. As discussed in Section 4.1.1, the 10 State Standards encourage water treatment facilities to have auxiliary power sufficient to meet average daily demand and that all water supply facilities and water treatment processes be protected to at least the 100‐year flood or maximum flood of record.^217^ Policies and standards that have not been updated to address climate considerations, such as the CSO LTCP requirements discussed in Section 5.1.2.2, could be updated accordingly to promote adaptation and resilience.

Statewide policies and standards can promote more consistent consideration of climate impacts by water utilities, decisionmakers, and other stakeholders. Where statewide guidance is not fully updated, acknowledging climate considerations is a start. NYSDEC released the latest draft of the state's *Stormwater Management Design Manual* ^300^ in May 2022. This manual provides design standards for stormwater management practices with the goal of mitigating the impacts of urban stormwater runoff. This version of the manual acknowledges the impacts of climate change and states that future versions of the manual will provide guidance on climate‐resilient stormwater management practice design. For the time being, the manual points readers to other NYSDEC guidance documents to help them design climate resilience measures, including documents on flood risk management and tidal wetland considerations.

In addition to using statewide design guidelines, municipalities can also review and update local design standards and policies for capital projects to better address climate impacts to water resources. For example, the New York City Mayor's Office of Climate and Environmental Justice released *Climate Resiliency Design Guidelines* (Version 4.1) in May 2022.^344^ These city guidelines go beyond existing building codes and standards to address climate impacts in the design of city capital projects, including impacts to water resources because of increasing precipitation.^344^ For enhanced on‐site stormwater management systems, the guidelines focus on improved stormwater management at the neighborhood scale, the sewershed scale, and the street/site scale by encouraging project designers to use techniques that increase the capacity of detention/retention systems. The guidelines recommend that designers consider a 10−40% increased capacity from base design; incorporate stormwater reuse practices; decrease impervious surface cover to promote on‐site infiltration; and use vegetated stormwater management practices such as rain gardens, vegetated swales, tree planting, and green roofs (i.e., green infrastructure).

#### 5.2.2 Planning

Clean water plans represent a notable adaptation opportunity for New York State's water resources. NYSDEC provides oversight and guidance on numerous types of clean water plans for surface waters in the state, including nine‐element plans, total maximum daily load plans, and drinking water source protection program plans.^345^ While the purpose and requirements for these plans vary, they generally document pollution sources, set pollutant reduction goals, and identify strategies that can improve water quality. Communities or stakeholder groups that want to implement actions identified in a plan, including climate adaptations tailored to a specific watershed, are eligible to access federal and state funding.^345^ Access to funding is particularly important for controlling nonpoint pollution sources that are precipitation‐driven and rely on voluntary action to implement management practices. Existing clean water plans, however, might not fully address climate impacts to the watershed or water body of concern. Older clean water plans could require updates to reflect new data and information related to climate impacts that are specific to New York State and unique land uses and hydrologic characteristics of a given watershed.

#### 5.2.3 Education and outreach and behavior change

Other important nonstructural adaptation approaches are those that embrace stakeholder education and outreach, especially initiatives that focus on behavior change. Stakeholders can include individual residents, business owners, and government agencies. Behavior changes that are key to addressing climate impacts to water resources include water demand reduction, water reuse, and improved on‐site stormwater management. Improved on‐site stormwater management can include voluntary decisions to use on‐site techniques (e.g., green infrastructure, stormwater capture and reuse) that promote infiltration and reduce the volume entering sewer infrastructure, although municipal codes and ordinances often require these approaches. Water demand reduction and water reuse are other important nonstructural adaptations that can reduce both the volume of water treated for use as potable water and the volume requiring treatment as wastewater. This section focuses on demand reduction and water reuse as education and outreach‐driven climate‐resilient behavior changes.

##### 5.2.3.1 Demand reduction

Within the system of treating, distributing, and using potable water, there remain numerous opportunities for reducing water losses or nonessential water uses. Taken together, these management approaches are broadly referred to as demand reduction. Demand reduction can increase resilience to drought while also reducing operational costs and energy use.

New York City has worked to reduce demand since the 1980s. The city reports that per capita demand has declined from 213 gallons per day in 1979 to only 115 gallons per day in 2017.^346^ Thus, while the city's population has grown by more than 1 million people since the early 1990s,^346^ demand has dropped. The city made initial gains by promoting more widespread adoption of metering, reducing pipe leaks, and emphasizing the importance of increased conservation. NYC DEP has continued to increase water efficiency, launching several new programs introduced under the city's 2013 and 2018 Water Demand Management Plans. For instance, NYC DEP partnered with other New York City agencies to install push buttons on spray showers in park play areas, which has saved 1.1 million gallons per day as of May 2018; upgrade bathroom fixtures in 700 school facilities, which has saved 4.71 million gallons per day as of 2022; and identify nonessential uses of potable water to sustain park water bodies, which has saved 1.05−1.25 million gallons per day as of 2022.^346^ NYC DEP also promoted “water challenges” that encouraged hospitals, restaurants, and hotels to undergo water audits and follow through to make measurable reductions in water use.^346^

Another program in New York City, the “Wait…” initiative, relies on a cell phone app to notify program participants when heavy precipitation is forecast and the risk of CSOs is greatest. The goal of the initiative is to induce water users to avoid household water use during heavy rains to reduce the likelihood that the combination of stormwater and wastewater will exceed the capacity of the CSS.^347^

Residential irrigation for watering lawns and gardens has been identified as a large driver of summertime water use in suburban areas.^65^ Using data from peak summer and typical winter days in 2007, the Suffolk County Water Authority noted how peak demand shifted from about 100,000 gallons per minute in winter to nearly 500,000 gallons per minute in summer.^348^ To address this surge in summer water use due to irrigation, suburban municipalities have used targeted techniques to reduce demand. The Roslyn Water District on Long Island, for example, has promoted a “Save Two Minutes” program, asking residents who use automatic irrigation systems for lawn watering to cut back their water use by 2 min per zone.^349^ Based on an average duration of 20 min of irrigation per zone, this reduction should reduce water use by 10%. Additionally, modern irrigation systems can incorporate rain sensors or soil moisture sensors such that irrigation is not used when there is already sufficient water for plant growth.^350^

For homeowners accustomed to green lawns and thriving gardens, water use could temporarily increase during drought periods until drought becomes severe enough that homeowners shift their behavior. However, behavioral changes sometimes require government mandates, which can reinforce existing inequities on already‐burdened communities.^351^ In the context of a severe, multiyear drought, water conservation mandates in California led to 25% declines in use.^352^

##### 5.2.3.2 Water reuse

Water reuse is gaining national attention as a strategy to improve the security, sustainability, and resilience of water resources across the country.^353^ This approach involves collecting wastewater, stormwater, or gray water (nonsewage wastewater from sinks, baths, and laundry) and treating the collected water to a level that is acceptable for another use, such as irrigation, power‐washing pervious surfaces, or flushing toilets.^354^ Stormwater and rainwater capture (also referred to as stormwater harvesting) commonly occurs through the use of rain barrels and cisterns. Captured water is typically used on site, often for irrigation purposes. The acceptance of water reuse as a practice is often related to stakeholder education and perceptions.^353^

In New York State, water reuse is gradually becoming more common, driven by specific watershed needs. For example, the Long Island Nitrogen Action Plan Water Reuse Advisory Workgroup has identified the opportunity to use treated effluent from centralized wastewater treatment facilities for golf course irrigation.^355^ The benefits of doing so include reducing demand on Long Island's sole source aquifer and reducing fertilizer use at golf courses, which could in turn lower the amount of nitrogen pollution entering Long Island's surface waters and groundwater as a result of runoff from golf courses (a problem likely to be exacerbated as extreme precipitation events become more frequent).

A recent study on the potential for water reuse in the Hudson River Valley^356^ demonstrates that water reuse is not a commonly occurring practice but has the potential to improve climate resilience for three sectors: food and agriculture; wineries, breweries, and cideries; and recreation (e.g., using treated effluent to support snowmaking). Determining why water reuse is not a common practice could be beneficial. It is possible that increased outreach could result in increased adoption of water reuse practices.

### 5.3 Institutional and governance adaptations

Many water resource adaptations rely on changes to institutions and the governance process. This section explores some adaptations involving management approaches.

#### 5.3.1 Continued monitoring to better recognize climate‐driven trends

While monitoring of water bodies and treated water already takes place in New York, the state could benefit from more closely aligning these efforts to changes in climatic conditions. A substantial data gap exists in understanding how cumulative and interactive changes in climate variables such as temperature, precipitation, and humidity^357^ affect ecosystems and watersheds,^358^ and by extension, source waters used for drinking water. Proactive management of the state's water quality should include continued monitoring of source water and treated drinking water; more detailed (i.e., higher temporal and spatial resolution) tracking of key water quality parameters in problem areas such as tributaries with recurrent high‐turbidity events; and tracking of high‐frequency climatic and hydrological data.^357^ The majority of lake water quality data collected by NYSDEC, either from the volunteer‐based CSLAP or from the Lake Classification and Inventory Program conducted by NYSDEC staff, are snapshot data. These data come from sampling events that occur biweekly (or even less frequently) between May and October. There is no official monitoring of lake ice cover, under‐ice lake temperature, or under‐ice water quality—variables that are measured in some other states, including Vermont and Minnesota. Most high‐frequency lake data buoys, which collect weather and water quality data simultaneously, are owned by colleges, universities, and research institutions but are operated and maintained by individual principal investigators and are dependent on grant funding and local donors rather than being funded as public infrastructure to be maintained and replaced in perpetuity.

#### 5.3.2 Using flexibility of existing governance structures to respond to climate change

The laws, compacts, and regulatory instruments governing access to water in New York State are not often at the forefront of policy and management decisions, largely because the state sits in a region that is infrequently water‐constrained. Typically, these laws and resource‐sharing arrangements are revisited only when there are droughts or dramatic shifts in water demand. For instance, increased stress on water resources in the western United States led, in part, to the passage of the Great Lakes Compact in 2008^114^ to prevent water transfers out of the Great Lakes watershed. Similarly, the possibility of hydraulic fracturing in New York State in 2010 led to the development of new standards for minimum flows in streams and rivers.^359^

As discussed earlier in this chapter, changing climatic conditions could lead to increased demand for access to water resources at the local and regional scale, while also affecting the quantity and quality of available water resources. As such, climate change could result in the need for new regulatory arrangements to help protect the resources and manage competing water demands. Instead of waiting until water stress conditions arise, it is useful to plan ahead and create well‐developed regulations and agreements that balance economic, ecological, and social impacts.

Section 2.1.4 introduces several in‐state and regional entities that have formal cross‐boundary governance structures or other unique water management responsibilities, including the IJC, the DRBC and SRBC, and the HRBRRD. Most of these institutions have taken steps to address climate change in their management plans and agreements, including the following examples:

- **International Joint Commission**. The 2012 amendment to the Great Lakes Water Quality Agreement,^360^ which was shaped by reports and recommendation of the IJC, included a new Annex 9 on “Climate Change Impacts” that committed the United States and Canada to “coordinating efforts to identify, quantify, understand, and predict the climate change impacts on the quality of the Waters of the Great Lakes, and sharing information that Great Lakes resource managers need to proactively address these impacts.” In 2015, the IJC established the Great Lakes–St. Lawrence River Adaptive Management Committee^361^ to evaluate how the regulation of water levels and flows is being impacted by changing conditions (in particular, climate change) and what modifications may be needed in response. The International Watershed Initiative is another recent IJC program that applies an ecosystem focus and engages local communities to help develop solutions for managing binational watersheds.^362^ Both the Great Lakes–St. Lawrence River Adaptive Management Committee and the International Watershed Initiative will help the IJC respond more quickly and with better public support as climate change disrupts weather patterns and affects water levels and flows in U.S.–Canada transboundary waters.
- **Susquehanna River Basin Commission**. The SRBC, in its comprehensive plan for 2021–2041, stated that “The Commission regards climate change as an existential threat to the [Susquehanna River] Basin's water resources in terms of more extreme floods and droughts, vulnerability to reliable water supplies, and exacerbation of critical issues already facing the Basin such as water quality impairments, stormwater runoff, and the need to plan for future water demands.”^363^ The SRBC's management plan directly integrates climate change into its four priority areas: water supply, water quality, flooding and drought, and watershed management. For instance, within the flooding and drought priority area, there is an emphasis on expanding the use of climate change projections, increasing the use of data analytics and technology for drought and flood forecasting and early warning, and identifying new water supply storage and backup sources.
- **Delaware River Basin Commission**. The DRBC's *Water Resources Program* planning document for 2023–2025 states that projected impacts of climate change on the Delaware River Basin—including greater extremes in precipitation, higher temperatures, sea level rise, and increased flooding—“will affect water supply and water quality—critical components of water security in the [basin].”^364^ The DRBC staff is using hydrodynamic and hydrologic models to study the impacts of sea level rise on salinity transport and to develop a range of scenarios for evaluating flow management programs. The goal is to “provide scientifically‐based information to the Commissioners and Basin stakeholders to support development and implementation of water resource management strategies in the Basin that increase the resiliency of our water resources to anticipated changes in climate and rising sea levels, as well as support improved adaptation planning.”^364^
- **Hudson River–Black River Regulating District**. The dams controlled by the HRBRRD allow substantial flexibility in responding to extreme weather events. Although climate change has not been a pressing concern of the HRBRRD until this point, its leadership recognizes that climate change will likely impact its operations in coming years (Callaghan J, Executive Director, Hudson River–Black River Regulating District [2022, April 29, Personal communication]). Still, the models and protocols the HRBRRD team uses for managing levels and flows in the system of rivers and dams under its control are expected to be sufficient for handling more extreme conditions resulting from climate change in the near term. The HRBRRD's engineers may need to make adjustments more actively in response to climate‐driven fluctuations in weather patterns and precipitation, but the district does not anticipate having to improve its physical infrastructure or adjust regulatory protocols before the Conklingville Dam undergoes a review for the renewal of its Federal Energy Regulatory Commission license in 2040 (Callaghan J, Executive Director, Hudson River–Black River Regulating District [2022, April 29, Personal communication]). Releases from the Great Sacandaga Lake reservoir through the Conklingville Dam can be used to control the position of the Hudson salt front. This is especially important during the summer months, when Conklingville Dam releases can help push the salt front further south and protect the drinking water inlets for downstream communities such as Poughkeepsie. The HRBRRD is planning to join several downstream municipalities in a study to be sponsored by NYSDEC on how best to manage the salt front in the coming years as climate change threatens to cause more extreme fluctuations of the salt front's upstream limit (Callaghan J, Executive Director, Hudson River–Black River Regulating District [2022, April 29, Personal communication]).

#### 5.3.3 Adapting the permitting process to better manage water withdrawals

Any shifts in water supply (due to climate) or demand (due partly to climate but also to population growth, economic development, etc.) could result in competing demands for finite water in certain water bodies. The portions of the state within the bounds of the Susquehanna and Delaware River Basins have established commissions with full‐time technical staff to coordinate and permit withdrawals and help manage diversions during periods of low flow. Other regions of the state also require reporting of withdrawals and permitting of new withdrawals with an average capacity greater than 100,000 gallons per day in any 30‐day period by NYSDEC^365^, ^366^ for review by the Division of Water Management. Permitting requires that a licensed engineer complete a report that both details available supply and considers other reported demands on the same water body. This permitting requirement has been in existence since 2012, but the state has offered limited guidance on how to estimate supply, and there is no mechanism to assess whether all withdrawals are actually being reported—especially those that might just be temporary during dry periods. Understanding availability could be particularly difficult for rivers and streams that are ungaged, meaning they lack current or historical information on flow fluctuations. Furthermore, there is often no mechanism—besides the courts—for arbitrating disagreements regarding flow availability and addressing conflicts in their early stages. It may be useful to consider developing more standard technical tools and water models for transparently evaluating and communicating water availability in large and small water bodies across New York.

#### 5.3.4 Broadening perspectives and expanding involvement in water governance

Communities across New York have already started the process of envisioning new laws and governance arrangements to protect future water resources. In November 2021, New York State citizens voted to include an amendment to the state constitution noting that “Each person shall have a right to clean air and water, and a healthful environment.”^367^ Often referred to as “green amendments,” these types of legislative interventions have grown more common in recent years in response to climate change and related water crises.^368^, ^369^

Such thinking may be novel for the legislative process, but it is not new to New York. Tribal Nations and Indigenous Peoples in New York have knowledge systems that have emerged from thousands of years of engagement with their local landscapes. Indigenous Peoples’ science and knowledge offer a clear vision for holistic, sustainable water development, often different from the narrower regulations of state and federal laws. For instance, the Haudenosaunee Environmental Task Force “Position on Climate Change” has articulated a path toward sustainability embedded within The Dish With One Spoon wampum agreement:

“Haudenosaunee Indigenous knowledge of The Dish With One Spoon spells out our relation to the natural world—Mother Earth. The Great Law of Peace given to us centuries ago informs us that everything that we need as human beings is gifted to us by Creation. That we were to take only what we needed. The Dish is a metaphor for those teachings of harmony with Mother Earth and the beaver tail soup includes all those resources placed on a menu for shared consumption based on equality.”^370^

Teachings from the Haudenosaunee also offer ways to negotiate healthy relationships with Indigenous and non‐Indigenous people. These teachings include the Kayanerenkó:wa (Great Law) and Kaswentha (Two‐Row). The Kayanerenkó:wa is the unifying teaching among the six Haudenosaunee communities (Tuscarora, Seneca, Cayuga, Onondaga, Oneida, and Mohawk) and is based on three foundational principles: peace, power, and a good mind.^371^ These foundational principles helped the Haudenosaunee Nations negotiate healthy relationships with each other through a deep understanding that peace is not the absence of conflict but an active process of negotiation and dialogue. The Kaswentha is an important agreement that was established between the Haudenosaunee and Dutch to travel down the river of life together while respecting each other's autonomy in perpetuity. It is sometimes visually depicted as a belt with two parallel lines of purple wampum. The two lines represent the two separate but equal paths of two entities, with neither attempting to change the trajectory of the other.^372^ The Kaswentha emphasizes the importance of diversity in thought but requires respect and the removal of power hierarchies. These teachings are applicable to relationships beyond governing bodies and can extend to interactions and negotiations among individuals, businesses, or community groups.

Indigenous Peoples have historically been excluded from water governance decision‐making.^373^ However, the federal government has recognized the importance of valuing Indigenous Traditional Ecological Knowledge in environmental decision‐making, including decisions about water resources.^374^ An example is the appointment of the first Indigenous commissioner to the IJC, Henry Lickers, in 2019. Commissioner Lickers's involvement has led to the incorporation of Indigenous science and community voices in the IJC's water resource governance processes.^375^

## 6 LOOKING AHEAD

This section looks at opportunities for positive change that can grow out of climate adaptation efforts and identifies emerging topics and research needs in the water resources sector. The section concludes by summarizing the major findings and recommendations presented in the chapter.

### 6.1 Opportunities for positive change

New York's abundant water resources, managed and delivered through an extensive network of public and private infrastructure, are essential to every aspect of social, environmental, and economic wellbeing in the state. Impacts of climate change—including rising temperatures, sea level rise, increased precipitation, and drought—can exacerbate existing challenges with aging infrastructure, increasing water demand, and infrastructure sustainability. However, initial suggestions of the impacts of climate change in the form of extreme weather events (e.g., Superstorm Sandy) have already prompted the state to plan, make new investments, and adopt innovative practices to enhance the resilience of its resource management approaches and infrastructure.

The importance of and willingness to undertake these investments is reflected in recent state and federal actions and initiatives. For example, in 2021, New York voters overwhelmingly voted to create a constitutional right to clean air and water and a healthful environment. This “Green Amendment” was enshrined as Article I, section 19 of the New York Bill of Rights.^367^ More recently, in March 2023, the first United Nations water conference in nearly 50 years was held at United Nations Headquarters in New York City, drawing stakeholders from around the world to address and accelerate solutions to challenges such as ensuring equitable and affordable access to clean water in the face of a rapidly changing climate.^376^

Many communities may previously have been unable or unwilling to undertake asset management and long‐term planning efforts due to resource constraints, limited technical capacity and knowledge, and other barriers. If such planning is necessitated by or funded in response to climate threats, it can generate substantial and long‐lasting financial, operational, and more importantly, public health benefits for utilities and ratepayers, in addition to extending the life of key infrastructure.

Preparing for and responding to climate impacts can also prompt investments that may have otherwise been further delayed (thereby prolonging the impacts of environmental and other threats caused by failing or outdated infrastructure), and may encourage innovative, cooperative solutions to what were previously perceived as local problems. For example, cooperation between water utilities, consideration of watershed‐scale solutions to problems that could provide downstream benefits, and use of natural infrastructure solutions in place of gray infrastructure carry benefits far beyond climate resilience.

In a 2012 report, the American Water Works Association estimated that the 25‐year need for addressing new and long‐standing infrastructure needs to maintain and meet service demand would cost more than $1 trillion nationwide.^377^ Local governments and utilities must navigate a complex array of funding options.^378^ Recent federal and state statutory and funding initiatives offer unprecedented opportunities for local investment in water resources and infrastructure. The 2021 Infrastructure Investment and Jobs Act provides historic levels of funding for water infrastructure, including for infrastructure resilience against climate and extreme weather impacts. The Inflation Reduction Act of 2022 provides substantial renewable energy incentives and investments in other climate adaptation and mitigation measures. Both acts place a significant emphasis on or require funding to be directed at low‐income and underserved communities and provide technical assistance and other support to help communities identify eligible water resource and infrastructure needs, design sustainable projects, access available funding, and see the project through to completion. This assistance is intended to help these communities and utilities overcome long‐standing barriers to accessing the myriad funding sources available. Such barriers include limited local capacity and knowledge to pursue funding, lack of an identified pipeline of projects and data to justify the need for the investment, inability to meet eligibility requirements, inability to take on debt or meet other financial qualifications, and competing local needs.^379^

### 6.2 Emerging topics and research needs

As knowledge of the impacts of climate change on water resources increases, so does recognition of the many ways in which water resource impacts can affect other sectors, and vice versa. Compounding challenges have also become more apparent. These challenges merit further attention.

One type of compounding challenge occurs when concurrent failures exacerbate each other. For instance, coastal storms that lead to flooding can also lead to power outages. These power outages make it more difficult to bring flooded infrastructure—such as a water treatment plant—back online. Additionally, power outages or disruptions in natural gas service can make it difficult for homeowners to boil water if their potable water is contaminated.

Similarly, the water affordability challenges discussed in Section 5.1.1.2 could coincide with other affordability challenges spurred by climate change. For example, at the same time, a changing climate leads water utilities to raise rates to pay for infrastructure upgrades, some New Yorkers may also find themselves paying higher rates for electricity and natural gas or purchasing more electricity to cool their homes in hotter weather.^72^

Responses to challenges can also exacerbate those challenges. For instance, during a drought, if an effective plan for reducing demand is not in place, dry conditions can lead to increases in demand. Residents might increase the amount of water they apply to their lawns and gardens, and some could shift away from certain water supplies (like shallow wells) to municipal, piped water. Thus, when water availability is lowest, demand could shift higher, further stressing already low water levels. Unexpected changes in demand can occur with shared sources of water that span large geographic areas, such as long rivers. A water user in the headwaters could increase their use without communicating the change to downstream water users. Under normal conditions, there may never be any need to communicate, leading to a lack of awareness of demand competition until extreme conditions actually occur. In this sense, climate change raises the need for increased coordination, such as the governance efforts discussed in Section 5.3.

Climate change also intersects with other emerging nonclimate challenges. One example of particular concern is the widespread detection of PFAS. Consumer and industrial products containing PFAS have been used for decades, ranging from firefighting foams to stain‐resistant coatings for fabrics. As analytical techniques for measuring PFAS evolve and the number of measurements being taken in the environment increases, there is an increased understanding of how widespread these chemicals are—for example, in contaminated groundwater.^380^ While PFAS contamination is an issue without climate change, a need to move away from existing contaminated water supplies could reduce flexibility in accessing water during droughts exacerbated by climate change.

The Technical Workgroup identified the following additional issues during the development of this chapter that could benefit from further investigation. Specifically, the Technical Workgroup identified the following needs:

- More systematically collected, localized data on water bodies. Limited resources for surveys and monitoring means that data often depend on volunteer monitoring, which can introduce sampling bias.
- More data on water use.
- Modeling of hydrologic impacts, which are not always a direct translation of precipitation and temperature and are difficult to construe without information on human interventions such as dams, withdrawals, and inflows.
- Improved groundwater modeling, including more precise location of the freshwater/saltwater interface and analysis of the long‐term sustainability of the groundwater supplies in particularly vulnerable areas such as Long Island's barrier islands.^381^
- Modeling of the effects of increasing sea level on the salt front for the Hudson River.
- Projections of changes in lake ice cover and Great Lakes water levels with more certainty.
- An understanding of how climate change will affect the 100‐year flood standard and PMF standard for dam safety—an active area of research and discussion within the dam safety community, with some states already proactively adjusting their PMF estimates.
- Assessments of the effectiveness of green infrastructure during extreme precipitation events across diverse New York localities.
- More research on potential interactions between climate change and current and potential water contaminants.

### 6.3 Conclusions

New York State's abundant water resources have historically provided sufficient potable supply to meet growing and changing demand, and they are critical to the health of the state's residents, ecosystems, and economy. Nearly 14.5 million New Yorkers receive water from public drinking water systems using surface water sources such as lakes, reservoirs, and rivers,^382^ and nearly 7 million receive water from public water supplies or private wells that tap groundwater.^382^, ^383^ (These numbers sum to more than the total population of the state because some people receive water from more than one system when workplaces, schools, etc., are counted.) Water treatment, distribution, and conveyance depend on an extensive infrastructure network that must be continually maintained, repaired, and replaced to protect public health and the environment.

The state's water resources and infrastructure are already affected by climate change. Rising sea levels threaten infrastructure and water resources in coastal areas, and they can have broader effects on estuarial rivers. Increases or decreases in precipitation can affect average streamflow and availability of water supplies, particularly for irrigation. Increasing temperatures and runoff can negatively affect water quality and place stress on wastewater treatment facilities. Heavy precipitation events can lead to CSOs, damage to infrastructure (including dams), and interruptions in facility operation.

Preparing for and adapting to the impacts of climate change on water resources and infrastructure largely falls under the responsibility of local governments. Much of the state's water infrastructure is aging and was not designed to operate under future anticipated climate conditions. Local governments require robust technical and financial resources to address climate change impacts. Understanding and accessing the funding available for such investments and identifying and justifying needed investments has long been a challenge for many communities, particularly smaller and historically underserved communities. Needed water resource and infrastructure investments also compete with other critical community public health, education, emergency response, and other funding needs. More broadly, underserved and overburdened communities, Tribal Nations, and other entities are particularly vulnerable to negative climate impacts, yet often lack an active voice or legal authority in decisions that affect their needs and daily lives.

Despite these challenges, communities have begun investing in enhanced water resources and infrastructure planning and innovation to improve the resilience of the state's infrastructure. State and federal legislation has made unprecedented amounts of funding and technical assistance available to communities that have historically been underfunded and marginalized. Additionally, ongoing studies and research efforts to project and manage the impacts of storm surge, sea level rise, and other climate hazards on New York will provide better data with which to make forward‐looking decisions and investments. Existing projections also indicate that the state's water resources may be resilient to or less affected by the impacts of climate change relative to other sectors. However, there is a considerable need for streamflow, recharge, and other data, as well as for standardization and harmonization of existing and future data to make it easier for local communities to access and use for decision‐making.

## 7 TRACEABLE ACCOUNTS

Traceable accounts examine each key finding in depth. They provide citations that support each assertion and present the authors’ assessment of confidence in each finding.

### 7.1 Key Finding 1

**Water bodies and groundwater under the direct influence of sea level are already being affected by climate change and will face greater risks in the future**. Groundwater wells in coastal areas will be subject to saltwater intrusion from sea level rise. Rising seas will also push the saltwater boundary on estuarial rivers such as the Hudson River farther upstream during drought periods, potentially affecting freshwater use and withdrawals. Detailed modeling of saltwater intrusion and estuary salt concentrations will lead to a better understanding of likely impacts.

#### 7.1.1 Description of evidence base

Climate change impacts at the freshwater/saltwater interface are more certain than climate change impacts on inland freshwater sources. Changes in long‐term mean regional precipitation patterns can be difficult to predict due to the multiple factors controlling precipitation generation.^82^, ^384^ While annual total precipitation is projected to increase, some seasons may be drier, particularly when increased evapotranspiration is taken into account.^72^ However, sea level rise has a well‐defined connection to physical processes affected by increasing temperature (i.e., melting of polar ice and thermal expansion of the ocean).

Saltwater intrusion, mainly due to overpumping of well water, has been documented for decades in numerous areas along the U.S. East Coast, including New Jersey, Florida, South Carolina, and Georgia.^191^ USGS evaluated the extent of existing saltwater intrusion along the eastern seaboard, including Long Island, using historical records dating back to 1903.^192^ This study found chloride concentrations greater than 250 milligrams per liter in wells of the Magothy aquifer along the southern barrier islands in central Long Island and in southern shoreline areas closer to New York City.

Observations of the salt front on the Hudson River have been collected for decades, with the inland movement of the salt front noted during past periods of severe drought.^105^ However, this work focused most extensively on a drought in the 1990s and has not considered changes in sea level.

#### 7.1.2 New information and remaining uncertainties

There are no known analyses that simulate the effects of increasing sea level on the salt front for the Hudson River, although such analyses in Spain^109^ and the Chesapeake Bay^108^ have shown upriver shifts in the salt front. Additionally, there is both anecdotal information and preliminary modeling^111^ that indicates the Delaware River (another regional estuary) could see its salt front move at least several miles upriver due to sea level rise. There are studies on the Hudson River that investigate how changes in channel bathymetry due to dredging may change salinity and threaten water supplies^385^; such models could presumably be adapted to investigate changing sea level.

While saltwater intrusion has long been a concern, older studies mainly considered its cause to be groundwater depletion and did not directly evaluate the role of changes in sea level. Studies that do consider the role of sea level rise highlight differing predictions of the extent of saltwater intrusion depending on the modeling assumptions.^386^, ^387^, ^388^ USGS is in the process of conducting a study on changing groundwater on Long Island.^185^ This new study will include geophysical mapping of the saltwater interface and provide an opportunity to assess whether the position of the interface has changed over time. USGS's study will also include numerical modeling to simulate possible future changes.

A formal salt front migration group, which includes NYSDEC, NYSDOH, USGS, Cary Institute of Ecosystem Studies, Riverkeeper, and municipalities that use the Hudson River as a water source, meets monthly to discuss this issue. However, a formal study of changes to salt front migration in the Hudson River due to sea level rise has yet to take place.

#### 7.1.3 Assessment of confidence based on evidence

Existing data trends, modeling efforts, and studies indicate with **high** confidence that water bodies and groundwater under the influence of sea level have been and will continue to be impacted by climate change. However, the magnitude and extent of impacts to New York State's potable water supplies due to rising sea levels **remains uncertain**. Basic physical processes lead to saltwater intrusion in aquifers when less dense fresh water is displaced by denser salt water. However, there needs to be a comparison of the actual geographic position of water wells, the populations served by these wells, and the effect of pumping of these wells in conjunction with groundwater rise. Similarly, the position of the salt front in estuarial rivers is dictated by the balance between freshwater outflows and the downstream elevation and salinity of the ocean. However, the salt front also depends on river bathymetry and freshwater withdrawals (factors in part controlled by humans), and its impact on water supply depends on operational adaptability, such as the capacity to draw more water during low tide, desalinate brackish water, or mix river water with alternate sources of water.

Given the relatively tractable nature of the processes and controls involved, there is **high** confidence that additional modeling and analysis can provide improved, actionable insight into saltwater intrusion into aquifers and the movement of the salt front in estuarial rivers.

### 7.2 Key Finding 2

**New York State's aging water infrastructure is particularly vulnerable to the impacts of climate change**. New York's older infrastructure was built for water levels, flows, and water quality conditions that have since changed and will continue to change. Older infrastructure is also more prone to leaks, breaks, and other failures. Upgrades to aging infrastructure can offer the dual benefit of building resilience for the future and providing immediate improvements in water system function. Strategically identifying upgrades that also support climate adaptation can be a cost‐effective way to allocate limited resources.

#### 7.2.1 Description of evidence base

The evidence base describing the aging of New York's water infrastructure is extensive. Many publications, including peer‐reviewed journals, reports from the New York State and New York City governments, and news articles, address infrastructure age and condition.^8^, ^11^, ^36^, ^311^, ^312^ The state's 20‐year infrastructure needs were recently estimated at approximately $44.2 billion for drinking water infrastructure and $38 billion for wastewater infrastructure.^36^ Many publications, including peer‐reviewed literature and news articles, have also cited aging and deteriorating water infrastructure as a nationwide concern and vulnerability.^212^, ^213^, ^214^

The effects of climate change on already deteriorating and vulnerable water infrastructure are also well documented, although much of this documentation is not focused on New York. Peer‐reviewed journals, state and federal government studies, and news reports consistently agree that increasing frequency and severity of storm events, rising sea levels, and increasing atmospheric temperatures will exacerbate stresses on New York's water infrastructure.^8^, ^215^, ^216^, ^245^, ^389^, ^390^

Many publications address the risk of increased inundation of water and wastewater treatment facilities due to precipitation and/or storm surges,^216^, ^244^, ^311^, ^389^, ^390^, ^391^, ^392^ as well as increased need for additional water treatment following severe weather events.^215^, ^392^ The likely increase in episodes of CSOs and sanitary sewer overflows due to the age of sewer systems is also well documented and expected to continue across the state.^216^, ^392^ Other publications highlight the growing need for backup power sources.^244^, ^390^

There are multiple anecdotal examples of how changing climate hazards have already led to changing water infrastructure design. Sea level rise was a factor for the city of Albany's new design and increased construction costs of tide gates at its Hudson River CSO outfall.^278^ Albany's climate change vulnerability assessment acknowledges that the two WWTP facilities near the Hudson River could experience more frequent flooding due to sea level rise and more frequent precipitation.^279^ Kirchhoff and Watson note that wastewater treatment facilities in Connecticut are already affected by sea level rise; facility managers have had to employ protective measures, including floodgates, flood‐proof doors, and plugs in vent holes.^244^

It is estimated that if New York City invested $315 million in fortifying and updating essential wastewater infrastructure, the city could avoid more than $2 billion in anticipated damages from storm surge, flooding, and sea level rise.^377^

#### 7.2.2 New information and remaining uncertainties

While scientists have very high confidence that heavy precipitation events will increase in frequency and severity (refer to projections developed for this assessment),^72^ the exact rate of increase in flooding is not as certain. It is expected that 100‐year floods will occur more frequently due to climate change, especially in coastal regions where the sea level is rising. A recent study of coastal flooding estimates that by the late 21st century, the 100‐year flood level could occur as frequently as annually in the northeastern United States.^218^ Estimates have changed greatly in the past decade and will likely change more as the climate changes, and standard sources and their underlying data on site‐specific precipitation events may no longer be appropriate for informed decision‐making around infrastructure investments due to underestimation of precipitation frequency estimates.^393^

Projected rates of sea level rise are better understood, albeit with a wide range of climate scenarios and uncertainty about future rapid ice melt. The projections developed for this assessment show sea level rising along New York's coast by up to 1 foot by the 2030s, about 2–3 feet by the 2080s, and more than 4 feet by 2150, relative to a 1995–2014 baseline.^72^

#### 7.2.3 Assessment of confidence based on evidence

There is **very high** confidence in the relationship between infrastructure age and repair or replacement needs and climate impacts. This level of confidence is based on the well‐documented concerns regarding the current age and condition of water infrastructure in the state, the likelihood that the condition of aging infrastructure will be exacerbated by the impacts of climate change, and the location of infrastructure in floodplains and other high‐impact areas throughout the state. The evidence supports **high** confidence in the finding that additional investments will be required to adequately address impacts such as changing water quality and decreased reliability of services from grid‐based electrical disruptions.

### 7.3 Key Finding 3

**Resources and preparedness for dealing with climate change vary greatly depending on the size and wealth of different communities**. Water infrastructure and water resources management are largely overseen by local governments. For communities that cannot afford full‐time technical staff, several different resources are available from state and federal governments and nongovernmental organizations, but these resources can be difficult and time‐consuming to navigate. There is a need to develop more efficient and less burdensome ways to support communities in implementing resilience measures.

#### 7.3.1 Description of evidence base

The recognition of disparities in resource availability at the municipal level emerged from comments by this assessment's Sector Advisors and from interviews with NYSDEC staff involved with floodplain management. This point is also consistent with anecdotal observations of resilience planning in the state. For example, NYC DEP operates one of the world's most complex water and wastewater systems, serving more than 9.5 million people.^394^ At the opposite end of the spectrum are small municipalities serving hundreds to thousands of people that may have only one or two staff members, mainly tasked with day‐to‐day water system operation.

The academic literature corroborates these findings, albeit without a specific focus on New York. In a study from Houston, Texas, less affluent communities were found to make fewer capital investments following water quality violations than more affluent communities.^395^ A study of disparities between urban and rural areas across the United States found that urban areas received much more hazard mitigation assistance per capita than rural areas.^396^

Numerous examples illustrate the availability of resources to support small, low‐income municipalities in water infrastructure management and planning. For example, the New York State Environmental Facilities Corporation specifically provides engineering planning grants of up to $100,000 for communities with median household incomes below certain thresholds.^397^ The New York State Floodplain and Stormwater Managers Association continually promotes training and certification for people tasked with making local floodplain management decisions.^398^ Similarly, New York State's Drinking Water Source Protection Program launched in 2021^399^ and began to offer free technical advice to municipalities to update plans to protect local water supplies. Guidance from the program indicates that municipalities can use the updated plan to apply for funding from more than 20 federal and state sources. Possible sources of assistance include the Climate Smart Communities program and the Water Quality Improvement Project program.^400^

While there is little peer‐reviewed documentation of difficulties in navigating these many programs, there are anecdotal examples such as the role the Ausable River Association has played in helping the Keene, Upper Jay, and Au Sable Forks communities plan and fund modifications to the river channel within their boundaries. Refer to the case study Ausable River Resilience Planning for more information.

#### 7.3.2 New information and remaining uncertainties

A study^378^ of the multitude of financing structures used to fund water infrastructure—bonds, state revolving funds, federal loans, and public‐private partnerships—reflects the complicated landscape of making capital investments necessitated by the high degree of fragmentation and mix of governance arrangements for water utilities. Academic literature evaluating understanding of the function of small water systems^379^ has documented causes and impacts of fragmentation among water utilities but has notably not considered consequences in the context of new stressors brought by climate change.

A disparity in resources might not be the sole cause of a lack of resilience planning. There could also be a lack of acknowledgment of climate change in some communities due to the politicization of climate change. Additionally, the technical path to resilience is not always clear (unlike a more straightforward municipal project such as building a road or bridge), and this lack of clarity in accepted methods or approaches could limit effective resilience planning.

#### 7.3.3 Assessment of confidence based on evidence

Given the consistency in evidence from multiple sources, there is **high** confidence in the conclusion that there is a disparity in resources across municipalities and that new ways to support communities in developing resilience to climate change should be considered.

### 7.4 Key Finding 4

**Long‐term water infrastructure resilience requires the proactive incorporation of changing climate conditions into planning and design**. State, Tribal, municipal, and other responsible entities broadly recognize that new infrastructure should reflect knowledge of future climate conditions to avoid increasing the risk of failure. However, new analytical tools, models, and data sets need further development to provide a basis for establishing forward‐looking standards. Continued support of applied science research and open‐access water data will help advance these improvements.

#### 7.4.1 Description of evidence base

Multiple water resource–related programs have started to update standards to reflect changes in risk due to a changing climate. Passage of the New York State Climate Risk and Resiliency Act in 2014 led to new guidance on assessing flood risk and has called for flood zones to be designated based on the standard 2–3 feet of freeboard for critical facilities added to existing 100‐year floodplain elevations.^180^ The *New York State Stormwater Design Manual* ^401^ has been updated to require the use of precipitation data from recent decades (e.g., Atlas 14^303^) instead of the mid‐20th century. Organizations managing water withdrawals such as the DRBC and the New York State Department of Environmental Protection have developed watershed modeling programs that use ensembles of future climate model predictions. (Refer to the case study Delaware River Basin Climate Collaboration for more information.) Passage of the Great Lakes Compact in 2012 led to new provisions for NYSDEC to permit water withdrawals by users with an average capacity greater than 100,000 gallons per day in any 30‐day period,^365^, ^366^ thus offering a new means to track competing water demands in the state. A number of approaches to flood adaptation have been tested in New York, including the conservation of natural features that protect against flooding (e.g., wetlands, forests), changes to land use regulations, and structural interventions (e.g., floodwalls and dams).^288^, ^402^ Future land use plans can also account for flooding by prohibiting development in high‐risk areas, thereby conserving essential natural features and protecting communities.^402^

There is limited academic literature focused on documenting changing impacts due to delays in updating standards. This limitation is well reflected in the body of literature evaluating the need for updates in stormwater infrastructure design standards. In most academic literature assessing the impacts of changes in precipitation on stormwater infrastructure, there is only a general presumption that as the frequency and intensity of actual storm events exceed that anticipated by designs based on historical weather, undersized stormwater infrastructure will lead to increased localized flooding and damages.^403^, ^404^, ^405^ The Technical Workgroup identified no academic studies that directly identified increased failure of stormwater infrastructure and increased damages due to climate change. Of those evaluations that do exist, the evidence for increased localized flooding and accompanying damage is mainly from simulations of urban stormwater systems.^406^ This lack of effort to conduct direct documentation of possible changing impacts is likely in part due to the well‐established physical controls that link increased precipitation intensities with overwhelmed stormwater infrastructure. In urbanized areas with a large fraction of impervious surface, increased precipitation intensities will increase runoff rates. Existing stormwater infrastructure—constrained by current pipe slopes and diameters—has firm physical maximums on the amount of runoff that can be conveyed before flooding occurs.

#### 7.4.2 New information and remaining uncertainties

The exact trajectory of changes in climate drivers remains uncertain, but regardless of the pace of change, the assessment in New York State's Changing Climate concludes with very high confidence that average temperature, the frequency and intensity of extreme heat events, sea level, annual total precipitation, and the frequency and intensity of heavy precipitation events will increase through the rest of the 21st century.^72^

In many cases, entities responsible for planning and design have recognized the need to replace old standards that are based on decades‐old climate data or hydrologic conditions. However, there is often a lack of resources to support new approaches. While the stormwater design manual suggests the use of Atlas 14, Atlas 14 uses data only through 2014.^301^ More forward‐looking approaches exist, including a series of intensity‐duration‐frequency curves specifically developed for New York State using nearly 50 downscaling simulations.^407^ In terms of tracking competing water demands for water supply, there are few resources for enforcement and monitoring to ensure water users actually report use under Environmental Conservation Law Article 15 Title 15, especially by temporary users who may only draw water during dry periods.

A remaining primary uncertainty is the lack of direct observation and documentation of localized impacts due to the use of outdated standards related to stormwater. The transient and small‐scale nature of some impacts makes them difficult to document.

#### 7.4.3 Assessment of confidence based on evidence

It is **well established** that old standards used in the design of water infrastructure depended on climate data from decades ago. Efforts to develop new standards have occurred transparently within the public domain with new programs or guidelines broadly announced (e.g., New York's Community Risk and Resiliency Act). There are remaining knowledge gaps, which are well communicated, with agencies expressing the need for new approaches to ultimately replace interim methods (i.e., the application of margins of safety instead of standards based on high‐quality climate projections). There is **high** confidence in the need to remain proactive in incorporating new information on changing climate conditions into standards.

### 7.5 Key Finding 5

**Climate change could place new stresses on already complex, multijurisdictional water governance, especially during drought periods**. A variety of in‐state and regional organizations manage these multijurisdictional waters for flood protection water supply, electric generation, recreation, and other uses. These complex governance structures affect underserved communities, regional governments, Tribal Nations, and other entities that are particularly vulnerable to negative climate impacts and that often have fewer resources and influence to represent their interests. Water management approaches that incorporate these entities’ participation and diverse knowledge, including Indigenous Traditional Ecological Knowledge, can help remedy water injustices and identify new opportunities to increase climate resilience.

#### 7.5.1 Description of evidence base

Many publications address the inherent complexity and scale of water governance,^408^, ^409^, ^410^, ^411^, ^412^ particularly in the context of climate change.^411^, ^413^, ^414^, ^415^ The literature indicates that climate change exacerbates pre‐existing governance and management challenges and furthers existing injustice and inequality. Conversely, poor water resource management increases vulnerability to climate change–related stressors and compounds environmental justice concerns.^413^ Because stationarity of climate can no longer be assumed, adaptive governance is imperative.^415^

Water justice has emerged as a standalone subset of environmental justice research.^52^, ^416^ In recent years, the focus of water management has shifted to include social, environmental, and economic concerns, including water justice and climate resilience. The literature underscores the importance of including diverse voices and viewpoints in water justice conversations.^52^

The extent and impacts of water injustice on Indigenous Peoples and Tribal Nations are well documented.^314^, ^316^, ^417^ In New York, disparities between Tribal and non‐Tribal water systems are evident. Tribal systems are more likely to be affected by MCL violations than non‐Tribal systems.^314^ Federal financial support for Tribal water systems, including funding for compliance, is lacking.^315^

The literature indicates that the effectiveness of oversight and governance of water resources is enhanced when all people can participate,^418^ and that underserved communities’ active participation in water governance is imperative to achieving water justice in a changing climate.^408^ Integrating Indigenous Traditional Ecological Knowledge into water governance and adaptation practices can provide a deeper, more specific understanding of the geographic region and associated ecosystems, which can enhance holistic watershed and ecosystem‐based management targets.^419^ Transparency in water governance, as well as meaningful inclusion of those most affected by water injustice in decision‐making, will increase resilience in a time of great uncertainty.

#### 7.5.2 New information and remaining uncertainties

Much of the literature cited regarding the challenges associated with water governance focuses on global issues, rather than issues specific to New York. It is reasonable to assume that the problems affecting other developed nations are also of concern in the United States. However, due to the lack of regional specificity in the literature, the extent to which water governance challenges are widespread in the state is still uncertain.

Similarly, information specific to New York's Tribal Nations is limited. Literature discussing nationwide environmental and water injustice issues on Tribal lands is available, but data specific to the Tribal Nations across New York is minimal. Therefore, water governance and justice challenges affecting New York's Tribal Nations require further community‐engaged and codesigned investigations.

#### 7.5.3 Assessment of confidence based on evidence

There is **high** confidence in the general conclusion that climate change could add stress to already complex water governance challenges. Scholarship on this topic consistently highlights ways in which climate change exacerbates existing challenges. However, because much of the available evidence comes from outside New York State, including from other regions and other countries with higher drought risk and more severe constraints on water supply, there is **medium** confidence in this conclusion as applied specifically to New York.

The idea that some groups are both more vulnerable to climate change impacts and have less political and financial power to assert their right to equitable treatment is widely established, with supporting evidence across many disciplines, including water governance. This portion of the key finding has **very high** confidence. There is **high** confidence that ensuring the representation of overburdened communities and Indigenous Peoples in water governance will lead to markedly more just and resilient outcomes.

## AUTHOR CONTRIBUTIONS

K.L.: Drafting, revising, and editing the manuscript; general supervision. S.B.S.: Drafting, revising, and editing the manuscript; drafting Kingston WWTP case study; general supervision. A.F.: Drafting, revising, and editing content related to Tribal Nations and water resources; drafting Akwesasne case study. D.H.: Drafting, revising, and editing sections related to water governance; drafting Ausable River Association case study. L.J.: Drafting, revising, and editing content related to hydrologic modeling; drafting DRBC case study. C.L.M.: Drafting, revising, and editing content related to groundwater. B.W.: Drafting, revising, and editing sections related to water and wastewater treatment. K.Y.: Drafting, revising, and editing sections related to water quality.

## COMPETING INTERESTS

The authors declare no competing interests.

### PEER REVIEW

The peer review history for this article is available at: https://publons.com/publon/10.1111/nyas.15197
